# Impact-acceleration head injury results in optic neuropathy in the thirteen-lined ground squirrel

**DOI:** 10.1038/s42003-026-10397-4

**Published:** 2026-06-09

**Authors:** Francisco M. Nadal-Nicolás, Eve Gold, Amanda Fu, Yeonho Kim, Yue Gao, Joseph McCabe, Kiyoharu J. Miyagishima

**Affiliations:** 1 National Eye Institute, NIH, Bethesda, MD USA; 2 Department of Ophthalmology, Faculty of Medicine, University of Murcia and Biomedical Research Institute of Murcia (IMIB-Pascual Parrilla), Murcia, Spain; 3 National Institute of Mental Health, NIH, Bethesda, MD USA; 4 Preclinical Behavior and Modeling Core, Department of Laboratory Animal Resources, Uniformed Services University of the Health Sciences, Bethesda, USA; 5 Henry M. Jackson Foundation for the Advancement of Military Medicine Inc., Bethesda, MD USA; 6 National Institute of Dental and Craniofacial Research, NIH, Bethesda, MD USA; 7 Department of Anatomy, Physiology & Genetics, F.E. Hébert School of Medicine, Uniformed Services University of the Health Sciences, Bethesda, MD USA

**Keywords:** Retina, Stress and resilience

## Abstract

Traumatic brain injury (TBI) caused by rapid head acceleration produces visual impairments from damage to the optic nerve (ON), as well as widespread perturbations of the central visual system. Widely used mouse and rat models have limitations in translational relevance due to their nocturnal habits and rod-dominant retinas. In contrast, the thirteen-lined ground squirrel (Ictidomys tridecemlineatus) is diurnal and possesses a cone-dominant retina with high retinal ganglion cell (RGC) density and an interlocking astrocyte pattern in the nerve fiber layer—an anatomical feature shared with primates and humans but not observed in other common rodent models. These similarities make the thirteen-lined ground squirrel uniquely suited for modeling the complex, diffuse pathophysiology of human closed-head TBI. Using a repetitive closed-head impact paradigm, we establish the thirteen-lined ground squirrel as a translational model of TBI. Repeated impacts induce long-term pathology, including abnormal seasonal weight regulation and persistent visual dysfunction. Longitudinal ophthalmic assessments reveal retinal thinning and significant reductions in RGC function. Histological analysis confirms dorsal RGC loss and late-stage changes in ON glia. Together, these findings demonstrate that the thirteen-lined ground squirrel recapitulates systemic, behavioral, and visual dysfunction following TBI, establishing it as a promising model for neurotrauma and vision loss.

**Figure:**
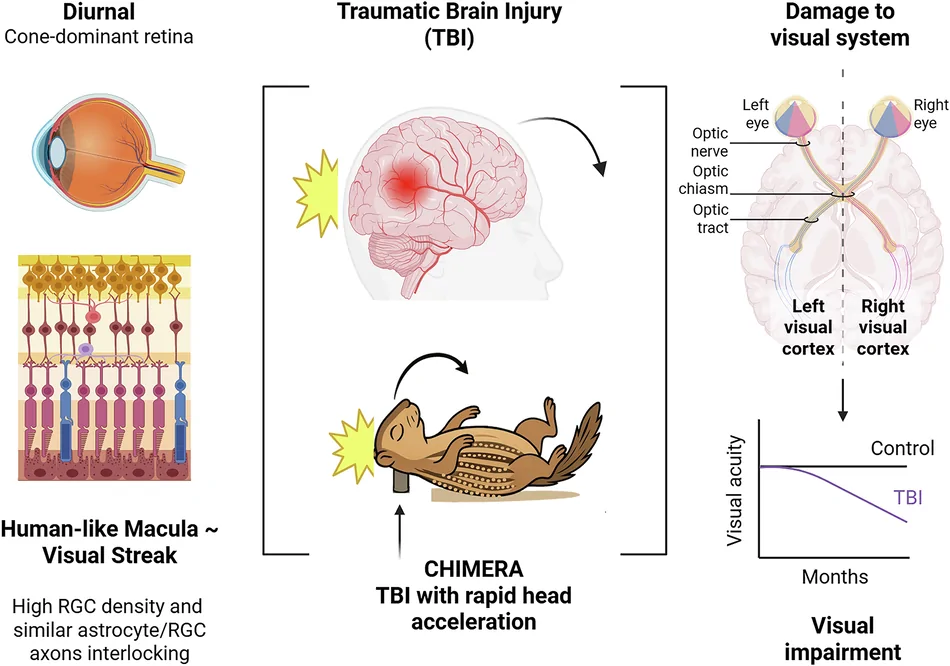

## Abstract (web summary)

Repetitive CHIMERA impacts induce visual, retinal ganglion cell, optic nerve, and metabolic dysfunction in the thirteen-lined ground squirrel, recapitulating key features of human traumatic brain injury and suggesting that this squirrel could be a useful future model for studying neurotrauma and vision loss.

## Introduction

Falls or collisions involving the head can cause traumatic brain injury (TBI). Visual impairment is a frequent consequence, comprising 20–40% of clinical cases^1^. Symptoms include reduced visual acuity, visual fields, contrast sensitivity, and pupil light reflex, deficits in dark adaptation, and double vision^2–9^. Visual dysfunction is particularly prevalent in military personnel exposed to blast-related injuries and in individuals with sports-related concussions^10–12^. Despite this high prevalence, the mechanisms underlying TBI-induced visual dysfunction remain incompletely understood, in part because current animal models do not fully capture clinically relevant pathophysiology.

We hypothesized that thirteen-lined ground squirrels (*Ictidomys tridecemlineatus*), due to their larger size and unique retinal architecture^13–15^, may provide a more suitable model for studying TBI than traditional rodents. Like humans, the thirteen-lined ground squirrel is diurnal and possesses a cone-dominant retina that supports high visual acuity during daytime activity^16–18^. Comparable to the human macula, which contains a high concentration of cones, the thirteen-lined ground squirrel has a visual streak located approximately 2 mm below the optic nerve (ON) head, where cone density peaks at ~50,000/mm² ^13^. This anatomical specialization enhances spatial resolution across the horizontal visual field, facilitating predator detection and navigation in open habitats.

In addition, the visual streak of the thirteen-lined ground squirrel possesses a high ganglion cell (RGC) density partially distributed across multiple layers, resembling primate retinal organization^14–19^. Notably, it also exhibits an interlocking astrocyte pattern in the nerve fiber layer shared with primates but not observed with other commonly used rodent models^14,19^. These characteristics make the visual streak comparable to the human macula and establish the thirteen-lined ground squirrel as a uniquely suitable model for studying the complex, diffuse pathophysiology of human closed-head TBI^20–22^.

Hibernators like the thirteen-lined ground squirrel, in particular, exhibit a remarkable resistance to injury during torpor^23^. This includes natural tolerance to ischemia and hypoglycemia^24–28^, as well as an inherent ability to reverse cataract formation^29^, offering a unique opportunity to investigate endogenous protective and regenerative mechanisms. However, while torpor is merely one aspect of hibernation physiology^30^, for this study, we chose to focus exclusively on euthermic animals that did not enter torpor, allowing us to examine the anatomical and functional consequences of TBI in a nonhibernating physiological state. Moreover, because thirteen-lined ground squirrels display pronounced seasonal cycles of weight gain and loss, they serve as a valuable model for studying obesity and metabolic regulation^31–34^, providing a unique framework for investigating how TBI may disrupt systemic metabolic homeostasis.

In this study, we sought to characterize visual impairment and alterations in the primary visual pathway induced by the Closed Head Impact Model of Engineered Rotational Acceleration (CHIMERA) model of TBI in thirteen-lined ground squirrels. Because TBI is defined by a concussive head impact, and vision originates in the retina before being transmitted to central brain targets, we focused our analyses on key structures along the primary visual pathway. In particular, we examined RGCs—the sole afferent neurons of the retina that project to the brain by forming the ONs—as well as the ONs themselves, where axonal injury, neuroinflammatory responses, and glial alterations are known to contribute to visual dysfunction^35,36^. Concussive events can induce compression, stretching, and dynamic shear forces within ONs, rendering these white matter axons particularly vulnerable even in the absence of direct ocular or ON trauma^6,8^. By assessing both retinal and optic nerve pathology, we aimed to identify structural correlates of functional and behavioral visual deficits associated with CHIMERA-induced TBI in a diurnal rodent with high visual acuity^16^, and to explore potential mechanisms of neuroprotection relevant to human traumatic brain injury.

## Results

### Establishing the thirteen-lined ground squirrel as a model of traumatic brain injury

In our preliminary studies (Supplementary Information), a single impact of varying energies (0.5, 1, and 1.5 J) did not generate sufficient injury to produce lasting pathology, nor did consecutive impacts within 30 s of each other on the same day. After an iterative process of testing impact energy, timing, and type, we established a protocol in which each animal was subjected to two CHIMERA impacts at 1.5 J for 3 consecutive days. This approach generates rapid acceleration and rotational forces that induce inertial and physical contact stresses on the brain, eye, ON, and surrounding tissues^37–41^.

**Fig. 1:**
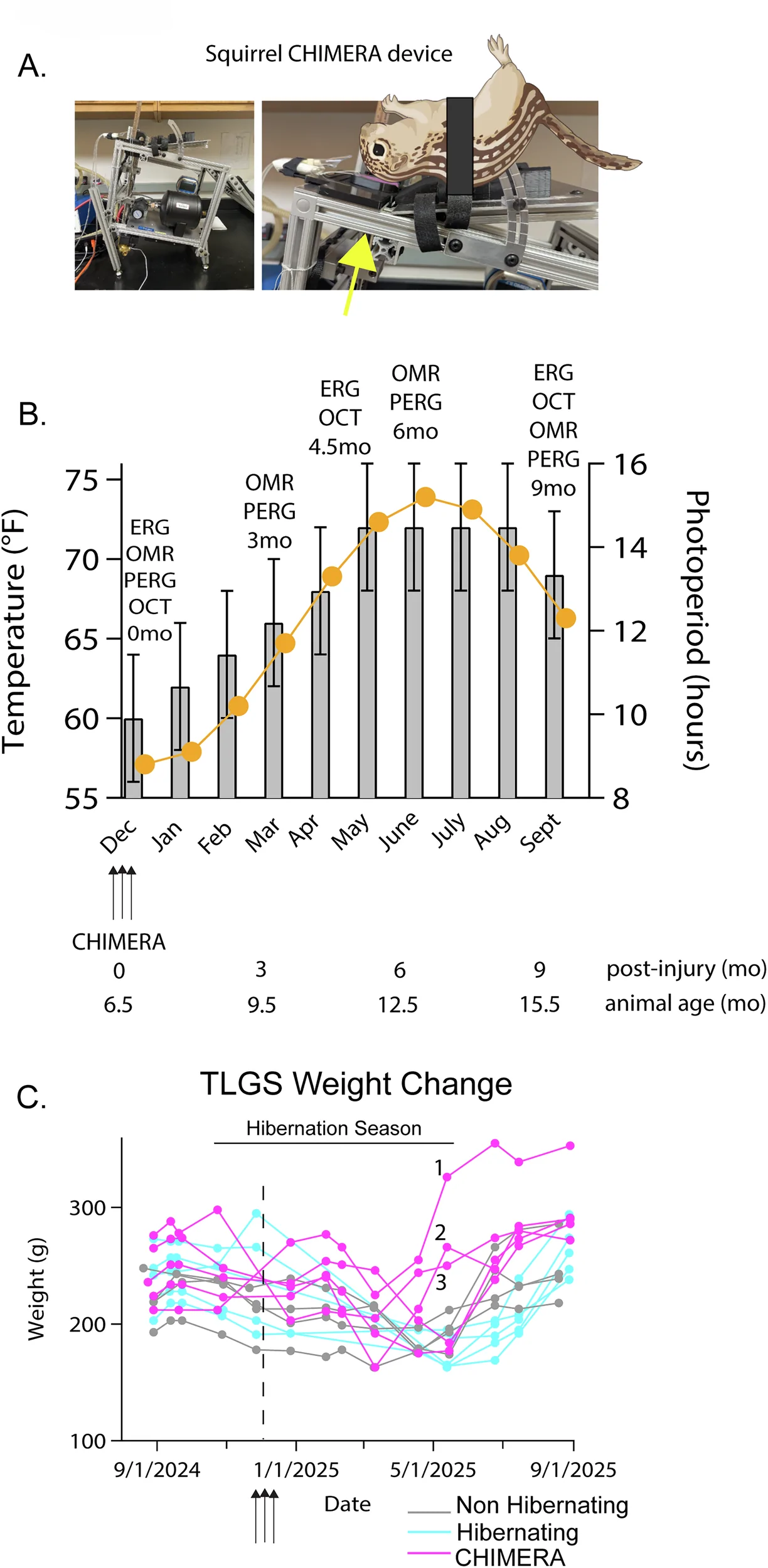
CHIMERA in the thirteen-lined ground squirrel. **A** Images of the CHIMERA apparatus used in this study. Left: horizontal view of the device, with the air compressor located beneath the platform. Right: squirrel positioned supine and secured with Velcro straps; a 3D-printed interface is placed between the animal’s head and the piston, which is aligned with the midline of the skull. A pressure of 6.1 psi corresponds to a piston velocity of 5.477 m/s (yellow arrow) and delivers an impact of 1.5 J. Each animal received two impacts, separated by 60–90 s, on 3 consecutive days. Created in BioRender. Miyagishima, K. (2026) https://BioRender.com/6cd89j6. **B** Experimental timeline showing the time points for visual function and structural assessments, as well as the environmental temperature (gray bar plot) and photoperiod (orange trace) the animals were housed. Error bars represent ±4 °F corresponding to the monthly upper and lower temperature range of the squirrel holding room. **C** Monthly body weights over 1 year in nonhibernating controls (gray, *n* = 4 animals), nonhibernating CHIMERA-exposed animals (magenta, *n* = 5 animals), and animals allowed to hibernate (cyan, *n* = 5 animals, as a reference for body weight changes during hibernation).

To reduce direct skull damage, an interface consisting of a 3D-printed frame lined with moldable silicone putty was placed between the piston and the animal’s head (Fig. 1A, right). The piston’s upward velocity was controlled by adjusting the pressure of compressed air released from a tank positioned beneath the apparatus (Fig. 1A, left). The interval between the two impacts was approximately 60–90 s, allowing time for repositioning and to ensure complete anesthesia. A timeline of the experiment and the time points when the animals were noninvasively assessed for visual function (electroretinogram and pattern ERG), behavioral (optomotor response, OMR), and structural changes optical coherence tomography, (OCT) is provided in Fig. 1B. Also included in the diagram for reference are the ambient housing temperatures, which were adjusted incrementally throughout the year, and the photoperiod, which was set to match sunrise and sunset times in Oshkosh, Wisconsin and adjusted every two weeks to mimic natural seasonal conditions.

The experimental paradigm was selected to reflect the complexity of a variety of human injury scenarios, including repetitive impacts sustained in contact sports, falls, military operations, and domestic abuse^42,43^.

### CHIMERA disrupts seasonal weight cycling in the thirteen-lined ground squirrel

Thirteen-lined ground squirrels are a naturally valuable model for studying obesity and metabolism due to their pronounced seasonal cycles of weight gain and loss. During the summer, squirrels accumulate fat reserves, which are then utilized over the winter months while hibernating. This annual cycle provides a unique framework for investigating how traumatic brain injury (TBI) can alter metabolic regulation.

We monitored squirrel body weights monthly and body temperature continuously after their arrival from Wisconsin (Fig. 1C). Because not all animals in our facility are observed to show signs of torpor, which would have them placed into a hibernaculum during the hibernation season, we selected nonhibernating animals for this study to isolate the metabolic effects of TBI without the potential confounding neuroprotective influence of cold associated with hibernation^44^.

For body weight comparison, we included a group of thirteen-lined ground squirrels that hibernated during the same period for comparison. Strikingly, three of the five thirteen-lined ground squirrels, maintained in euthermic conditions and subjected to CHIMERA in early December exhibited extreme fluctuations in increased body weight (numerically annotated) that were uncoordinated with their expected seasonal cycle (May 13, 2025), suggesting a whole-body metabolic disruption induced by TBI (Fig. 1C). Because these three animals appeared to reach near-maximal weights earlier than anticipated, we assessed spleen and kidney weight, as enlargement of these organs is commonly associated with obesity-related body composition in humans, and reported spleen size changes after TBI^45^. However, average spleen weights in TBI animals (0.18 g ± 0.05, *n* = 5 animals [3 male and 2 female]) were not significantly different from those of control animals (0.24 g ± 0.1, *n* = 4 [2 male and 2 female]; Supplementary Information; unpaired two-tailed t-test: *p* = 0.2988; Supplementary Data 1). Similarly, kidney weights did not differ appreciably between naïve (0.80 g ± 0.08, *n* = 8, two kidneys per animal, [4 and 4 from male and female animals, respectively]) and TBI animals (0.84 g ± 0.14, *n* = 10 kidneys [6 and 4 from male and female animals, respectively]), with no statistical difference detected (Supplementary Information; unpaired two-tailed t-test: *p* = 0.4650; Supplementary Data 1).

### CHIMERA induces transitory changes in retinal thickness

**Fig. 2:**
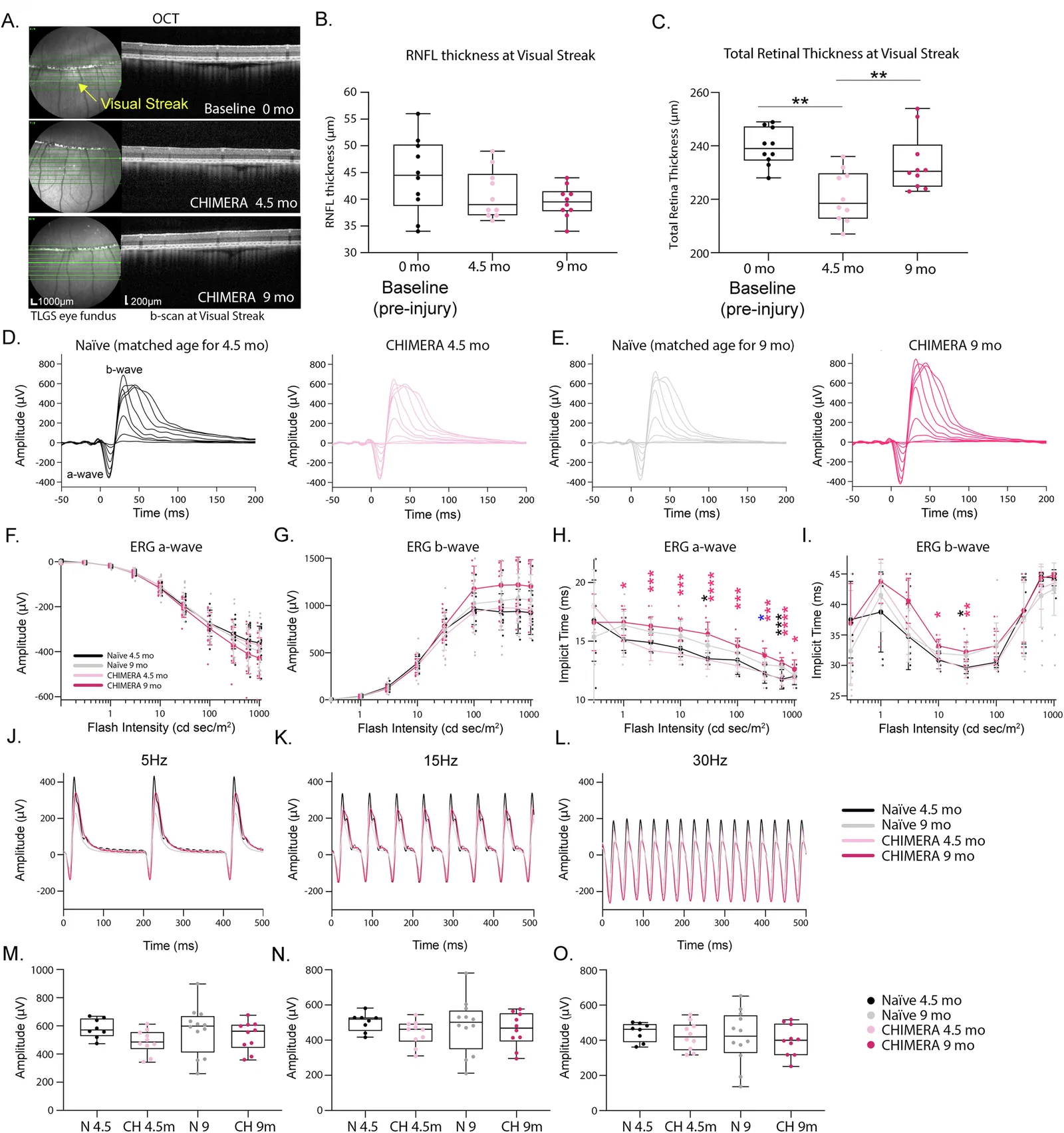
OCT and ERG measurements in thirteen-lined ground squirrels before and after CHIMERA. **A** Representative OCT images (eye fundus and b-scans) from a thirteen-lined ground squirrel examined at baseline (pre-injury), 4.5- and 9- months post-injury. Yellow arrow indicates the visual streak, located ~2 mm beneath the optic nerve head. Box plots showing changes in retinal nerve fiber layer (**B**) and total retinal thickness (**C**) before (baseline) and post-CHIMERA. Significant changes are indicated by asterisks (*n* = 10 eyes [6 and 4 from male and female animals, respectively], repeated-measures one-way ANOVA and Tukey post hoc test, **p ≤ 0.01). **D**, **E** Averaged ERG responses for uninjured controls (*n* = 8 eyes from 4 animals) and CHIMERA-injured animals (*n* = 10 eyes from 5 animals) at 4.5 months (**D**) and 9 months (*n* = 12 eyes from 6 separate naïve animals). **E** Post-injury (*n* = 10 eyes from 5 animals). Response amplitude versus stimulus intensity for a-waves (**F**) and b-waves (**G**). Implicit times of a-waves (**H**) and b-waves (**I**). Sample size for CHIMERA-injured animals (same animals analyzed at 4.5 and 9 month post injury), *n* = 10 eyes, 5 animals [3 males and 2 females]; age-matched naïve controls at the 4.5-month, *n* = 8 eyes, 4 animals [2 males and 2 females]); and age-matched naïve controls at 9-month, *n* = 12 eyes, 6 animals [4 males and 2 females] statistical comparisons were performed using ordinary one-way ANOVA followed by Tukey’s post hoc test. Dark magenta asterisks indicate differences between CHIMERA at 4.5 and 9 months; blue asterisks indicate differences between CHIMERA at 9 months and naïve at 9 months; black asterisks indicate differences between naïve at 4.5 and 9 months. Significance levels are denoted as follows: ****p ≤ 0.0001, **p ≤ 0.001, *p ≤ 0.01, and *p* ≤ 0.05. **F**–**I** Error bars represent standard deviation. Averaged light-adapted flicker ERG responses to 5 Hz (**J**), 15 Hz (**K**), and 30 Hz (**L**) stimuli in naïve and CHIMERA-injured animals (same cohort). Box plots of flicker response amplitudes at 5 Hz (**M**), 15 Hz (**N**), and 30 Hz (**O**) show no significant differences among the analyzed groups (ordinary one-way ANOVA). See Supplementary Data 1 for statistical details. The whiskers on all boxplots shown (**B**, **C**, **M**–**O**) extend to the 90th (upper) and 10th (lower) percentiles.

Since repetitive head impacts can result in optic neuropathy and functional impairments^46^, we evaluated longitudinal changes in retinal structure and function. We performed OCT and recorded light-adapted electroretinograms (ERGs) in control and injured animals at 4.5- and 9-month post-injury (Fig. 2).

Because ON injury is expected to reduce the number of RGCs, we examined thinning of the retinal nerve fiber layer (RNFL) as an indicator of axonal loss. However, RNFL thinning was not anticipated within the timeframe of this study, as previous studies have shown that retinal thickness in thirteen-lined ground squirrels remains stable with aging^47^. OCT measurements were therefore focused on the visual streak, a specialized retinal region characterized by the highest density of cones and RGCs. Importantly, due to the horizontal orientation of the ON head (ONH), which effectively divides the retina into superior and inferior hemiretinas, a large proportion of RGC axons from regions below the ONH converge through the visual streak^14–19^. This makes it a particularly reliable and anatomically consistent region for detecting changes in RNFL thickness in the thirteen-lined ground squirrel^14^.

Analysis of the visual streak revealed that RNFL thickness (Fig. 2A, *yellow arrow*) was not significantly reduced in injured animals at 9 months post-CHIMERA compared to the pre-injury (baseline) levels from the same animals (Fig. 2B**;** *n* = 10 eyes [6 and 4 from male and female animals, respectively], repeated-measures one-way ANOVA, *F*(1.389, 12.50) = 2.075, *p* = 0.1730; Supplementary Data 1). In contrast, total retinal thickness was significantly reduced at 4.5 months post-injury (Fig. 2C; same cohort, repeated-measures one-way ANOVA, *F*(1.946, 17.51) = 15.34, *p* = 0.0002; Tukey’s post hoc for multiple comparisons, *p* = 0.0014; Supplementary Data 1). By 9 months post-injury, retinal thickness was no longer significantly different from baseline but was significantly increased relative to the 4.5-month time point (Fig. 2C; Tukey’s post hoc for multiple comparisons, *p* = 0.0079; Supplementary Data 1).

### Progressive retinal degeneration following CHIMERA revealed by ERG analysis

To determine whether visual function was affected by CHIMERA, we evaluated the entire visual pathway, beginning with the photoreceptors and bipolar cells. Full-field electroretinography (ERG) was performed using corneal electrodes, where the a-wave reflects photoreceptor activity, and the b-wave reflects bipolar cell activity. Averaged ERG responses from naïve thirteen-lined ground squirrels, at 4.5- or 9-month post-CHIMERA, are shown in Fig. 2D. Averaged ERG responses from thirteen-lined ground squirrels at 4.5 months post-injury and age-matched naïve animals are shown in Fig. 2D; whereas Fig. 2E shows responses at 9 months post-injury alongside age-matched naïve animals.

Neither the ERG a-wave nor the b-wave showed significant changes in amplitude (Fig. 2F, G) in CHIMERA-injured animals (*n* = 10 eyes, 5 animals [3 males and 2 females]) compared with age-matched naïve controls at the 4.5-month (*n* = 8 eyes, 4 animals [2 males and 2 females]) and 9-month (*n* = 12 eyes, 6 animals [4 males and 2 females]) post-injury time points (nonsignificant ordinary one-way ANOVA and Tukey’s post hoc for multiple comparisons test, see Supplementary Data 1). However, implicit time for a-wave for most of the stimulus conditions, and only punctually in b-wave (Fig. 2H, I), showed a significant increase at 9 months post-CHIMERA with respect to 4.5 months post-CHIMERA (same cohort, ordinary one-way ANOVA and Tukey’s post hoc for multiple comparisons test, see Supplementary Data 1 for details). Regarding implicit time changes, we also observed sporadic differences between naïve, 4.5-month, and 9-month groups; however, these were of much smaller magnitude and less consistent than those observed following CHIMERA injury (see Supplementary Data 1 for details). Together, these results indicate that CHIMERA injury does not measurably affect outer retinal response amplitude, but is associated with a subtle, time-dependent slowing of phototransduction kinetics.

The squirrel’s cone-dominant retina is specialized for rapid visual signal transmission. To evaluate cone-mediated visual function (Fig. 2J–L), we used flicker stimuli (10 cd s/m² at 5, 15, and 30 Hz) under light-adapted conditions (25 cd/m², white 6500 K) to suppress rod input. Because rod responses are not expected to contribute at frequencies above 28 Hz, the measured signals at 30 Hz (Fig. 2L) primarily reflect cone activity^48^. Post-injury animals showed no frequency-dependent changes in response amplitude compared with naïve animals even at 30 Hz (Fig. 2M–O; same cohort, ordinary one-way ANOVA; 5 Hz: *F*(3, 36) = 1.221, *p* = 0.3334; 15 Hz: *F*(3, 36) = 0.5753, *p* = 0.6350; 30 Hz: *F*(3, 36) = 0.3016, *p* = 0.8240, Supplementary Data 1), indicating that cone responses are not impaired by the mild closed head impact used in this study.

### CHIMERA-induced TBI causes subtle deficits in visual acuity measured by optomotor response

**Fig. 3:**
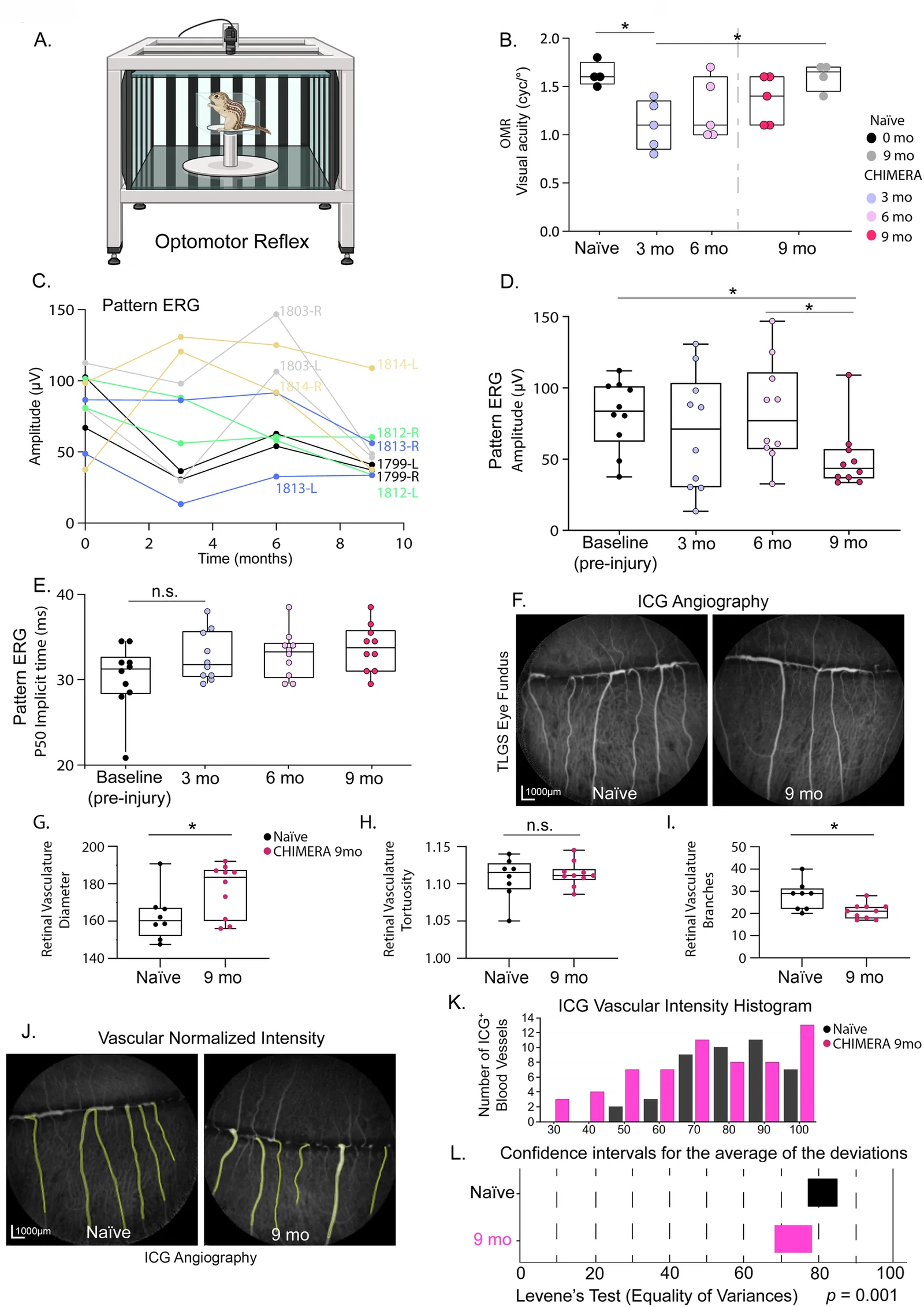
Visual function and retinal vasculature following CHIMERA in thirteen-lined ground squirrels. **A** Schematic of optomotor reflex (OMR) recording using the modified platform (see “Material and Methods” section). Created in BioRender. Miyagishima, K. (2026) https://BioRender.com/bm4zwp6. **B** Box plot showing changes in visual acuity at 3-, 6-, and 9-month post-CHIMERA compared to naïve and age-matched naïve controls (re-evaluated 9 months later). Statistical comparisons showing significant reduction in animals at 3 months post-CHIMERA (ordinary one-way ANOVA followed by Tukey’s HSD post hoc test, *p* = 0.03 with respect to naïve, and *p* = 0.04 with respect to age-matched naïve animals; *n* = 4 [2 males and 2 females], for naïve and age-matched naïve animals; and *n* = 5 animals [3 males and 2 females] re-evaluated at 3-, 6- and 9-months post-CHIMERA). Naïve OMR data reprinted from ref. ^95^ Fig. 5C Reprinted with permission. **C** Individual pattern ERG (PERG) response amplitudes before injury and at 3-, 6-, and 9-months post-CHIMERA. The colored traces indicate individual pattern ERG amplitudes from the left (L) and right (R) eyes of each thirteen-lined ground squirrel (black – animal ID-1799; gray – animal ID-1803; green – animal ID-1812; blue – animal ID-1813; yellow – animal ID-1814). **D** Box plot of PERG amplitudes demonstrating a significant reduction at 9 months post-injury relative to pre-injury levels (repeated-measurements one-way ANOVA with Tukey correction, *p* = 0.02), as well as a significant decrease in amplitude between 6- and 9-months post-injury (*p* = 0.03; *n* = 10 eyes [6 and 4 male and female eyes, respectively], the same animals were evaluated pre- and post-CHIMERA). **E** Box plot of P50 implicit times for PERGs indicating no statistical differences at 3-, 6-, or 9- months post-injury compared to baseline (same cohort, repeated-measurements one-way ANOVA with Tukey correction, *n* = 10 eyes, same as in (**D**). **F** Representative ICG angiography images of retinal vasculature in naïve controls and 9 months post-injury. Box plots of retinal vessel diameter (**G**), tortuosity (**H**), and the number of retinal vascular branches (**I**). Statistical comparisons were performed using unpaired two-tailed Student’s *t *tests (*n* = 8 [4 male and female] and 10 [6 and 4 male and female, respectively] eyes for naïve and 9-month post-CHIMERA groups, respectively): a significant reduction in vessel diameter (*p* = 0.0442) and branch number (*p* = 0.0109) was observed at 9 months post-injury (**G**, **I**), whereas no significant differences were detected in vascular tortuosity (**H**). **J** ICG angiography images showing vessels selected in yellow for pixel intensity analysis. **K** Histogram of ICG vascular intensity profiles, showing increased variability in animals 9 months post-injury compared to controls, which exhibit more uniform fluorescence intensity. **L** Plot of confidence intervals for naïve and 9-month post-injury animals; Levene’s test indicates greater variability in ICG intensity in post-injury animals (*p* = 0.001, same cohort: *n* = 8 [4 male and female] and 10 [6 and 4 male and female, respectively] eyes, naïve and 9-months post-CHIMERA, respectively). See Supplementary Data 1 for statistical details. The whiskers on all boxplots shown (**B**, **D**, **E**, **G**–**I**) extend to the 90th (upper) and 10th (lower) percentiles.

We also measured the optomotor response, which evaluates visual function through an involuntary head-tracking reflex to patterned stimuli (Fig. 3A). The optomotor test revealed a significant reduction in visual acuity at 3 months post-injury (1.10 ± 0.25 cyc/deg, *n* = 5 animals [3 males and 2 females]) compared to naïve animals (1.63 ± 0.13 cyc/deg, *n* = 4 animals [2 males and 2 females]; Fig. 3B; ordinary one-way ANOVA, *F*(4, 18) = 3.914, *p* = 0.0186; Tukey’s post hoc for multiple comparisons test, *p* = 0.0305; Supplementary Data 1). The difference in visual acuity at 6- (1.26 ± 0.32 cyc/deg) and 9-months (1.36 ± 0.25 cyc/deg) post-injury no longer reached statistical significance (Tukey’s post hoc for multiple comparisons test, *p* = 0.1989 and *p* = 0.4867 respectively; Supplementary Data 1). To account for potential age-related changes, the naïve animals (*n* = 4 [2 males and 2 females]) were re-evaluated 9 months later, and the visual acuity (1.6 ± 0.14 cyc/deg) showed no significant difference from the earlier time point (Tukey’s post hoc for multiple comparisons test, *p* = 0.9999; Supplementary Data 1). In contrast, CHIMERA animals at 3 months post-injury remained statistically different from controls, even when compared with the 9-month control measurements (Tukey’s post hoc for multiple comparisons test, *p* = 0.0418; Supplementary Data 1).

### Pattern ERG confirms delayed retinal ganglion cell dysfunction post-CHIMERA

To corroborate the visual deficits observed with the optomotor reflex, we also measured pattern electroretinography (PERG) in the same animals before injury and at 3-, 6-, and 9-months post-injury to track longitudinal changes in retinal function. PERG provides a measure of RGC activity, with reductions in amplitude reflecting loss of RGC function. At 9 months post-injury, TBI-exposed thirteen-lined ground squirrels showed a significant reduction in PERG amplitude (50.3 ± 22.5 µV, *n* = 10 eyes [6 and 4 male and female eyes, respectively]) compared to their pre-injury baseline (81.6 ± 24.3 µV, same cohort; Fig. 3C, D). Repeated-measures one-way ANOVA confirmed a significant effect over time (*F*(2.383, 21.45) = 3.977, *p* = 0.0281), with Tukey’s post hoc for multiple comparison analysis indicating that the reduction in amplitude occurred between 6- and 9-months post injury (*p* = 0.0345, Supplementary Data 1). In contrast, the implicit time of the P50 peak in the PERG response did not change significantly from 30.2 ± 4.0 ms at baseline in the naïve condition to 33.7 ± 2.8 ms at 9 months post-injury (Fig. 3E; repeated-measures one-way ANOVA, *F*(2.353, 21.18) = 2.613, *p* = 0.0894; Supplementary Data 1).

### Nine-month post-CHIMERA assessment reveals retinal vasculature remodeling

Comparative analysis of indocyanine green angiography (20 min after intraperitoneal injection) enabled visualization and analysis of the inner retina vasculature 9 months post-injury. In all animals assessed, the indocyanine green (ICG) remained confined to the blood vessels indicating that the closed head impact trauma did not leak out due to damage to the blood-retinal barrier (Fig. 3F). Quantification revealed a significant change in the maximal retinal vascular diameters measured compared to naïve animals (Fig. 3G; unpaired two-tailed t-test: *p* = 0.0442; *n* = 8 [4 male and female] retinas for naïve, and *n* = 10 [6 and 4 male and female, respectively] retinas at 9-month post-CHIMERA; Supplementary Data 1). In contrast, no significant differences were observed in retinal vascular tortuosity (Fig. 3H, unpaired two-tailed t-test: *p* = 0.6170; same cohort; Supplementary Data 1). Additionally, a significant reduction in the number of branches was detected at 9 months post-CHIMERA compared with naïve animals (Fig. 3I, unpaired two-tailed t-test, *p* = 0.0109; same cohort; Supplementary Data 1). We also investigated irregular areas of hypofluorescent or hyperfluorescent staining. By analyzing the pixel values of the major vessels in each image (Fig. 3J), we determined that the ICG vascular intensity was more variable in animals 9 months post-injury compared to the naïve animals (Fig. 3K). This resulted in a wider confidence interval for the average of the deviations (Fig. 3L) and was found to be statistically different by Levene’s Test for equality of variances (*p* = 0.001; same cohort; Supplementary Data 1).

### Histological analysis reveals regional retinal ganglion cell loss at 9 months post-injury

**Fig. 4:**
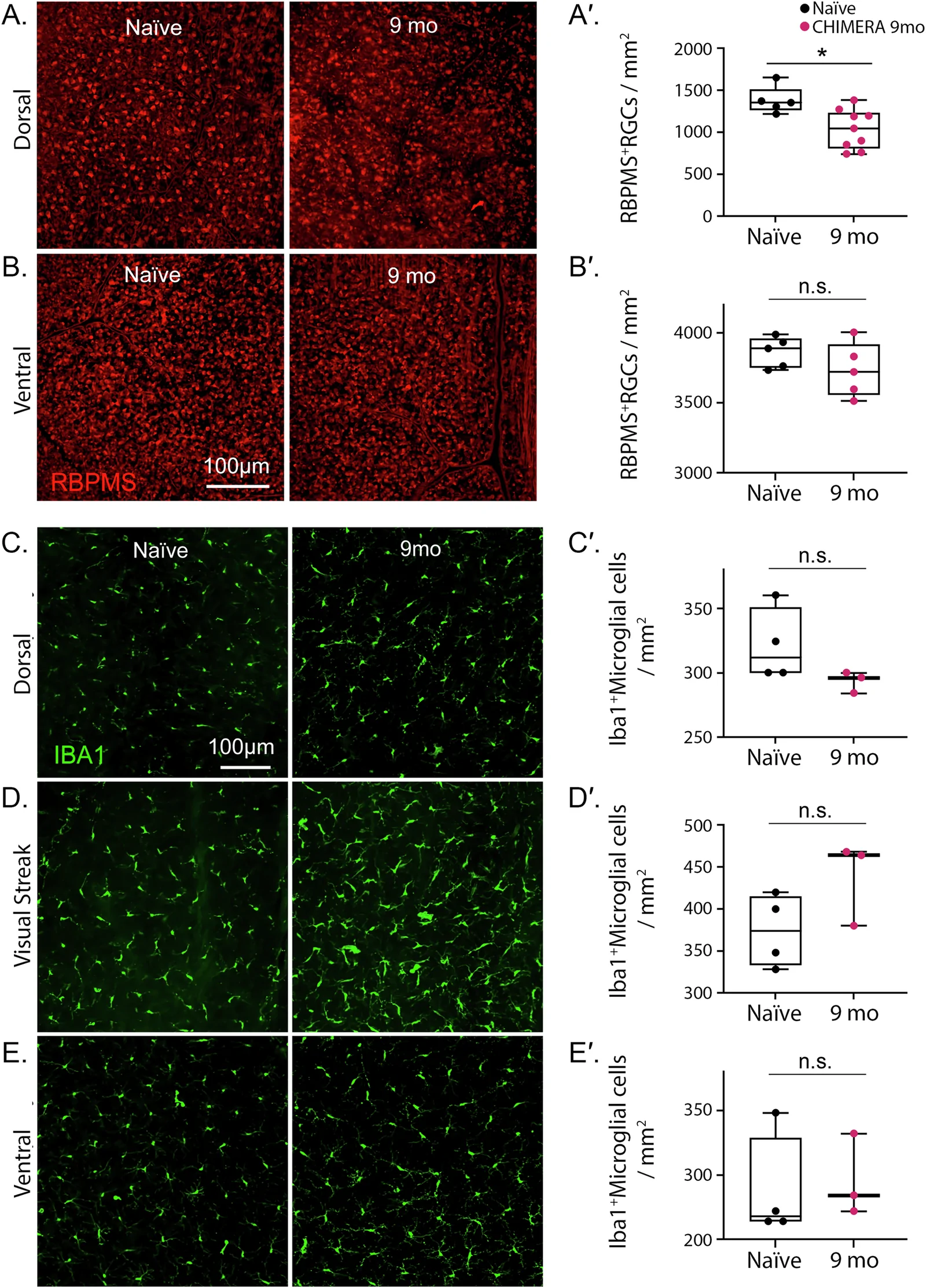
Regional differences in RGC and microglial density following CHIMERA injury. Immunofluorescence staining of retinas in thirteen-lined ground squirrels at 9-months post-injury. Confocal images of retinal flat mounts immunolabeled with RBPMS (red) to identify RGCs in the **A** dorsal periphery and **B** mid-ventral. **A′**, **B′** Box plots of RGC density in dorsal and ventral regions showing a significant loss of RGCs in the dorsal periphery at 9-months post-injury compared to uninjured controls. **A′** *n* = 5 [3 male and 2 female] and 9 [5 male and 4 female] eyes, for naïve and 9-month post-CHIMERA groups, respectively; unpaired two-tailed Student’s *t* test, *p* = 0.0138. **B′** *n* = 5 [3 male and 2 female] and *n* = 5 [4 male and 1 female] retinas for naïve retinas and at 9-months post-CHIMERA, respectively; unpaired two-tailed Student’s *t* test, *p* = 0.232. **C**, **D**, **E** Representative confocal images of IBA1-labeled microglia (green) in naïve and 9-month post-CHIMERA retinas from the **C** dorsal, **D** visual streak, and **H** ventral retina. **C′**, **D′**, **E′**: box plots of microglia density in dorsal, visual streak, and ventral retinas (*n* = 4 [1 male and 3 female] and 3 [3 male] eyes, for naïve and 9-month post-CHIMERA groups, respectively; unpaired two-tailed Student’s *t* test, n.s., not significant). RGCs and from each retina were automatically quantified within regions of interest (ROIs) measuring 0.64 × 0.64 mm, while microglia cells were manually marked and quantified within ROIs of 0.5 mm × 0.5 mm. Scale bars: 100 µm. See Supplementary Data 1 for statistical details. The whiskers on all boxplots shown **A’–E’** extend to the 90th (upper) and 10th (lower) percentiles.

At 9 months post-injury and in age-matched naïve animals, we concluded the study by performing histological analysis of the retinas and ONs. RGC density was quantified in both the dorsal and ventral retina along the periphery (Fig. 4A, B), as previous studies have reported that peripheral RGC loss is an early feature in TBI models^49,50^. Whole-mount retinas were stained for RBPMS (red), and labeled RGCs were quantified. A significant reduction in RGC density compared to naïve animals was observed in the dorsal retina (Fig. 4A′; unpaired two-tailed t-test, *p* = 0.0138; *n* = 5 images [3 male and 2 female] from naïve retinas; and *n* = 9 images [5 male and 4 female retinas] at 9-months post-CHIMERA, respectively; Supplementary Data 1), but not in the ventral retina (Fig. 4B′; unpaired two-tailed t-test, *p* = 0.232; *n* = 5 images [3 male and 2 female] for naïve retinas, and *n* = 5 images [4 male and 1 female] at 9-months post-CHIMERA; Supplementary Data 1). We also examined regional differences in microglial density across the retina (dorsal periphery, visual streak, and ventral periphery) at 9 months post-injury. Interestingly, although the differences did not reach statistical significance, the dorsal periphery (Fig. 4C-C′; unpaired two-tailed t-test, *p* = 0.1697; *n* = 4 images [1 male and 3 female] for naïve retinas, and *n* = 3 images [3 male] for retinas at 9-months post-CHIMERA; Supplementary Data 1) showed a trend toward fewer microglial cells per mm² in the 9-month injury group compared to controls (Fig. 4C′). In contrast, the visual streak tended to show a higher number of microglia (Fig. 4D) in the 9-month injury group, although this difference also did not reach statistical significance (Fig. 4D′; unpaired two-tailed t-test, *p* = 0.1305; same cohort; Supplementary Data 1). Although this increase is not significant, it could reflect RGC functional alterations as confirmed by the visual acuity test of OMR (Fig. 3B) and PERG (Fig. 3C, D). It is possible that some degeneration occurred within the visual streak but was not readily apparent due to the multilayered organization of RGCs in this region, variability among animals, or the reduced sample size analyzed (*n* = 4 and 3 eyes, naïve and 9-month post-CHIMERA, respectively). In the ventral periphery (Fig. 4E), we observed no difference in microglial number (Fig. 4E′, unpaired two-tailed t-test, *p* = 0.7659; same cohort; Supplementary Data 1), consistent with the absence of RGC loss in this area.

### Optic nerve analysis reveals microglial activation 9 months post-injury

**Fig. 5:**
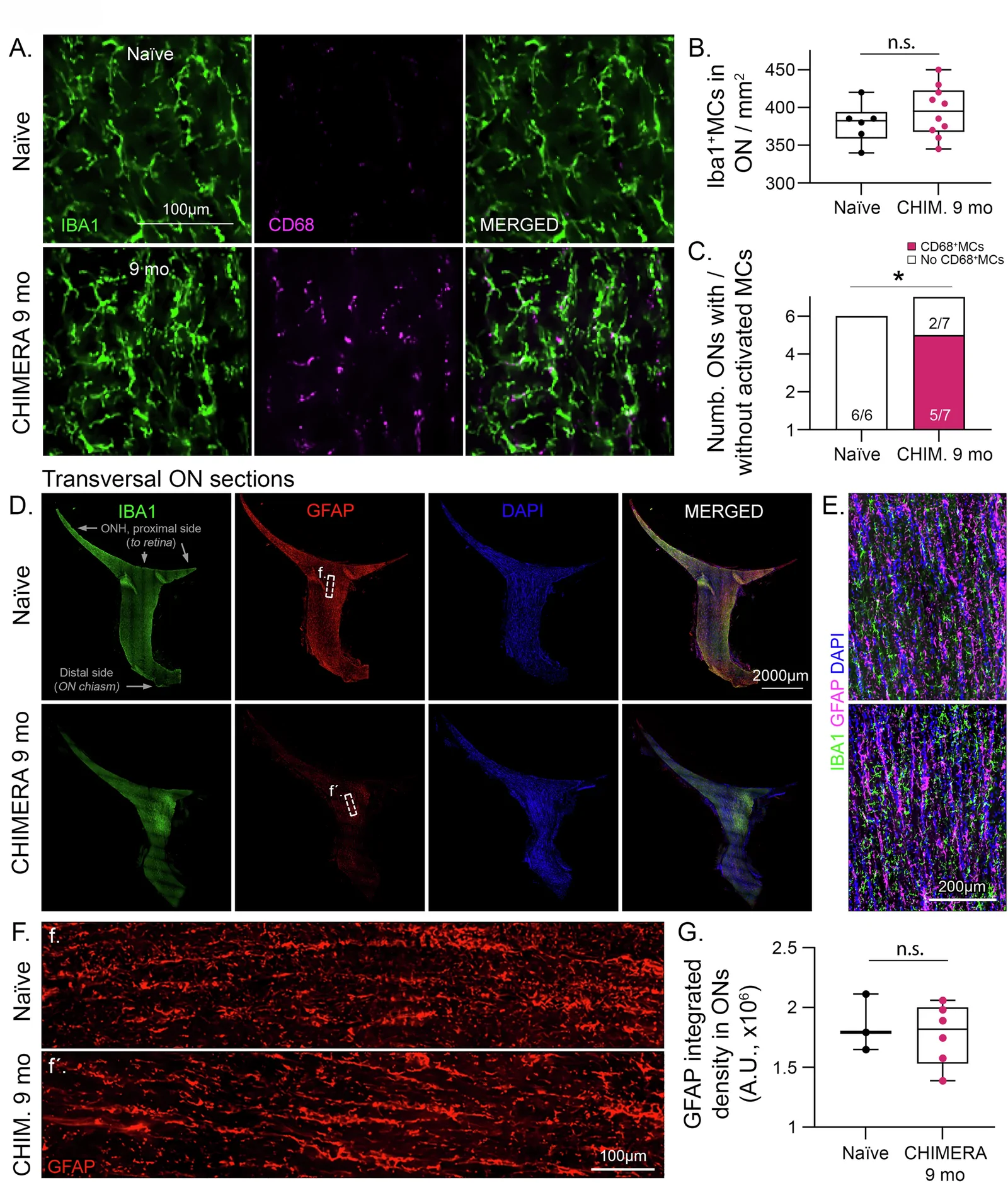
Confocal images of longitudinal optic nerve (ON) sections from naïve thirteen-lined ground squirrels and animals 9 months post-CHIMERA injury. **A** Representative cropped regions from a naïve ON and at 9-months post-CHIMERA showing microglia labeled with IBA1 (green) and CD68 (magenta). **B** Manual quantification of microglial density (cells per 1 mm × 0.2 mm area) in naïve controls (black, *n* = 6 [3 male and female]) and injured animals (magenta, *n* = 10 [4 male and 6 female]). Unpaired two-tailed Student’s *t* test, n.s., not significant. **C** Bars represent the proportion of ONs exhibiting microglial activation in each group; individual ONs are categorized as positive or negative for Iba1/CD68 co-localization. A significantly higher proportion of CHIMERA ONs showed activation compared with controls (5/7 [6 ONs from male and 1 from female animals] vs. 0/6 [3 ONs from male and female animals]; two-sided Fisher’s exact test, *p* = 0.0210). **D** Longitudinal ON sections immunostained for IBA1 (green, microglia/macrophages) and GFAP (red, astrocytes), with DAPI counterstain (blue, nuclei), in naïve (top row) and 9-month post-injury animals (bottom row), depicting proximal (to retina) and distal (to optic nerve chiasm) portions of the ONs. Scale bar = 2000 µm. **E** Representative higher magnification views of microglia and astrocytes counterstained with DAPI for naïve (top row) and 9-month post-injury animals (bottom row). Scale bar: 200 µm. **F** Higher magnification images of regions indicated by dashed boxes in (**D**) (**f-f′**), illustrating the GFAP expression. Scale bar: 100 µm. **G** Box plot of integrated GFAP fluorescence density. Statistical comparisons were performed using an unpaired two-tailed Student’s *t* test; n.s. not significant (*n* = 3 [2 male and 1 female] and 6 [4 male and 2 female] ONs, naïve and 9-months post-CHIMERA groups, respectively). See Supplementary Data 1 for statistical details. The whiskers on all boxplots shown (**B**, **G**) extend to the 90th (upper) and 10th (lower) percentiles.

Examination of ONs from age-matched naïve and 9-month post-injury animals (Fig. 5A) revealed that microglia cell density evaluated by IBA1 expression in injured animals appeared comparable to controls (Fig. 5B, unpaired two-tailed t-test, *p* = 0.0546; *n* = 6 [3 male and female] and 10 [4 male and 6 female] ONs for naïve and at 9-months post-CHIMERA, respectively; Supplementary Data 1). However, evidence of significantly higher microglial activation was observed in most TBI samples (Fig. 5C), as indicated by regions of more prominent CD68 staining within portions of the ON (Fig. 5A). If this activation occurs only in specific regions, it could explain why not all sections exhibited clear differences—given that the ON of the thirteen-lined ground squirrel is relatively thick and only a subset of longitudinal sections was stained, potentially missing the affected branch. Nonetheless, IBA1 and CD68 co-localization indicate that 71% of ONs subjected to CHIMERA exhibit microglial activation, whereas none of the control ONs showed an increased CD68 signal (5/7 [6 male and 1 female] vs. 0/6 [3 male and female] ONs in CHIMERA and control groups, respectively; two-sided Fisher´s exact test, *p* = 0.0210; Supplementary Data 1). In contrast, glial fibrillary acidic protein (GFAP) expression did not differ significantly between groups (Fig. 5D, E) as quantified in comparable regions of interest adjacent to the ONH (Fig. 5F, G**;** unpaired two-tailed Student’s *t* test, *p* = 0.6757; *n* = 3 [2 male and 1 female] and 6 [4 male and 2 female] ONs for naïve and 9-months post-CHIMERA, respectively; Supplementary Data 1).

## Discussion

Our findings establish the thirteen-lined ground squirrel as a relevant model for studying ocular and behavioral changes associated with traumatic brain injury. After determining that the confound of torpor was reliably removed, we used a repeated CHIMERA impact paradigm with an interface between the animal’s head and the piston to generate biomechanical forces that induced measurable structural, functional, and behavioral alterations without causing overt skull damage. TBI-exposed thirteen-lined ground squirrels exhibited persistent deficits in RGC function, as evidenced by reduced pattern ERG amplitudes, consistent with findings from other studies of blast-induced injury^51,52^. These functional deficits were accompanied by transient reductions in visual acuity measured by optomotor responses; however, ERG recordings showed no deficits in photoreceptor and bipolar cell response amplitude, but is associated with a subtle, time-dependent slowing of phototransduction kinetics, which may reflect mild alterations in photoreceptor response dynamics and/or early changes in post-receptor signal transmission without overt synaptic or cellular loss. Together, these findings suggest that the visual impairments observed in thirteen-lined ground squirrels at 9 months post-CHIMERA are primarily attributable to damage within RGCs and the ON pathway, rather than to dysfunction of other retinal neuronal populations.

In agreement with these functional and behavioral results, in vivo anatomical evaluation using OCT revealed a significant thinning of the total retinal thickness at 4.5 months post-injury and a subsequent increase in thickness at 9 months, possibly reflecting dynamic structural remodeling processes rather than sustained degeneration, potentially involving glial responses, such as Müller cell or astrocyte reactivity, as well as inflammation-associated tissue changes that can temporarily influence retinal thickness. RGC degeneration was supported by PERG measurements at 9 months post-injury, which showed reduced amplitudes indicative of persistent RGC dysfunction. Ex vivo confocal imaging of retinal flat mounts revealed peripheral RGC loss, confirming these observations. However, although we could not reliably detect significant RGC degeneration within the visual streak—likely due to the high density of RGCs in this region^14,19^, which may obscure subtle RGC loss—we observed a trend toward increased microglial cell density in the visual streak. This likely reflects the fact that microglia are primarily recruited to sites of local axonal degeneration and debris clearance. Following the initial injury and subsequent wave of neurodegeneration, the inflammatory response diminishes in regions where RGCs have already been lost, leading to a reduced microglial presence over time. These events were accompanied with subtle alterations in retinal vascular diameter and branching, and changes in microglial markers in the ON, as reported previously^53^, which are consistent with chronic post-injury pathology. In this context, the increased prevalence of localized microglial activation (Iba1/CD68 co-localization) in ONs following CHIMERA is likely associated with ongoing focal axonal degeneration. Future studies using optical clearing and whole-nerve imaging^54^ could determine whether microglial activation is regionally concentrated or more widespread^55^. These results are consistent with previous reports describing axonal varicosities, retraction bulbs, and axon loss in the optic tract and ON of mice and rats subjected to TBI^56–59^, suggesting that such alterations may contribute to chronic pathology^60^. However, it is important to note that axonal abnormalities can, in some cases, resolve within days to months after injury^60,61^, highlighting the heterogeneity of TBI outcomes. In addition, although previous studies have reported increased GFAP immunoreactivity following TBI^62–64^, we did not observe differences in GFAP levels in ON astrocytes at 9 months post-injury, which may reflect a resolution of astrocytic reactivity at chronic time points, highlighting the dynamic nature of glial remodeling following TBI.

Notably, whereas multiple CHIMERA impacts typically result in more severe degeneration in nonhibernating species, such as mice and rats, the thirteen-lined ground squirrel demonstrated relative resilience to 3 consecutive days of 2 CHIMERA impacts, suggesting that a greater number of consecutive injury days (e.g., 5 days) may be required to elicit more robust pathological changes. TBI-induced systemic consequences, including disruptions in body weight regulation and alterations in visual function, may arise from combined retinal damage and central nervous system injury^65–67^. Alternatively, damage to the white matter tracts may cause demyelination or impair axonal transport, disrupting signal transmission, altering both behavior and visual outcomes^6,8,68^. Collectively, these results demonstrate that the thirteen-lined ground squirrel recapitulates key features of human impact-related TBI—including visual system vulnerability, metabolic dysregulation, and behavioral deficits—providing a novel and translationally relevant platform for investigating mechanisms and potential interventions for traumatic brain injury. Interestingly, three of the five CHIMERA-injured animals exhibited abnormally elevated body weight earlier than expected. Because the spleen is of particular interest in TBI—undergoing dynamic changes in size that reflect activation and mobilization of immune cells^45^ and forming part of the “brain-spleen axis,” a bidirectional communication pathway between the nervous and immune systems after brain injury^69–72^—we evaluated spleen and kidney weights. However, no significant differences were observed between CHIMERA-injured and naïve animals, which may reflect either higher resilience or a more localized systemic response to TBI in thirteen-lined ground squirrels compared with other small rodents.

The observed changes in the retinal vasculature suggest its potential as a sensitive biomarker for TBI and a target for therapeutic intervention. In CHIMERA-injured animals, the nonuniform binding and filling of the inner retinal vasculature with ICG dye may reflect disrupted blood flow (ischemia), damage to the vessel walls, or mechanical disruption. Additionally, in vivo assessment of apoptotic RGCs or reactive immune cells using Annexin-V can serve as a biomarker of degenerative events in the retina, potentially enabling earlier therapeutic intervention^73,74^.

Because the CHIMERA model is based on traumatic head impact with accelerated rotational movement, consistent with prior studies, we focused our analyses on the primary visual pathway, the RGCs and their axons, which represent the most vulnerable retinal population under these biomechanical conditions. Given the unique horizontal orientation of the ON head of the thirteen-lined ground squirrel, it is plausible that mechanical forces acting on the nerve contribute to axonal or vascular injury sufficient to produce regionally selective RGC loss. In this study, we evaluated RGC loss in dorsal and mid-peripheral ventral retinas because these regions contain fewer RGCs, which are characterized by larger and more sparsely distributed somas that are organized primarily in a monolayer. In contrast, the central retina exhibits a markedly higher RGC density, with an exceptional multilayered arrangement along the visual streak^14,15,19^. This higher baseline density complicates detection of modest RGC loss in retinal flat mounts using RBPMS, as overlapping cytoplasmic labeling in two-dimensional imaging limits accurate cell discrimination. Consequently, this approach is more sensitive to relatively pronounced degeneration within the visual streak^75^. Nevertheless, the observed reduction in visual acuity measured by OMR strongly suggests that the visual streak is functionally affected. Thus, both vascular and topographic considerations would be critical when evaluating localized injury effects in this model.

We did not evaluate other retinal neuronal populations or perform retinal analyses using cryosections. This decision was based on our primary objective to assess degeneration within the primary visual pathway, where traumatic brain injury is expected to preferentially affect RGCs as a consequence of axonal damage to the ONs. Consistent with this rationale, previous studies have shown that ON axotomy does not result in significant loss of non-RGC neurons within the ganglion cell layer even after prolonged periods up to 15 months^76^, nor does it lead to photoreceptor loss up to 6 months post-injury^77^. Consistent with that, our ERG recordings demonstrate no deficits in photoreceptor and bipolar cell function, and therefore, the visual impairments observed are mostly due to damage to RGCs and their axons. Nevertheless, we acknowledge that secondary or indirect retinal changes—potentially mediated by glial responses, such as microglial or Müller cell activation^78^—cannot be fully excluded and warrant further investigation in future studies.

One limitation of this study is that we cannot definitively claim to have removed all potential hibernation-related influences, and thus, there may be some metabolic impacts of hibernation on the animals in this study. However, hibernation is not a stable physiological state but a highly dynamic process. During the hibernation season, animals undergo prolonged periods of deep torpor lasting 10–14 days, interspersed with short interbout arousals (IBA) of approximately 8–12 h, during which body temperature transiently returns to ~37 °C before the animals re-enter deep torpor. Importantly, animals in IBA are still considered part of the hibernation cycle and are expected to return to torpor shortly thereafter. In our paradigm, however, animals never entered a true hibernation state: they exhibited neither deep torpor nor IBA. Although these animals initially made brief, unsuccessful attempts to hibernate, these attempts ceased, and the animals remained physiologically awake throughout the winter. Consequently, we consider the animals in this study to be fundamentally different from hibernating animals sampled during IBA. Continuous body temperature monitoring confirmed stable euthermia with only normal circadian fluctuations, further supporting the conclusion that these animals were not engaged in the hibernation program. While a more comprehensive molecular characterization would be required to definitively demonstrate complete equivalence to summer-euthermic physiology, studies have shown that even animals sampled during IBA exhibit physiological parameters comparable to awake conditions^79^. In addition, throughout the experimental period, animals were maintained under seasonally adjusted light-dark cycles and provided ad libitum access to food and water, as previously described^80^, but were deliberately not placed in a dark, cold hibernaculum. These housing and husbandry conditions were specifically implemented to prevent the induction of a hibernation cycle. Thus, taken together, these observations support our interpretation that thirteen-lined ground squirrels that did not hibernate during winter are more representative of normal awake conditions than of hibernating states, and that our results are unlikely to be confounded by hibernation-related physiology.

Due to the regulatory status of the thirteen-lined ground squirrel under the Animal Welfare Act (AWA), the experimental design required careful optimization to minimize animal numbers and experimental attempts while still allowing determination of whether TBI induces measurable alterations in the visual pathway. The AWA, enforced by the United States Department of Agriculture (USDA), establishes standards for the care, treatment, and transportation of covered animals to ensure humane treatment and welfare. As a U.S. federal law, the AWA applies specifically to animal research conducted within the United States and does not extend to other countries. In contrast, the use of this species in other countries would be governed by their respective regulatory and ethical frameworks.

Within this context, in our study, this designation influenced experimental design through Institutional Animal Care and Use Committee (IACUC) oversight, which required careful justification and minimization of animal use. This was particularly important given that this injury model had not previously been established in the thirteen-lined ground squirrel, necessitating a cautious, pilot-scale approach. Accordingly, this study was designed to generate foundational data while limiting animal use.

As described in the methods, the animals used in this study can be sourced from an established research colony at the University of Wisconsin–Oshkosh. Previous work by Dr. Dana Merriman and colleagues has outlined effective strategies for successful captive breeding and colony maintenance of this species^80^. However, compared to traditional rodents, the thirteen-lined ground squirrel requires specialized husbandry, including seasonally adjusted photoperiods and access to specialized low-temperature environments (hibernacula) to support torpor and hibernation during winter. These requirements add logistical complexity and may limit broader adoption.

As with other AWA-covered species (which include most warm-blooded animals except birds, rats, and mice bred for research), research protocols involving thirteen-lined ground squirrels are subject to heightened scrutiny and more extensive justification. This pilot study provides foundational parameters for generating closed-head impact TBI in this model and is intended to guide future research while facilitating more efficient ACUC review and approval.

As the field continues to move toward new approach methodologies aimed at improving human relevance, the thirteen-lined ground squirrel represents a valuable niche model, particularly for studies of neuroprotection and metabolic suppression. However, its broader and long-term utility will depend on the continued development of sustainable captive breeding programs, standardized husbandry practices, and clear regulatory pathways that balance scientific use with animal welfare.

Because thirteen-lined ground squirrels retain largely intact visual function despite injury, this model is particularly well-suited for longitudinal behavioral studies—including motor, cognitive, and social assessments—without the confounding effects of severe sensory deficits. Furthermore, transcriptomic analyses following acute and chronic head injury in hibernating animals subjected to CHIMERA may uncover hibernator-specific neuroprotective mechanisms that preserve neural and visual function^81,82^. Collectively, these results position the thirteen-lined ground squirrel as a unique and highly versatile model for investigating the mechanisms, progression, and treatment of traumatic brain injury, bridging the gap between preclinical studies and human TBI, and may accelerate the development of effective interventions.

## Methods

### Ethics approval

All animal procedures were conducted in accordance with U.S. federal laws and Department of Agriculture regulations governing the humane use of animals in research and in adherence to the ARVO Statement for the Use of Animals in Ophthalmic and Vision Research (see https://www.arvo.org/About/policies/arvo-statement-for-the-use-of-animals-in-ophthalmic-and-vision-research/). Experimental protocols were reviewed and approved by the National Eye Institute Animal Care and Use Committee (NEI ASP-699 and NEI ASP-595). We have complied with all relevant ethical regulations for animal use.

### Animals

Adult male and female thirteen-lined ground squirrels (*Ictidomys tridecemlineatus;* 6–15-month-old) were initially obtained from Dr. Dana Merriman (University of Wisconsin–Oshkosh) and transported to the NIH animal facility. Upon arrival, animals were clinically evaluated by veterinary staff and placed under a quarantine period, after which a second veterinary assessment confirmed health and suitability for experimentation. Animals were then implanted with subcutaneous temperature transponders for continuous monitoring of core body temperature and housed individually under standard laboratory conditions with seasonally adjusted light-dark cycles designed to replicate natural photoperiods in Wisconsin (Fig. 1B). Briefly, environmental temperature (gray bars) was gradually increased from 60 ± 4 °F in December to 72 ± 4 °F by late spring–summer, with incremental changes of ~1 °F each 2-weeks period. In parallel, the photoperiod (orange trace) was extended from 8.5 to 9 h of light in December–January to approximately 15 h in June, increasing by ~1.5 h per month. Food and water were provided ad libitum, with seasonal dietary enrichment supplied in accordance with approved standard operating procedures (SOPs) as previously reported^80^. Briefly, high-protein Cat Chow was provided ad libitum, together with weekly dietary enrichment consisting of a teaspoon of Jumble (trail mix containing sunflower seeds), a teaspoon of sunflower seeds, a teaspoon of Monkey Jumble™ mix, two peanuts, and 1/8 section of fruit (apple, orange, or a grape). Animals are housed individually in cages (9.5 × 13.5 × 8.5 inches) with 1 inch of Aspen Sani-Chip® bedding. Environmental enrichment, consisting of a red plastic cylinder (Bio-Serv Rat Tunnel) and a wooden cube, was provided throughout the study to promote exploratory behavior and animal welfare. Throughout the study period, thirteen-lined ground squirrels were maintained under these conditions and monitored twice a week by trained personnel to assess general health and well-being.

The following animals were used in this study:

#### Experimental groups

*The CHIMERA-injured group consisted of five squirrels (magenta*, *Fig*. *1C**):* *1799 (female), DOB: 5-20-24; 1803 (female), DOB: 5-21-24; 1812 (male), DOB: 5-22-24;* *1813 (male), DOB: 5-22-24; 1814 (male), DOB: 5-22-24*

*The naïve (uninjured) control group consisted of four squirrels (gray*, *Fig*. *1C**):* *1846 (female), DOB: 6-6-24; 1827 (male), DOB: 5-23-24; 1779 (male), DOB: 5-20-24;* *1833 (female), DOB: 5-23-24*

To control potential differences in body weight associated with hibernation, a subset of thirteen-lined ground squirrels (*n* = 6) was allowed to enter hibernation in the fall after exhibiting signs of spontaneous torpor. These animals were transferred by veterinary personnel from standard housing conditions (ambient room temperature) to a dedicated hibernaculum maintained at 4 °C, as reflected in Fig. 1C. Following transfer, animals were monitored daily, and after three consecutive days in stable torpor (confirmed by a body temperature of ~4 °C) they were placed in individual containers with appropriate bedding, without access to food or water, consistent with standard hibernation protocols. Health status was assessed twice weekly, and body weight was recorded periodically throughout the hibernation period. Importantly, these animals were used exclusively for body weight monitoring and were not subjected to any experimental procedures or included in subsequent analyses.

*The hibernating naïve (uninjured) control group consisted of six squirrels*(*cyan*, Fig. 1C)*:*

*1776 (male), DOB: 5-20-24*

*1817 (male), DOB: 5-20-24*

*1824 (male), DOB: 5-20-24*

*1845 (female), DOB: 6-4-24*

*1790 (female), DOB: 5-20-24*

*1804 (female), DOB: 5-21-24*

### Study design

Five nonhibernating thirteen-lined ground squirrels were subjected to CHIMERA (Fig. 1A) in early December (mixed sex; 3 males and 2 females, ~6.5 months of age at the time of injury), yielding *n* = 10 eyes and ONs. An additional four nonhibernating age-matched animals served as naïve controls (mixed sex; 2 males and 2 females, ~6.5 months of age), yielding *n* = 8 eyes and ONs. Inclusion criteria: all animals selected were adults within their first year of age that were apparently healthy as assessed by season-appropriate body weight according to institutional SOP (with a minimum body weight threshold of 170 g), active and alert behavior, normal coat coloration, and absence of hair loss or other visible signs of illness. In addition, animals were selected to achieve a balanced representation of sexes, with approximately equal numbers of male and female animals included in the study.

To evaluate visual impairment and degeneration induced by CHIMERA in thirteen-lined ground squirrels under awake conditions (thereby avoiding potential confounding neuroprotective effects of hibernation) and because degenerative changes following traumatic brain injury may be more readily detectable at later stages, we extended the anatomical evaluations to 9 months post-CHIMERA and ensured that thirteen-lined ground squirrels remained predominantly in euthermic conditions throughout the post-injury period.

Thirteen-lined ground squirrels are obligate hibernators who start hibernating in the fall through the winter, with many documented molecular, genetic, and anatomical changes during hibernation^83–87^. During winter, animals often initially undergo a brief transitional period during which they attempt to enter hibernation, a phase characterized by a temporary decrease in body temperature, which reflects the expected metabolic shift associated with the onset of torpor. However, if the animals are not provided with the environmental conditions required for deep torpor (cold ambient temperatures), torpor occurs transiently. Following this period, body temperature stabilizes and subsequently fluctuates only within a normal circadian range, typically showing minor day-night variations of a few degrees, which are not comparable to the pronounced hypothermia observed during torpor. Consequently, in the 9-month period of our experiments, all animals in the study were kept in euthermic conditions (ambient temperature, food, and water) to prevent confounding torpor-related readouts. In fact, it has been described that animals collected at the interbout arousal (IBA), a transitory phase of hibernation where animals wake up, and their body temperature increases to euthermic values, resemble thirteen-lined ground squirrels more in active conditions than those in deep torpor^79^. Body temperature in the selected animals was continuously monitored in real time using implanted transponders to confirm that animals were no longer attempting to hibernate and returned to a stable euthermic state. Thus, we objectively verified that these animals were not in a prolonged pre-hibernation or metabolically compromised condition. In addition, animals that did not undergo a hibernation cycle were evaluated twice per week throughout the study, and no health issues were observed; to date, we are not aware of any evidence indicating that missing a single hibernation cycle adversely affects health in thirteen-lined ground squirrels.

All animals subjected to this study underwent noninvasive anatomical (OCT), functional (ERG, PERG), and behavioral assessments (OMR). In some experiments (ERG), additional naïve animals were included at a later time point (9 months; *n* = 6 animals, mixed sex; 3 males and 3 females, at ~15 months of age, yielding *n* = 12 eyes) to minimize the influence of age on baseline measurements. At the conclusion of the 9-month study, the retinas and ONs were collected from the four naïve animals and the five thirteen-lined ground squirrels subjected to repeated CHIMERA injury (Fig. 1B).

#### Implantation of subcutaneous temperature transponders

Thirteen-lined ground squirrels were anesthetized using inhaled isoflurane delivered in 100% oxygen (2–4%) within an induction chamber for approximately 4–6 min. Once anesthetized, animals were transferred to a heated surgical platform and maintained under 3% isoflurane in dorsal recumbency throughout the procedure. Physiological parameters, including respiration, heart rate, and body temperature, were continuously monitored during surgery. The surgical area was shaved and aseptically prepared with alternating applications of disinfectant soap and isopropyl alcohol for three consecutive cycles. Topical proparacaine was applied at the incision site to provide local anesthesia.

Wireless temperature bio-loggers (DST micro-ACT; 2.5 × 0.8 cm; Star-Oddi, Garðabær, Iceland) were implanted subcutaneously in the region posterior to the scapulae. A small incision was made in the subscapular area, and a subcutaneous pocket was carefully formed to position the transmitter approximately 1–2 cm from the incision site, minimizing tension on the sutures and reducing the likelihood of wound dehiscence. Skin closure was performed using 5-0 non-absorbable sutures. Implanted bio-loggers recorded body temperature at 15-min intervals, and data were collected through telemetry. Animals received postoperative analgesia with ketoprofen (5 mg/kg).

### CHIMERA induction

Thirteen-lined ground squirrels (*n* = 5) were deeply anesthetized prior to CHIMERA exposure. Each animal was individually placed in an induction chamber and anesthetized with 2–4% isoflurane in 100% oxygen for 4–6 min. Following induction, animals were quickly transferred to the CHIMERA platform (located in the same room) and secured in the supine position using Velcro straps.

According to USUHS and ACUC protocol, Acetaminophen (Tylenol®, 1 mg/mL; approximately 100–200 mg/kg body weight) was provided in the drinking water 24 h before injury and maintained until 24 h post-injury, consistent with published dosing^88^ and prior experience with rats in this model.

CHIMERA procedures followed standard techniques^58^. The animal’s head was positioned so that the piston contacted the scalp just anterior to the bregma suture, delivering an impact of ~1.5 J. A nose cone continued to deliver 2% isoflurane during positioning but was removed immediately prior to impact. Designated thirteen-lined ground squirrels received two consecutive impacts separated by 60–90 s, during which animals were briefly returned to isoflurane anesthesia while they were repositioned. The CHIMERA device uses compressed air to drive a metal piston, with a solenoid valve controlling the release to achieve precise impact energy^89^, and is designed to generate reproducible TBI of varying severities using controlled, quantifiable biomechanical inputs in a nonsurgical platform^90^. Velocity sensors verify piston speed, ensuring reproducible biomechanical forces. Following impact, animals were placed in a clean nesting cage on a warming pad (maintained by circulating warm water at approximately 37 °C) and monitored until fully conscious and demonstrating normal mobility before returning to their home cage. Naïve thirteen-lined ground squirrels (*n* = 4) were placed in a similar position and received a similar duration of isoflurane exposure as the CHIMERA cohort; however, this exposure for the naïve animals occurred at the NIH rather than at the USUHS, and therefore, transport of the naïve animals to USUHS was not required. Only the CHIMERA animals were transported to and from USUHS on the same day (round trip). Animals were only housed at the NIH facility. Naïve thirteen-lined ground squirrels did not receive acetaminophen in the drinking water. Both anesthesia and CHIMERA procedures were brief, lasting approximately 3–5 min in total. Consequently, body temperature and body weight did not fluctuate substantially during the procedure, making it highly unlikely that the intervention induced any hibernation-like physiological responses in these animals. Additionally, animals were maintained at ambient room temperature, with ad libitum access to a normal food regimen and water.

To establish the CHIMERA model in thirteen-lined ground squirrels, pilot experiments were performed using one animal at a time to assess feasibility, safety, and the induction of measurable pathology. These pilot animals were used exclusively for model optimization, were not included in the final experimental cohorts, and were not reused for subsequent experiments. Single impacts at varying energies (0.5, 1, and 1.5 J, *n* = 1 per group [3 females]), as well as consecutive impacts delivered within 30 s on the same day, were tested. None of these conditions produced detectable pathological changes in the visual pathway, probably due to the CHIMERA model, the injury location, the animal model, and/or the animal anatomy, such as the skull thickness, despite multiple publications using mice that report damage from one impact^90–94^.

Throughout model development, animals were closely monitored for unexpected adverse effects, including failure to recover after impact. In such cases, predefined humane endpoints approved by the institutional protocol would have been followed (euthanasia using isoflurane overdose, achieved by administration of 5% isoflurane in 100% oxygen in an induction chamber until respiratory arrest, followed by confirmation of death and cervical dislocation). Throughout the course of this study, no animals exhibited conditions requiring application of the predefined humane endpoints.

### Optical coherence tomography (OCT)

OCT imaging was performed in both eyes (*n* = 10 eyes) from the same animals at three time points: baseline (pre-injury), and 4.5-, and 9-months post-CHIMERA (Fig. 2A), using spectral domain-optical coherence tomography (SD-OCT, Heidelberg Engineering, Heidelberg, Germany). Animals were anesthetized with 5% isoflurane for induction and maintained at 3–4.5% under a bRetinal nerve fiber layer (RNFL) thickness—representing the unmyelinated axons of RGCs—and total retinal thickness were measured in the region of the visual streak, located approximately 2 mm inferior to the ON head^14^. The retinal vasculature and horizontal axis of the ON were used as landmarks to standardize positioning across time points.

OCT acquisition settings were as follows: Fast preset, averaging 9 frames (ART 9), scan angle 55° × 20° (1536 A-scans), with 9 sections spaced 720 µm apart. The same animals were imaged at baseline, 4.5 months, and 9 months post-injury. Retinal thickness measurements (RNFL and total retinal thickness) were obtained using Heyex software (Heidelberg Engineering, Spectralis system) by applying the scale bar measurement tool to the corresponding retinal layers.

### Light-adapted electroretinogram (ERG)

Animals were induced with 5% isoflurane and maintained under anesthesia at 3–4.5% via a nose cone. After mydriasis induction (tropicamide), electrodes were placed on the corneas using Systane® eye drops for lubrication and electrical contact. A reference electrode was positioned in the hind limb, and an additional reference electrode was placed in the animal’s mouth. ERGs were recorded using the Diagnosys system (Diagnosys LLC, Lowell, MA, USA), and electrode impedances were verified to be below 5 kΩ before starting measurements. Xenon light flashes from 0.01 to 3500 cd s/m^2^ were used as stimulus. The background light in the ColorDome was set to 25 cd/m^2^. The flicker stimuli were 10 cd s/m^2^ at 5, 15, and 30 Hz over a background of 25 cd/m^2^ ^17^. All analysis was performed using custom software written in MATLAB (Mathworks). ERG recordings were obtained from both eyes of the same animals (*n* = 10 eyes) at two time points following CHIMERA induction: 4.5- and 9-months post-CHIMERA. In addition, two groups of age-matched naïve animals were examined as controls: one corresponding to the age of animals at 4.5 months post-CHIMERA (*n* = 8 eyes) and another matching the age of animals at 9 months post-CHIMERA (*n* = 12 eyes). Exact sample sizes are provided in the figure legends.

### Optomotor reflex (OMR)

Visual acuity assessment was performed using the OptoDrum system (Striatech GmbH, Tübingen, Germany). As we previously adapted this test for the thirteen-lined ground squirrel^95^, each animal was placed on an elevated platform at the center of an arena surrounded by four LCD monitors (Fig. 3A), which displayed a rotating black-and-white grating pattern at varying contrast levels. The gratings rotated clockwise or counterclockwise at 12°/s, and an automated tracking system recorded head movements in response to the stimulus.

Each stimulus was presented for a minimum of 1 s and terminated after a maximum of 5 s or if the animal became restless. A trial was considered successful if the head movement score exceeded the chance-level threshold for stimulus-independent movement. Trials alternated between clockwise and counterclockwise rotations until a valid response was recorded. Testing began at a spatial frequency of 0.5 cycles/°, with visual acuity defined as the highest spatial frequency at which the animal successfully responded in at least two independent trials. Testing was terminated once the animal failed three consecutive trials at the same spatial frequency. OMR measurements were obtained from naïve animals (*n* = 4) and from CHIMERA-injured animals re-evaluated at 3-, 6-, and 9-months post-injury (*n* = 5 animals). Additionally, the same naïve animals were re-evaluated 9 months later to match the age of the final post-CHIMERA assessment, thereby excluding aging as a contributing factor to changes in visual acuity.

### Pattern ERG

Animals were initially anesthetized with 5% isoflurane and maintained under anesthesia at 3–4.5% via a nose cone. Once anesthetized, animals were positioned in the large animal holder of the Jörvec pattern ERG system (Miami, FL). The stimulation protocol was as follows: stimulus: pattern, side: both, mode: Ipsi, pattern: 4, brightness: 15, modulation: PERG, sweeps: 0/4/31/372, rate: 4.05/s, time: 500 µs. Data analysis was performed using the Jörvec software, with peak amplitudes determined using the folding function and the implicit time measured at the P50 component. PERG measurements were obtained from both eyes (*n* = 10 eyes) of the same animals (*n* = 5) at baseline (pre-injury) and 3-, 6-, and 9-month post-CHIMERA.

### Indocyanine green angiography (ICGA)

Animals were induced using 5% isoflurane and maintained under anesthesia at 3-4.5% under a nose cone. Animals received intraperitoneal (i.p.) injections of indocyanine green (ICG, Diagnostic Green®) at a concentration of 5 mg ICG dye/mL of sterile water, and eyes were dilated with tropicamide and phenylephrine. Eyes were lubricated with Systane eye drops periodically to prevent desiccation. Images of vasculature were taken, 20 min after i.p. injection, using the ICGA setting on the imaging platform (Heidelberg Spectralis® HRA + OCT), and the final ICGA images were averaged from 100 scans.

### Angiography analysis

All retinal vasculature images were analyzed in Fiji (ImageJ). Visible vessels were manually traced and quantified using the simple neurite tracer (SNT) and Skeletonize plugins, and retinal vascular branches were quantified. Retinal vasculature tortuosity was calculated using the tortuosity index (TI), defined as the ratio of the path length along the vessel centerline (branch length) to the straight-line (Euclidean) distance between endpoints. A higher deviation of TI from 1.0 indicates greater vessel tortuosity. TI values were calculated for each individual vessel and averaged across all vessels within each eye. Images were analyzed in the naïve condition and at 9 months post-injury. Retinal vascular diameter was determined by measuring the widest segment of four selected major retinal vessels in each eye. Measurements were measured from both eyes for each animal (*n* = 10 and 8 eyes for naïve and 9-month post-CHIMERA, respectively).

### Measurement of blood vessel intensity using indocyanine green angiography

The average intensity of blood vessels was evaluated from fundus ICGA images acquired with SD-OCT. Blood vessel profiles (5–7 vessels/eye; naïve, *n* = 8 eyes; 9-months post-CHIMERA, *n* = 10 eyes; total vessels analyzed = 109) were manually outlined using the polygon selection tool in Fiji (ImageJ, NIH) by the same investigator, blinded to the experimental groups. For each eye, all outlined vessels were measured simultaneously to obtain vessel area and mean gray value. To account for acquisition-related differences in brightness, the mean gray values of each eye were normalized to the maximum mean gray value of that eye (set to 100%) to enable comparisons. Higher intraocular variability was interpreted as an indicator of altered vascular circulation, whereas controls were expected to show more homogeneous circulation, reflected by lower variability values.

### Tissue processing

Prior to cardiac perfusion, animals were placed in a sealed chamber and exposed to an overdose of isoflurane, achieved by administration of 5% isoflurane in 100% oxygen in an induction chamber until cessation of spontaneous breathing. This procedure rapidly induces deep anesthesia and loss of consciousness, followed by respiratory and cardiac arrest. Prior to secondary cardiac perfusion, death was confirmed by the absence of spontaneous breathing and heart rate, as well as by the presence of characteristic pallor of the skin in the paws and of the nose, mouth, and tongue. The animals were then perfused with PBS and 4% PFA, followed by careful dissection of the brains and eyes (with ONs intact). The ONs were then cut to separate the two eyes. Eyes were post-fixed in 4% PFA for 1 h. Under a dissection microscope, retinas were gently isolated from the eyecups. To prepare for flat-mount imaging, retinas were cut radially in a star pattern to flatten the tissue. Retinas were then washed three times with PBS to remove residual PFA and to store for further analysis.

The spleen and kidneys were also removed after perfusion. Fat was carefully dissected away from each organ under a dissection microscope, and the wet weight was measured using a laboratory scale.

### Immunostaining

After carefully removing the vitreous using fine forceps and a soft brush, retinas were permeabilized and blocked in 2% normal goat serum with Triton X-100 in PBS for 30 min, replacing the solution every 10 min. Retinas were incubated in primary antibody solution RNA binding protein, multi-splicing (RBPMS; red, Genetex, Cat#: GTX118619; 1:500^75^) in 0.5% PBST (Triton X-100) for 7 days at 4 °C. After three washes, retinas were incubated in the appropriate secondary antibody for 3 days at 4 °C. Following three additional washes, retinas were mounted using a glycerol-based mounting medium. A subset of retinas (*n* = 4 and 3 for naïve and 9 months post-CHIMERA, respectively) were dismounted and immunodetected for IBA1 (green, Wako, Cat#: 019-19741, 1:500^96^) to evaluate microglia numbers.

ONs were washed three times in PBS and then placed in 15% sucrose in PBS for 6 h, followed by transfer to 30% sucrose overnight at 4 °C. ONs were embedded in molds with OCT compound on dry ice. The molds were then equilibrated in the cryostat for 1 h before sectioning. ONs were mounted onto glass slides and sections (15 µm thick) were incubated overnight at 4 °C with primary antibodies in PBST, including GFAP (red, Aves Labs, Cat# GFAP, 1:500^97^), IBA1 (green, Wako, Cat#: 019-19741, 1:500) and CD68 (magenta, Bio-Rad Laboratories, MCA1957, 1:1000^14^), and counterstained with DAPI (blue, 1:1000). Slides were washed three times with PBS and incubated with the appropriate secondary antibodies for 2 h at room temperature. Following three additional washes, slides were mounted using an antifade glycerol-based mounting medium.

### Image acquisition

Images from whole-mount retinas and entire retinal sections were obtained using a 20× objective on a Nikon A1R microscope. Additionally, images from different retinal areas (dorsal, visual streak, and ventral periphery) were acquired to evaluate regional alterations.

### Image quantification of retinal ganglion cells, microglial cells, and GFAP integrated density

For RGC quantification, image processing was performed using NIS-Elements AR (General Analysis 3, GA3), which enables integration of conventional segmentation with AI-assisted tools to create customizable measurement workflows. After selecting the appropriate fluorescence channel (red) corresponding to RGCs, the Bright Spots function was used to identify and mask circular, high-intensity features. The typical diameter parameter was set to 10.0 µm, contrast was set to 0.01, symmetry was set to detect all objects, and the intensity range was defined as 0–255. The Grow feature, which expands pixels outward from detected objects, was disabled. For visualization, the output was configured to display circular regions outlining the detected RGCs. RGC numbers were calculated in dorsal (*n* = 5 and 9 for naïve and 9 months post-CHIMERA, respectively) and ventral (*n* = 5 for naïve and 9 months post-CHIMERA) retinas with the same area (0.64 × 0.64 mm) and divided by 0.4096 mm^2^ to obtain their density (cells/mm^2^). In the pilot experiments conducted to define the CHIMERA parameters, RGCs were quantified in AOIs (0.58 × 0.58 mm) from dorsal and ventral retinas of animals subjected to CHIMERA at 0.5, 1, and 1.5 J (*n* = 2 for 0.5 and 1 J, and *n* = 1 for 1.5 J).

The density of microglia cells (Iba^+^cells) was quantified manually from whole-mount retinas and ON longitudinal sections. For retinas, we extracted 3 retinal areas (dorsal, visual streak, and mid-ventral) with the same frame size (0.5 × 0.5 mm). The dorsal frame was obtained from 2 to 3 mm above the horizontal ON head, the visual streak was obtained from the central region of the visual streak (that corresponds approximately from 1.5 to 2.5 mm below the ON head), and the mid-ventral frame was extracted from approximately 4–6 mm below the ON head (for retinal and ON head position, see^14^). For ON sections, a single frame (1.0 × 0.2 mm) was extracted along the ON axis. Then, all images were manually dotted using image editing software (Adobe Photoshop v26.6.1). The manually annotated microglia cells (10pix round dots representing Iba^+^cells) were subsequently quantified using an automated algorithm in FIJI (NIH^14^). To obtain the density, we divided the number of cells by the image frame area (0.25 and 0.2 mm^2^ for retina and ON images, respectively). From all retinas we used a subset (*n* = 4 and 3 for naïve and 9 months post-CHIMERA, respectively) per group to evaluate the microglia, and we evaluated the density per area. For ONs, we evaluated *n* = 6 for naïve and *n* = 10 for TBI.

Microglial activation in the ON was evaluated by assessing the co-localization of Iba1 and CD68 immunoreactivity. Focal CD68-positive microglial activation was clearly detected in specific branches in most ONs subjected to CHIMERA; however, widespread activation across the entire ON section was not detected. Given the relatively large diameter and complex architecture of the ON of the thirteen-lined ground squirrel, compared with those of mice or rats, only a subset of sections was analyzed. Consequently, additional localized regions of degeneration and associated microglial activation may have been underrepresented. Therefore, microglial activation was quantified as the proportion of ONs exhibiting microglial activation versus those without detectable activation in both experimental and control groups.

Quantification of GFAP immunoreactivity in the optic nerve was performed using ImageJ (FIJI, NIH) by measuring integrated density normalized to the corresponding optic nerve area. To minimize technical variability, only samples that were sectioned on the same day, mounted on the same slide, and processed simultaneously during immunostaining were included. For each optic nerve, a region of interest measuring 0.2 × 1 mm was defined, and fluorescence intensity was quantified using the integrated density function.

### Statistics and reproducibility

All statistical analyses were conducted using GraphPad Prism (version 10.5.0), except for the indocyanine green (ICG) vascular intensity (Levene’s test calculator available from Statistics Kingdom 2017). A comprehensive summary of the statistical tests applied, including results and significance levels, is presented in Supplementary Data 1. OCT measurements of RNFL and total retinal thickness were analyzed using repeated-measures one-way ANOVA and Tukey´s post hoc test for multiple comparisons, as the dataset included pre-injury baseline measurements and follow-up assessments 4.5- and 9-months post-injury. Amplitude and implicit time of the ERG a- and b-waves, as well as flicker response amplitudes (5, 15, and 30 Hz), were analyzed using ordinary one-way ANOVA followed by Tukey’s post hoc test for multiple comparisons, comparing age-matched naïve control animals with CHIMERA-exposed animals. OMR visual acuity measurements were analyzed using ordinary one-way ANOVA and Tukey´s post hoc test for multiple comparisons, as age-matched naïve control animals and CHIMERA-exposed animals were compared. Repeated-measures analysis was not applied because the naïve control animals were not longitudinally matched to the CHIMERA cohort; control animals were assessed at 0 and 9 months, whereas CHIMERA animals were assessed at 3-, 6-, and 9-month post-injury. Pattern ERG amplitudes and implicit times were analyzed using repeated-measures one-way ANOVA and Tukey´s post hoc test for multiple comparisons, consistent with the inclusion of pre-injury baseline measurements and follow-up measurements at 4.5- and 9-month post-injury. Retinal vasculature parameters, microglial cell density (Iba1 cells per unit area) in retina and ONs, GFAP integrated density, and the weight of different organs (spleen and kidneys), were compared between naïve and 9 months post-CHIMERA injury using unpaired two-tailed Student’s *t* tests. The ICG vascular intensity histogram and confidence intervals were plotted using Levene’s test calculator [Internet] and Statistics Kingdom 2017 [cited 18 September 2025] (Available from: https://statskingdom.com/230var_levenes.html and verified using the R software package). RGC (RBPMS) density in the retina was initially analyzed using ordinary one-way ANOVA with Tukey’s post hoc test for multiple comparisons. However, because thirteen-lined ground squirrel RGC density is known to differ between dorsal and ventral retina^14^, and this was not the focus of the present study, direct comparisons between naïve and 9-month post-CHIMERA groups were performed using unpaired two-tailed Student’s *t* tests. Microglial activation (Iba1/CD68 co-localization) was analyzed using Fisher’s exact test by comparing the proportion of ONs exhibiting activated microglia between naïve and CHIMERA-treated groups.

Data are presented as box plots (GraphPad Prism, version 10.5.0), in which the central line represents the median and the upper and lower box boundaries indicate the upper and lower quartiles, respectively. Whiskers extend to the 90th (upper) and 10th (lower) percentiles. Individual data points are shown as dots. Statistical significance is indicated as follows: *p* ≤ 0.05 (*), *p* ≤ 0.01 (**), *p* ≤ 0.001 (***), and *p* ≤ 0.0001 (****).

This study was not formally registered because it was a preclinical animal study, and trial registration is not required for nonclinical research. Details of the study design are included in the Methods section and in the figure legends. The CHIMERA group included five thirteen-lined ground squirrels, with sample size determined by NEI Animal Care and Use Committee (ACUC) limitations on the use of this USDA-protected species in animal research for blast of closed head impact TBI. Since this is primarily a longitudinal study with regards to visual function, structure, and behavior, the number of eyes and ONs was *n* = 10 eyes from 5 animals that satisfied the required sample size (*n* = 10 eyes) calculated using G × Power 3.1.9.7 for an effect size d*z* = 0.9, *α* err prob = 0.05, and Power (1-β err prob): 0.8. The actual power was calculated to be 0.83.

This approach is justified by the diffuse nature of the CHIMERA head injury, which can differentially affect individual visual pathways, and by prior evidence showing that unilateral ON injury does not induce significant contralateral RGC degeneration^76,98^. Although contralateral glial activation has been reported in certain conditions (e.g., glaucoma^99^), this does not necessarily result in visual impairment or contralateral RGC loss, which are the primary anatomical outcomes assessed in this study. Accordingly, pathological changes detected in either eye or ON, whether arising from direct or secondary mechanisms, were considered a consequence of the CHIMERA challenge.

In this study, although the traumatic impact was delivered at the head midline, the biological outcome may differ between sides due to inherent variability in TBI models. Thus, for all groups, both eyes and ONs from each animal were analyzed and treated as independent experimental units (OCT, ERG, PERG, and ex vivo anatomical analysis), except for the behavioral assessment (OMR), where each animal was an experimental unit. While bilateral sampling was performed, staining combinations were not applied systematically across all ex vivo analyses, precluding the use of repeated-measures statistical approaches and limiting the assessment of intra-animal correlations. In some cases, additional naïve animals were included as age-matched animals. Certain analyses were performed on subsets of the available samples; accordingly, the specific sample size for each analysis is provided in the corresponding figure legends and in Supplementary Data 1. Animals were randomly allocated to experimental groups; however, attempts were made to include equal numbers of age-matched male and female animals whenever possible. While we did not observe any anecdotal sex-dependent differences across primary functional, behavioral, or anatomical outcome measures, we were unable to perform a statistical analysis for sex effects due to limited sample size. Therefore, data from males and females were pooled for all analyses to focus on injury-dependent effects and to increase statistical power.

Investigators who performed comparative image and data analysis were blinded to the group (control vs. TBI) using animal ID numbers assigned by the veterinary facility.

### Reporting summary

Further information on research design is available in the Nature Portfolio Reporting Summary linked to this article.

## Supplementary information

Supplementary Information

Description of Additional Supplementary Files

Supplementary Data 1

Reporting Summary

Transparent Peer Review File

## Data Availability

The raw datasets supporting the findings and conclusions of this study are included within the article and its Supplementary Materials. Original files are available upon request in compliance with the NIH Data Management and Sharing Policy.
