# Supplementary material for: Impact-acceleration head injury results in optic neuropathy in the thirteen-lined ground squirrel
Source: Commun Biol. 2026 Jun 9;9:1217. doi: 10.1038/s42003-026-10397-4 (PMC13586358; doi:10.1038/s42003-026-10397-4)
Supplement: Supplementary file 1 [file 42003_2026_10397_MOESM1_ESM.pdf]

SUPPLEMENTARY INFORMATION

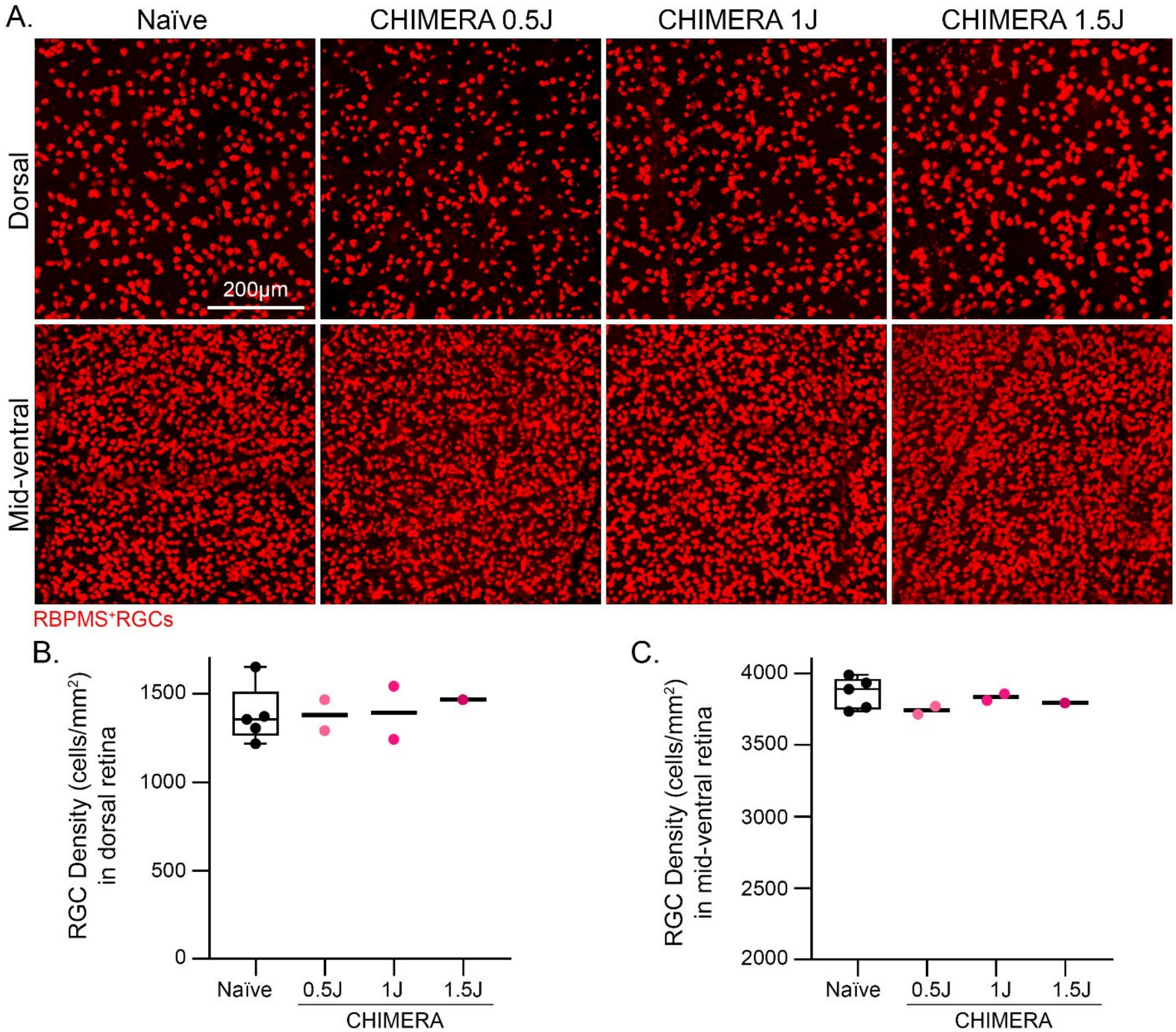

<u>Supplementary Figure 1</u>. Pilot CHIMERA study at 21 days post-CHIMERA induction. **(A)** Representative RBPMS immunofluorescence images of Retinal ganglion cells (RGCs) in flat-mounted thirteen-lined ground squirrel retinas from naïve animals and animals exposed to 0.5, 1, and 1.5 J CHIMERA injury. Images show retinal regions from the dorsal (top row) and mid-ventral (bottom row) retina. **(B)** Quantification of RGC density in the dorsal retina, showing no apparent differences among naïve and CHIMERA-injured groups (0.5, 1, and 1.5 J). **(C)** Quantification of RGC density in the mid-ventral retina, also showing no apparent differences among naïve and CHIMERA-injured groups (0.5, 1, and 1.5 J). Sample sizes: n = 5 [3 male and 2 female] retinas for naïve, n = 2 for CHIMERA 0.5 J [2 female] and 1 J [2 female], and n = 1 [1 female] retina for CHIMERA 1.5 J. No statistical analyses were performed due to the limited sample size (as approved by the Institutional Animal Care and Use Committee) in this pilot study aimed at establishing the CHIMERA paradigm in the thirteen-lined ground squirrel model. Whiskers in the Naïve groups **(B–C)** extend to the 10th and 90th percentiles.

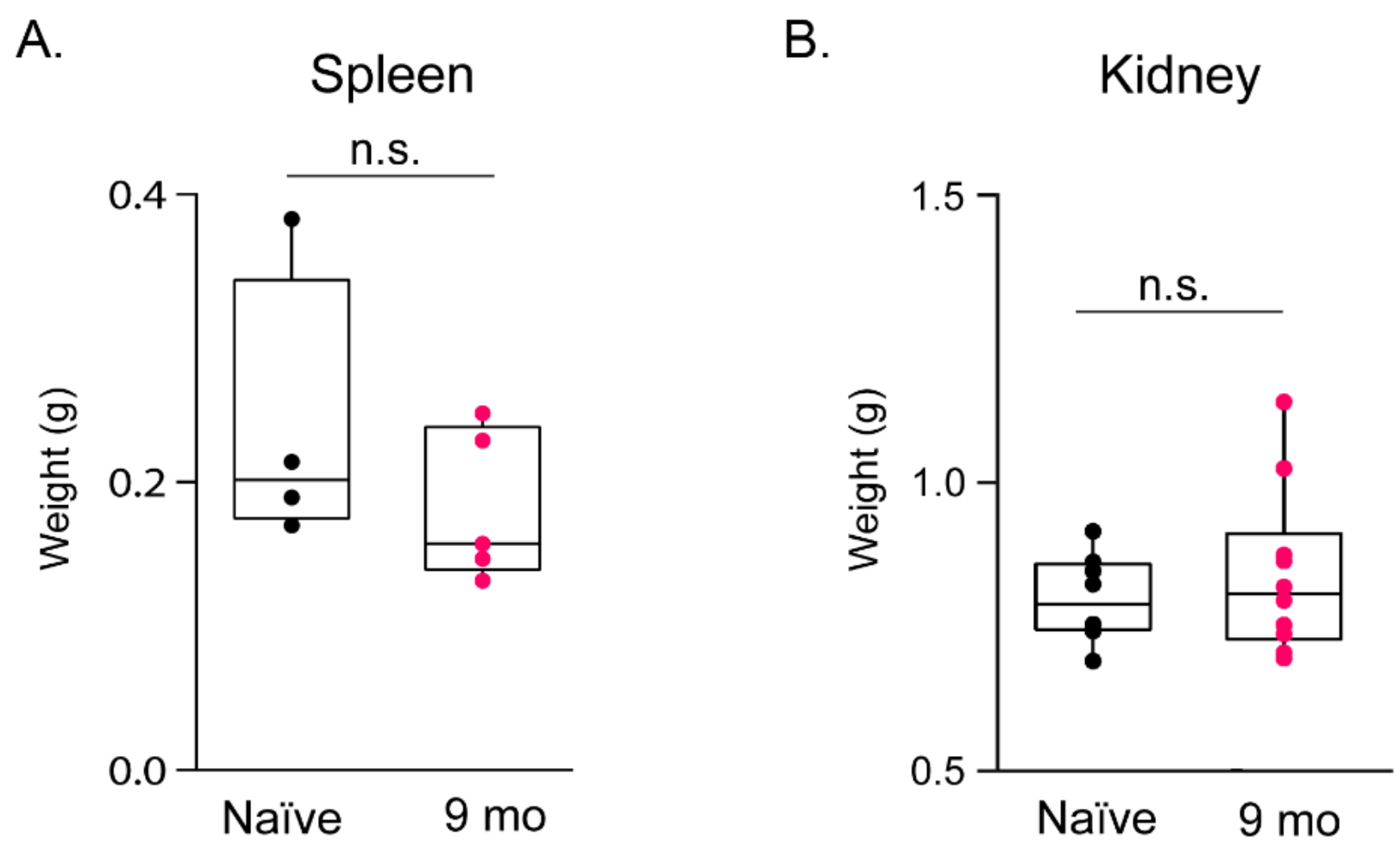

<u>**Supplementary Figure 2.**</u> **Organ weights in the thirteen-lined ground squirrels in naïve and at 9-months post-CHIMERA induction**. Wet weights of spleen **(A)** and kidneys **(B)** in naïve controls (black) and CHIMERA-exposed animals at 9 months post-injury (magenta). Sample size, n=4 [2 male and 2 female] and 5 [3 male and 2 female] spleens, and n=8 [4 and 4 from male and female animals] and 10 [6 and 4 from male and female animals, respectively] kidneys for naïve and 9-months post-CHIMERA groups, respectively; unpaired two-tailed Student's t-test, n.s., not significant). The whiskers on all boxplots shown **(A-B)** extend to the 90th (upper) and 10th (lower) percentiles. See Supplementary Data for statistical details.
