# Supplementary material for: Impact-acceleration head injury results in optic neuropathy in the thirteen-lined ground squirrel
Source: Commun Biol. 2026 Jun 9;9:1217. doi: 10.1038/s42003-026-10397-4 (PMC13586358; doi:10.1038/s42003-026-10397-4)
Supplement: Supplementary file 3 [file 42003_2026_10397_MOESM5_ESM.pdf]

nature portfolio

Peer Review File

Impact-Acceleration Head Injury Results in Optic Neuropathy in the Thirteen-Lined Ground Squirrel

Corresponding Author: Dr Kiyoharu Miyagishima

This file contains all reviewer reports in order by version, followed by all author rebuttals in order by version.

Version 0:

Reviewer comments:

Reviewer #1

(Remarks to the Author)
This submission characterizes changes to the visual system following repeated mild, diffuse TBI induced with the CHIMERA model in the thirteen-lined ground squirrel. Damage to the optic nerve, retina and visual pathway have been established in multiple models of closed head TBI. The authors make the case that because this squirrel species has cone dominated retina, they more closely resemble humans than do rodents (nocturnal animals with rod dominant retina) and would provide a more clinically relevant model. The authors examine visual responses and retinal functional measures as well as histology of the retina and optic nerve. While the premise for developing a model using the ground squirrel is interesting, the study does not contain the necessary controls. This fundamental flaw prohibits drawing meaningful conclusions regarding the effect of repeated mild TBI over a 9-month postinjury period. There are also numerous substantial issues with the experimental approach and rigor, analyses and interpretations. Some of these issues, although not an exhaustive list, are provided below.

The controls are stated to be age-matched, but their age is not provided and it is not clear whether they are aged-match at the time of the induction of injury (6.5 months old) or if they are age-matched to the 9 month euthanasia time point for the CHIMERA group (15.5 months old). Further, the control animals do not appear to be monitored over time, to provide age-matched control data to parallel the time course of the injured squirrels (measurements at 3, 4.5, 6 and 9 months after TBI). This is a fundamental flaw in the design. There is no apparent reason why assessments could not have been done repeatedly in the control (naïve) animals. Without parallel measurements in age-matched controls at each time point, the results in the study could merely reflect age-related changes. Changes in the CHIMERA group cannot be attributed to TBI with the current design.

Other issues:
1. The study is underpowered, with 4 controls and 5 injured animals.
2. The authors should not use the term 'control' or 'naïve' to refer to baseline of injured squirrels These are not equivalent in terms of the design and confuse the interpretation (see for example, Figure 3D where graph is labeled as 'naïve' but legend describes this group as 'pre-injury'). Data are not presented consistently; for example, some outcomes appear to lack the naïve control group entirely (see Figure 2D).
3. The authors fail to clearly describe what constitutes 'n' for each outcome measure and to report the n's per group for each outcome. While the methods state there are 5 CHIMERA squirrels and 4 naïve controls, the authors refer to some additional controls that are added at later time points. The numbers, sex and ages of these must be specified as well as whether these were used for all outcomes or only selected outcomes. If the latter, a strong rationale should be provided. Oftentimes there are many more data points than 5 in injured groups (see Figures 2, 3, 4) without adequate description of how these larger groups were derived. If multiple measures are taken within a given animal (as in from the two eyes/nerves or multiple measurements within one sample), these cannot be considered as independent samples, and must either be averaged to yield one data point per animal or treated as a repeated measure within an ANOVA design.
4. The authors state that males and females were used. Given the potential for sex-dependent responses, the number of males and females should be reported. The proportion of males:females should be comparable in control (naïve) and CHIMERA groups and the responses differentiated in the graphs when possible (e.g. different color or shape symbols within the group).
5. The manuscript lacks a statistical analysis methods section. Only in a few cases are statistical approaches detailed (with text in results or figure legend). A comprehensive statistical analysis section should outline the approach used for each

outcome measure. Further, statistical methods are at times inconsistent (e.g. the data in Fig 3B and 3D analyzed using different approaches despite identical experimental constructs). Some data are interpreted in the absence of any statistical analysis (e.g. Fig 1I, J). The approaches do not incorporate the use of repeated sampling within an animal and do not consistently incorporate the repeated measures design within the CHIMERA group. For any ANOVA, the F test results, including p value, should be reported.
6. A number of datasets are overinterpreted. When a statistical test indicates no difference, a difference should not be reported. For example, there is no difference in spleen weight (Fig 1D) but the authors report a decrease in spleen weight. This supposed difference is driven by one naïve animal with a spleen nearly 2x the size of the other naïve squirrels, which seems highly abnormal. Thus, conclusions about changes in spleen weight due to TBI are unwarranted. See also Fig 2F, 2G where there is no statistical difference (although the statistical test was not described) but the authors report a difference.
7. More detail should be provided about housing. There is no assurance that naïve controls were housed for similar duration/time of year as squirrels designated for CHIMERA. This is especially important given the authors desire to link injury to disruption of seasonal/metabolic patterns. Further, it is unclear whether the 'hibernating' and 'nonhibernating' animals in Fig 1C are all naïve squirrels. It is not clear how long before being assigned to injury the squirrels were housed in the vivarium. This is critical to their characterization as 'non hibernating'. It appears the injury was induced at the beginning of the 'hibernation season.' The authors acknowledge that the timing of initiation of torpor (indicating hibernation) varied across animals through the fall and winter, so it is not clear how the authors know whether the squirrels designated for CHIMERA would or would not have hibernated.
8. The conclusions about weight changes are complicated by the apparently lack of body weight measurements just prior to injury in the CHIMERA cohort (see Fig 1C). Thus, the large weight change between 1 month prior to injury and the first post-injury measurement in some injured squirrels cannot reliably be attributed to the injury as all or some may have occurred in the one month prior to the injury.
9. The statement that prior reports show a single impact is insufficient to generate injury pathology (Results 1st paragraph) is misleading. Many papers have documented pathology with a single impact, while others report no pathology. This is highly dependent on factors such as the type of model, injury location, injury severity, and outcome parameter.
10. There is no description of the quantification approach used for optic nerve immunostaining (or CD68 immunostaining). The description of quantification of RGCs is incomplete and should specify factors such as the magnification used for imaging, size or number of fields assessed, how these were selected in a unbiased fashion, and method for cell counting. For example, was this object counting within the analysis software? If so, was there an intensity threshold used for cell detection or size inclusion/exclusion criteria?
11. Exclusion of a data point for the analysis for data in Figure 3D is not appropriate. The groups are too small to robustly detect 'outliers' and this data point is well within the experimental range across the controls and the injured animals at different time points.
12. Speculation about early changes in GFAP expression in optic nerves should be removed from the methods.
13. It is unclear what is meant by 'collateral damage' on p. 16
14. There is no mention of anesthesia of the squirrels prior to cardiac perfusion (p. 16). Is this an omission?
15. Please indicate in the methods whether animals were returned to isoflurane between the two CHIMERA injuries. Specify whether naïve squirreals received a similar duration exposure to isoflurane as did the CHIMERA cohort.

Reviewer #2

(Remarks to the Author)
The paper introduces a model of TBI (procedural and animal model) that is suitable for comparison with primates. It is generally well written and is sufficient for the aims of the study, although further experimental evidence could have been provided. why not cross-sectional analysis of retina to show changes in layers. It would be good to be added to the result sections.
Please further comment in the discussion on the fact that a lot of changes are not significant, can authors think of more repetition for injury to see higher chances of big differences? Or just adding numbers will resolve it

Detailed comments below;
Line 53: species name should be in Italics
Line 76: unusual to abbreviate species names in scientific papers, either TLGS as an abbreviation of species name is rather unusual, Anyhow use TLGS or squirrels, consistently in the text not randomly use one of the two
Line 122-124: this statement is not well observed in the figure 1C, better explanation or clarity on what stage these 3 animals are different would be helpful
Line 156; show significance on the graph Fig 1I-J
Line 162: Fig 2 not 2A as ERG is not shown in 2A
Line 172: CHIMERA???
Line 176: retinal ganglion cells and their axons do not contribute to the ERG recording.correct this sentence then
Fig 2E: please indicate a and b wave on the graph, Fig 2F: why a and b wave were capitalized in the graph but not in the text. they should be the same
Fig 3C: in C different groups have to be identified
Fig 4A: 9 months looks like the image has a different magnification, correct it. Relevant counting method is not described
Line 257-258: ERG does not measure Ganglion cells and their functions, so this statement is not correct
Line 264: may (repeated word)
Line 266: full stop at the end of sentence
Line 419: sudden change of font

Reviewer #3

(Remarks to the Author)
Please see the attached detailed review, as I want to preserve the outline formatting. Thank you.

Version 2:

Reviewer comments:

Reviewer #1

(Remarks to the Author)
The authors have made many changes to the manuscript in response to the reviewers' comments. However, there remain numerous points that require clarification, particularly regarding the statistical analysis, reporting of the results and interpretation of the findings.

1. The conclusions in the Abstract should be more carefully written to convey accurately the findings. The authors write "Longitudinal ophthalmic assessments revealed retinal thinning and significant reductions in RGC function, along with persistent deficits in visual acuity. Histological analysis confirmed dorsal RGC loss and late-stage changes in ON glia." What are the reductions in RGC function that are referred to here? There were no significant changes in retinal functional measures in Figure 2. Visual acuity deficits were observed at 3 months but not 6 or 9 months, so the characterization of these deficits as 'persistent' is questionable. The changes in ON glia were only for astrocytes, not microglia, so it would be more precise to state this as 'changes in ON astrocytes'.
2. The housing conditions are not detailed for the subset of ground squirrels that were moved to the hibernaculum to allow hibernation (in support of collecting weight data in Figure 1?). The alterations to vivarium temperatures (shown in Figure 1) were not described in the housing section.
3. The statistical analysis section and reporting within the text/legends is not complete.
a) It would be easier for the reader to follow if references were provided to the data figures for each test described. For example, "OCT measurements of RNFL and total retinal thickness (Figs 2B and 2C) were analyzed using repeated-measures one-way ANOVA in GraphPad Prism (version 10.5.0), as the dataset included pre-injury baseline measurements and follow-up assessments 4.5- and 9-months post-injury." This would also help the authors verify that they have provided methods for each dataset.
b) In the previous sentence, statistical software is referenced for this one test, but not for any of the other tests. If all tests were run in GraphPad Prism v 10.5.0, this statement should stand alone, not in association with one test.
c) ANOVA results are not consistently reported. The authors should report all ANOVA F test results and p values. F tests should be reported as $F(x,y)=z$, $p=xx$ or $F_{x,y}=z$, $p=xx$. For 2-way ANOVAs or 1-way ANOVAs with repeated measures, both main effect and interaction ANOVA results should be provided. This can be done in the results text, the figure legends or in a table.
d) Post-hoc t-tests used for ANOVAs are not described. Consideration should be given to correcting for multiple comparisons.
e) Lines 167-169, the authors state that RNFL thickness "decreased progressively with time" despite no significance at either time point relative to baseline and no notable change between 4.5 and 9 months. This is an overinterpretation. Further, no ANOVA results are reported. (see point c).
f) Authors should be clear about whether p values reported are for the ANOVA or the posthoc tests. For example, on page 5 the authors write "In contrast, total retinal thickness was significantly reduced at 4.5 months post-injury (Figure 2C; $p=0.0014$; repeated-measures ANOVA, n=10 eyes). By 9 months post-injury, retinal thickness was no longer significantly different from baseline but was significantly increased relative to the 4.5-month time point ($p=0.008$) Figure 2C; repeated-measures ANOVA, $F=15.34$, $p=0.0002$)," This is confusing, because the there should only be one repeated measures ANOVA run for Figure 2C, which should be reported (F test and p value for the ANOVA) prior to reporting posthoc t-test p values. It is unclear whether the two different p values refer to the ANOVA or to the posthoc tests.
g) It is unclear how data in Figure 2 F-I were analyzed, despite a reference to no "significant" changes on line 186, which implies statistical analyses. The statistical tests should be described in the Statistical analysis section and the results reported (i.e. non-significant ANOVAs for main/interaction effects).
h) There is no description of the statistical analysis for data in Fig 2 M-O.
i) On page 6, the authors' statement that "This reduction in OMR persisted at 6- (1.26 ± 0.32 cyc/deg) and 9-months (1.36 ± 0.25 cyc/deg) post-injury" is not supported by the statistical analysis which shows no difference, as they report: "… although the difference at these later time points no longer reached statistical significance." Given the very small n for this study, the authors must be more cautious about overinterpreting nonsignificant shifts in distribution. The title of this section (lines 200-201) should also be modified accordingly.
j) On page 7, in describing the PERG data, the authors describe one posthoc result, then the overall ANOVA, then another posthoc result. It would be clearer to first report the overall ANOVA, and then the posthoc comparisons.
k) The statistical analysis section says "Retinal vasculature parameters were compared between baseline pre-injury images and 9 months post-CHIMERA injury using Student's t tests." but Figure and Figure legends describe the control group as naïve not pre-injury. There is also a redundant sentence fragment in this part of the statistical analysis section.
l) Please specify in the Statistical Analysis section whether student's t-tests were 2-tailed or 1-tailed, paired or unpaired. Then this information does not need to be provided in the Results text.
m) The authors, in their response to the previous critique that two eyes or nerves in the same animal are not independent

measures, provide a statement in their 'definition of n' that "where applicable, bilateral measure were analyzed using repeated-measures ANOVA designs". This approach does not seem to have been incorporated into any of the analyses. Further, the authors statement that the injury is "equivalent" to both eyes and nerves of one animal is not supported by any evidence and is contradicted by their statement that the TBI can "differentially affect each visual pathway". Further, if the injury (mechanically) is equivalent to both sides of the visual system, as the authors suggest, this is in support of the necessity of treating the left and right samples as repeated measures rather than independent samples. That is, the responses within an animal may be more similar than those across animals. This can be tested statistically, but the fact that some animals were sampled once (R or L) while others were sampled bilaterally (R and L) as revealed in the supplementary Excel file provided with this revision, makes testing this assumption virtually impossible. The lack of systematic sampling is a limitation of the study design that needs to be included.
4. In the legend for Figure 2, the box and whisker plots are described as "Error bars represent the 90th (top) and 25th (bottom)". This is a nonstandard description and seems incomplete and in error. The middle line and top and bottom of the box should be defined (often these represent median and quartiles), and the whiskers (error bars) should be defined (often these represent the 95th percentile, or maybe the 90th percentile in this case?). This explanation should be included in each figure containing box plots. Alternatively, the explanation could be in the Statistical Analysis section to describe how all box plots are represented.
5. On page 5-6, the authors state "By 9 months post-injury, retinal thickness was no longer significantly different from baseline but was significantly increased relative to the 4.5-month time point …suggesting that prolonged retinal inflammation may contribute to increased cellular thickness at this later stage." It is unclear how any inference about inflammation can be drawn from retinal thickness measurements. This speculative comment should be removed from the Results.
6. Statements (page 8) such as "This likely reflects the fact that microglia are primarily recruited to sites of active degeneration and debris clearance. Following the initial injury and subsequent wave of neurodegeneration, the inflammatory response diminishes in regions where RGCs have already been lost, leading to a reduced microglial presence over time" are speculative interpretation and belong in the Discussion, not the Results.
7. Similarly, "certain ON sections display microglial activation, possibly associated with localized axonal degeneration." The latter part of this sentence is speculation that could be provided in the Discussion but not the Results.
8. The statement "TBI-exposed thirteen-lined ground squirrels exhibited long-term deficits in RGC function, as evidenced by reduced pattern ERG amplitudes and delayed implicit times" on page 9 is not supported by the data, which showed no change in implicit times. Further, the PERG data shows a delayed decrease in RGC function, which is somewhat different than 'long-term deficits' which could be interpreted as deficits that persist for a long time.
9. The statement that " in vivo anatomical evaluation using OCT revealed a gradual thinning of the RNFL" is not supported by the data, which 1) does not show a statistically significant change and 2) shows an equivalent median at 4.5 and 9 months. There is no acute data, so the authors do not know if this nonsignificant decrease was 'gradual'.
10. The statement "…total retinal thickness was revealed to be significant at 4.5 months post-injury and a subsequent increase in thickness at 9 months, possibly reflecting an immune cell-mediated response. This degeneration…" is also highly speculative. What evidence is there of immune cell mediated increase in retinal thickness? The authors data show no change in retinal glia. Further, what evidence is there that the decreased retinal thickness represents 'degeneration', if the change is transient? RGC dysfunction does not equate to degeneration.
11. The statement "These events were accompanied with subtle alterations in retinal vascular branching and changes in microglial markers in the ON" is not accurate, as there were no changes in microglial markers (Iba1) in the retina or ON. The CD68 labeling was reported anecdotally. If the authors wish to make a claim about changes in microglial in the ON, they need to perform a more complete examination of CD68 immunolabeling.
12. It is unclear how the data in this study are consistent with reports of axonal injury in rodent TBI (lines 312-315), since no assessment of axonal pathology was performed and GFAP expression decreased, which is opposite of the typical response to white matter injury.
13. Given the authors admission that availability of the ground squirrels and their protected status constrained their experimental design, these considerations should be provided as limitations to the model in the Discussion.

Reviewer #2

(Remarks to the Author)
I am happy with the revisions made and have no further comments.

Reviewer #3

(Remarks to the Author)
I thank the Authors for the comprehensive responses to all the Editor's and Reviewers' comments. I am grateful for the Reviewers for catching things I missed.

The Rebuttal responses to MY particular concerns have been met in the revision.

If the other Reviewers feel the same way, I consider this Revision to be a success worthy of publication.

Nevertheless there are two small points:

1. The word "scull" should be spelled "skull".

2.The new text at the Revision's Lines 571-2 should reads: "...despite multiple publications using mice that report damage

from one impact(92-96)" [add "using mice"]. It's for a different paper I'm sure, but I wonder how much the difference in skull morphology plays in the differential susceptibility to a single impact.

Open Access This Peer Review File is licensed under a Creative Commons Attribution 4.0 International License, which permits use, sharing, adaptation, distribution and reproduction in any medium or format, as long as you give appropriate credit to the original author(s) and the source, provide a link to the Creative Commons license, and indicate if changes were made.
In cases where reviewers are anonymous, credit should be given to 'Anonymous Referee' and the source.
The images or other third party material in this Peer Review File are included in the article's Creative Commons license, unless indicated otherwise in a credit line to the material. If material is not included in the article's Creative Commons license and your intended use is not permitted by statutory regulation or exceeds the permitted use, you will need to obtain permission directly from the copyright holder.
To view a copy of this license, visit https://creativecommons.org/licenses/by/4.0/

DEPARTMENT OF HEALTH & HUMAN SERVICES

Public Health Service

National Institutes of Health
National Eye Institute
Bethesda, Maryland 20892

Title: Impact-Acceleration Head Injury Results in Optic Neuropathy in the Thirteen-Lined Ground Squirrel

Comments:

Reviewer #1 (Remarks to the Author):

This submission characterizes changes to the visual system following repeated mild, diffuse TBI induced with the CHIMERA model in the thirteen-lined ground squirrel. Damage to the optic nerve, retina and visual pathway have been established in multiple models of closed head TBI. The authors make the case that because this squirrel species has cone dominated retina, they more closely resemble humans than do rodents (nocturnal animals with rod dominant retina) and would provide a more clinically relevant model. The authors examine visual responses and retinal functional measures as well as histology of the retina and optic nerve. While the premise for developing a model using the ground squirrel is interesting, the study does not contain the necessary controls. This fundamental flaw prohibits drawing meaningful conclusions regarding the effect of repeated mild TBI over a 9-month postinjury period. There are also numerous substantial issues with the experimental approach and rigor, analyses and interpretations. Some of these issues, although not an exhaustive list, are provided below.

The controls are stated to be age-matched, but their age is not provided and it is not clear whether they are aged-match at the time of the induction of injury (6.5 months old) or if they are age-matched to the 9 month euthanasia time point for the CHIMERA group (15.5 months old). Further, the control animals do not appear to be monitored over time, to provide age-matched control data to parallel the time course of the injured squirrels (measurements at 3, 4.5, 6 and 9 months after TBI). This is a fundamental flaw in the design. There is no apparent reason why assessments could not have been done repeatedly in the control (naïve) animals. Without parallel measurements in age-matched controls at each time point, the results in the study could merely reflect age-related changes. Changes in the CHIMERA group cannot be attributed to TBI with the current design.

Re: We thank the reviewer for this careful assessment and appreciate the opportunity to clarify the experimental design and control comparisons.

For behavioral measures such as optomotor response (OMR), control animals consisted of naïve, uninjured squirrels that were age-matched to the experimental animals at the time of CHIMERA induction (6.5 months of age). These same control animals were also assessed longitudinally and measured again 9 months later, corresponding to the 9-month post-injury time point in the CHIMERA group. Because no age-related differences were observed over this relatively short interval, these data were previously pooled. In response to the reviewer's concern, we have now separated control data by time point and revised the figures and figure legends accordingly to explicitly indicate the age at assessment.

For other outcome measures where only a single comparison was performed (e.g., CHIMERA vs. control at 9 months post-injury), the control animals were age-matched to the CHIMERA group at the terminal time point (15.5 months of age). We have clarified this explicitly in the Methods and figure legends.

Importantly, we have conducted longitudinal visual function and structural studies in both mice and ground squirrels. We are currently preparing a separate manuscript detailing age-related changes. In that study, squirrels were followed for up to 4 years of age.

Based on these data, the 9-month interval examined here (6.5 to 15.5 months of age) is insufficient to produce measurable age-related changes in visual function, as assessed by PERG amplitudes, ERG amplitudes, and OCT thickness. In our previous analysis, ERG a-wave and b-wave amplitudes did not differ significantly between the 4.5- and 9-month time points; therefore, these data were combined and reported as a single control group. In the current analysis, the data are separated by age, and we find that the previously reported slowing of the ERG responses is no longer significant at 9 months. This correction has been made in the revised manuscript.

The squirrels only breed once a year (within the first two weeks from emerging from hibernation) and are obtained from the University of Wisconsin. We did not obtain any new squirrels this year 2025 due to the freeze in purchases through the Office of Logistics and Acquisition (OLAO) in the federal government.

Finally, ground squirrel availability imposes practical constraints on experimental design. Animals are obtained from the University of Wisconsin and breed only once per year, within a narrow window shortly after emergence from hibernation. Additionally, no new animals were obtained in 2025 due to a federal government-wide freeze on purchases through the Office of Logistics and Acquisition (OLAO). Despite these constraints, the inclusion of age-matched terminal controls, longitudinal assessments of the CHIMERA-injured animals before and after injury, and insights from a prior aging study in squirrels (manuscript in preparation) ensures that the observed changes in the CHIMERA group cannot be attributed to aging alone.

Other issues:
1. The study is underpowered, with 4 controls and 5 injured animals.

As this is primarily a visual study characterizing the effects of traumatic optic neuropathy in the TLGS, we leveraged data from both eyes. Both eyes are expected to be equivalently affected due to the precise central localization of the piston impact in the CHIMERA device.
We selected (n=4) animals to serve as controls (8 eyes) to be compared to the animals subjected to CHIMERA (n=5) animals (10 eyes). Because squirrels are a USDA-protected species, the NIH Animal Care and Use Committee requested that we limit the number of animals used in this study. To address the reviewer's concerns we have reperformed the statistical analysis using repeated-measures ANOVA where appropriate.

2. The authors should not use the term 'control' or 'naïve' to refer to baseline of injured squirrels These are not equivalent in terms of the design and confuse the interpretation (see for example, Figure 3D where graph is labeled as 'naïve' but legend describes this group as 'pre-injury'). Data are not presented consistently; for example, some outcomes appear to lack the naïve control group entirely (see Figure 2D).

Re: The authors thank the reviewer for asking for clarity. We have gone back through the figures and text to make sure that we are more precise in our descriptions and to not use these terms interchangeably. We will use the terms "baseline" or "pre-injury" to refer to the animals in the

CHIMERA condition before being exposed to the CHIMERA paradigm. We will use the term "naïve" to refer to animals in the control condition that are never exposed to CHIMERA.

3. The authors fail to clearly describe what constitutes 'n' for each outcome measure and to report the n's per group for each outcome. While the methods state there are 5 CHIMERA squirrels and 4 naïve controls, the authors refer to some additional controls that are added at later time points. The numbers, sex and ages of these must be specified as well as whether these were used for all outcomes or only selected outcomes. If the latter, a strong rationale should be provided. Oftentimes there are many more data points than 5 in injured groups (see Figures 2, 3, 4) without adequate description of how these larger groups were derived. If multiple measures are taken within a given animal (as in from the two eyes/nerves or multiple measurements within one sample), these cannot be considered as independent samples, and must either be averaged to yield one data point per animal or treated as a repeated measure within an ANOVA design.

Re: The authors thank the reviewer for asking for clarity.

Definition of n
For all outcome measures, n represents the number of individual animals unless otherwise explicitly stated. Where applicable, bilateral measures were analyzed using repeated-measures ANOVA designs. All statistical analyses have been rechecked and revised to ensure appropriate handling of repeated measures.

Experimental Groups

The CHIMERA-injured group consisted of five squirrels:
1799 (female), DOB: 5-20-24
1803 (female), DOB: 5-21-24
1812 (male), DOB: 5-22-24
1813 (male), DOB: 5-22-24
1814 (male), DOB: 5-22-24

The naïve (uninjured) control group consisted of four squirrels:
1846 (female), DOB: 6-6-24
1827 (male), DOB: 5-23-24
1779 (male), DOB: 5-20-24
1833 (female), DOB: 5-23-24

The naïve (uninjured) control group (Hibernating (cyan), Figure 1C) consisted of 6 squirrels:
1776 (male), DOB: 5-20-24
1817 (male), DOB: 5-20-24
1824 (male), DOB: 5-20-24
1845 (female), DOB: 6-4-24
1790 (female), DOB: 5-20-24
1804 (female), DOB: 5-21-24

Sex and age at the time of assessment are now explicitly reported in the Methods.

Age Matching and Cohort Consistency
Ground squirrels hibernate for nearly six months per year and therefore produce only a single litter

annually. Mating occurs within a narrow window immediately following emergence from hibernation, and offspring must mature rapidly before the next hibernation period. Consequently, all animals born within a given year—across different mating pairs—are born within an approximately one-month window (typically April through early May). As a result, animals obtained from the same cohort differ in age by no more than one month, ensuring a high degree of age consistency across experimental and control groups.

Use of Controls Across Outcome Measures

There are no age-dependent changes in OMR, PERG, ERG, or OCT retinal thickness in squirrels between 6.5 months (time of injury) and 15.5 months (terminal collection), based on our prior longitudinal aging study in this species (manuscript in preparation). Accordingly, for these functional measures, longitudinal tracking of naïve controls was not required, as each CHIMERA-injured animal served as its own pre-injury baseline control. Statistical analyses for these outcomes have been revised to use repeated-measures ANOVA where appropriate.For outcome measures assessed only at the terminal time point, age-matched naïve controls (15.5 months old) were used for comparison.

Clarification of Apparent Sample Size Inflation

Figures in which more data points appear than the total number of animals reflect multiple measurements obtained within the same animal (e.g., bilateral eyes). These data points are now clearly identified as within-animal measures and are statistically treated as repeated measures. The analyses no longer treat these data as independent biological replicates and the figures have been updated to reflect the new statistical analysis.

Aging Reference Data

As noted above, we have previously characterized age-related changes in visual and retinal outcomes in both squirrels and mice and are preparing a separate manuscript describing interspecies differences. Data confirming the absence of age-dependent effects in squirrels for PERG and OCT retinal thickness over the relevant age range are provided to the reviewer for reference only and are not intended for inclusion in the present manuscript.

Representative measured values at 12 months of age (naive squirrels, for reference only):

PERG: 92.29 ± 9.51 µV (amplitude) @ 1 year old.
OCT: 283.22 +/- 4.86 µm (total retinal thickness) @ 1 year old. Measured with a 30 degree lens
Note: the data in this manuscript was measured using the 55 degree (wide field) lens.

In the current manuscript we now cite Follett et al (2025) that performed a longitudinal study on OCT thickness in thirteen lined ground squirrel earlier this year and concluded that the retinal thickness of elderly animals (5-8 years old) was comparable to younger eyes (12 months old).

Figure-Specific Clarifications

Figure 2A–C

OCT measurements were obtained from n = 5 CHIMERA-injured squirrels at baseline (pre-injury), 4.5 months post-injury, and 9 months post-injury. Measurements were taken from both left and right eyes, yielding 10 data points per time point. These bilateral measurements were treated as repeated measures within animals.

Figure 2D–O
Panels depict averaged responses from the 10 eyes at each time point (baseline, 4.5 months post-injury, and 9 months post-injury).

Figure 3B
Naïve OMR data were obtained from a separate group of age-matched, uninjured squirrels. These animals were assessed at 0 months and again at 9 months to demonstrate the absence of age-related changes in visual acuity. Data from these two time points are now displayed separately to make this distinction explicit.

Figure 3C
Squirrel identification numbers and left (L) and right (R) eyes are now labeled. The x-axis has been corrected to "Time (months)."

Figures 3D, 3E, 3G, 3H, and 3I
Baseline measurements are now explicitly labeled as "Pre-injury."

4. The authors state that males and females were used. Given the potential for sex-dependent responses, the number of males and females should be reported. The proportion of males:females should be comparable in control (naïve) and CHIMERA groups and the responses differentiated in the graphs when possible (e.g. different color or shape symbols within the group).

The authors thank the reviewer for requesting clarification. The proportion of males to females was comparable between the CHIMERA-injured and naïve control groups. No sex-dependent differences were observed for any outcome measures; therefore, data from males and females were pooled for all analyses. The number of males and females represented in each group has now been clarified in the Methods.

5. The manuscript lacks a statistical analysis methods section. Only in a few cases are statistical approaches detailed (with text in results or figure legend). A comprehensive statistical analysis section should outline the approach used for each outcome measure. Further, statistical methods are at times inconsistent (e.g. the data in Fig 3B and 3D analyzed using different approaches despite identical experimental constructs). Some data are interpreted in the absence of any statistical analysis (e.g. Fig 1I, J). The approaches do not incorporate the use of repeated sampling within an animal and do not consistently incorporate the repeated measures design within the CHIMERA group. For any ANOVA, the F test results, including p value, should be reported.

Re: The authors thank the reviewer for raising this missing section. The suggested changes will be incorporated to the text and figures.

"Statistical Analysis
OCT measurements of RNFL and total retinal thickness were analyzed using repeated-measures one-way ANOVA in GraphPad Prism (version 10.5.0), as the dataset included pre-injury baseline measurements and follow-up assessments 4.5 and 9 months post-injury. OMR visual acuity measurements were analyzed using one-way ANOVA, as age-matched naive control animals and CHIMERA-exposed animals were compared. Repeated-measures analysis was not applied because the naïve control animals were not longitudinally matched to the CHIMERA cohort; control animals were assessed at 0 and 9 months, whereas CHIMERA animals were assessed at 3, 6, and 9 months post-injury. Pattern ERG amplitudes and implicit times were analyzed using repeated-measures one-

way ANOVA, consistent with the inclusion of pre-injury baseline measurements and follow-up measurements at 4.5 and 9 months post-injury. Retinal vasculature parameters were compared between baseline pre-injury. Retinal vasculature parameters were compared between baseline pre-injury images and 9 months post-CHIMERA injury using Student's t tests. Levene's test was used to compare mean absolute deviations as a measure of variability in indocyanine green (ICG) intensity profiles. Quantification of retinal ganglion cells (RGCs) and microglia (cells per unit area) was compared between naïve (uninjured) animals and animals at 9 months post-CHIMERA injury using Student's t tests. Glial fibrillary acidic protein (GFAP) intensity in optic nerve images was quantified in ImageJ as integrated density and compared using Student's t tests."

6. A number of datasets are overinterpreted. When a statistical test indicates no difference, a difference should not be reported. For example, there is no difference in spleen weight (Fig 1D) but the authors report a decrease in spleen weight. This supposed difference is driven by one naïve animal with a spleen nearly 2x the size of the other naïve squirrels, which seems highly abnormal. Thus, conclusions about changes in spleen weight due to TBI are unwarranted. See also Fig 2F, 2G where there is no statistical difference (although the statistical test was not described) but the authors report a difference.

Re: The authors thank the reviewer for expressing this concern. We will remove the figures corresponding to the spleen and kidney measures as they are not significant. We have also removed descriptions of 2F and 2G as there was no statistical differences found between groups (naive, 4.5mo and 9mo post injury).

7. More detail should be provided about housing. There is no assurance that naïve controls were housed for similar duration/time of year as squirrels designated for CHIMERA. This is especially important given the authors desire to link injury to disruption of seasonal/metabolic patterns. Further, it is unclear whether the 'hibernating' and 'nonhibernating' animals in Fig 1C are all naïve squirrels. It is not clear how long before being assigned to injury the squirrels were housed in the vivarium. This is critical to their characterization as 'non hibernating'. It appears the injury was induced at the beginning of the 'hibernation season.' The authors acknowledge that the timing of initiation of torpor (indicating hibernation) varied across animals through the fall and winter, so it is not clear how the authors know whether the squirrels designated for CHIMERA would or would not have hibernated.

Re: The authors thank the reviewer for this clarification. The naive controls were age matched and housed on the same cage rack as the squirrels designated for CHIMERA. They were maintained for the same duration and time of year as the CHIMERA squirrels.

In Figure 1C, all hibernating (cyan) and non-hibernating (gray) animals were naive. As stated in the methods only five animals were subjected to CHIMERA (magenta).

Animals were received in August and, following a brief quarantine period, were introduced into the colony (September 2024). Body weight measurements were performed by veterinary staff as part of routine animal welfare monitoring.

For seasonal hibernation to occur, animals must be physically transferred by veterinary personnel from the standard housing rack (ambient room temperature) to the hibernaculum maintained at 4 °C, which is located in an adjacent room. Although animals may attempt to enter torpor during the fall, they will not hibernate for the season unless transferred to the hibernaculum. Veterinary staff

were instructed not to move any animals used in this study (both naïve and CHIMERA) to the hibernaculum, even if they exhibited signs of attempted torpor. As a result, all study animals remained awake throughout the season.

The injury was induced in the beginning of December to allow sufficient time to complete the study before the lab closure which was scheduled for the end of the fiscal year Sept 30th, 2025. All squirrels used in the study were monitored daily by veterinary staff and did not hibernate for the season.

8. The conclusions about weight changes are complicated by the apparently lack of body weight measurements just prior to injury in the CHIMERA cohort (see Fig 1C). Thus, the large weight change between 1 month prior to injury and the first post-injury measurement in some injured squirrels cannot reliably be attributed to the injury as all or some may have occurred in the one month prior to the injury.

Re: The authors thank the reviewer for the careful revision. The main body weight difference described in the manuscript occurs in May 2025 and is now explicitly denoted in Figure 1C.

9. The statement that prior reports show a single impact is insufficient to generate injury pathology (Results 1st paragraph) is misleading. Many papers have documented pathology with a single impact, while others report no pathology. This is highly dependent on factors such as the type of model, injury location, injury severity, and outcome parameter.

Re: We thank the reviewer for requesting clarification. In the process of establishing this squirrel model we did attempt single impacts of various impact energies (0.5J, 1J, 1.5J) as well as consecutive impacts (within 30s of each other on the same day). None resulted in any measurable pathological changes. This will be made clear in the text.

10. There is no description of the quantification approach used for optic nerve immunostaining (or CD68 immunostaining). The description of quantification of RGCs is incomplete and should specify factors such as the magnification used for imaging, size or number of fields assessed, how these were selected in a unbiased fashion, and method for cell counting. For example, was this object counting within the analysis software? If so, was there an intensity threshold used for cell detection or size inclusion/exclusion criteria?

Re: The authors thank the reviewer for the clarification. The RGCs were quantified using the NIS Elements software (this is described in the methods) but it is now moved into a separate section called Image Quantification of RGCs and Microglia.
The CD68 immunostaining was manually dotted and counted using a script written in image J. This description is now included in the methods section. Images were taken using a 20x objective on a Nikon A1r microscope.

"Image processing was performed using NIS-Elements AR (General Analysis 3, GA3), which enables integration of conventional segmentation with AI-assisted tools to create customizable measurement workflows. After selecting the appropriate fluorescence channel (red) corresponding to RGCs, the Bright Spots function was used to identify and mask circular, high-intensity features. The typical diameter parameter was set to 10.0 µm, contrast was set to 0.01, symmetry was set to detect all objects, and the intensity range was defined as 0–255. The Grow feature, which expands pixels outward from detected objects, was disabled. For visualization, the output was configured to display circular regions outlining the detected RGCs."

"The density of microglial cells (Iba1+Cells) were quantified manually from whole-mount retinas and ON longitudinal sections. 1) For retinas, we extracted 3 retinal areas (dorsal, visual streak and mid-ventral retinas) with the same frame size (0.5x0.5mm). The dorsal frame was obtained from 2-3mm above the horizontal ON head, the visual streak was obtained from the central region of the visual streak, that corresponds aprox. From 1.5-2.5mm below the ON head, and the mid-ventral frame was extracted from aprox. 4-6mm below the ONH. For retinal and ON head position see Xiao et al., 2021 (PMID: 34253851). 2) For ON sections, a single frame (1x0.2mm) was extracted along the ON axis. Then, all images were manually dotted using image editing software (Adobe Photoshop v26.6.1). The manually annotated microglial cells (10pix round dots representing Iba1+cells) were subsequently quantified using an automated algorithm in FIJI (NIH, Xiao et al., 2021, PMID: 34253851). To obtain the density we divided the number of cells by the image frame area: 0.25mm^2 and 0.2mm^2, for retina or ON images respectively. From all retinas we used 3 per group to evaluate the microglia, and we evaluated the density per area and overall (average of the 3 areas). For ONs we evaluated 6 for control and 10 for TBI."

CD68+Microglial cells were reported qualitatively and not quantified because most of the ON sections evaluated did not present an overall activation, despite we clearly observed activation in branches in some of the ONs. We did not examine all On sections because the squirrel ONs are thicker than mouse or rat´s ON, and there is a chance we missed the branch with degeneration and microglial activation. Thus, we preferred to report qualitatively (instead of quantitative) the presence of Microglia activation as shown in figure 4.

11. Exclusion of a data point for the analysis for data in Figure 3D is not appropriate. The groups are too small to robustly detect 'outliers' and this data point is well within the experimental range across the controls and the injured animals at different time points.

Re: We thank the reviewer for this point. The data was analyzed with PRISM however we will manually turn off outlier detection and include this point in the analysis. The new figure is provided with the new statistical analysis.

12. Speculation about early changes in GFAP expression in optic nerves should be removed from the methods.

Re: We thank the reviewer for making this point, we will remove the speculation regarding GFAP from the Methods.

13. It is unclear what is meant by 'collateral damage' on p. 16

Re: The authors thank the reviewer for their careful revision. This statement can be removed. During initial testing of CHIMERA and the advanced blast simulator (blast wave), body shielding was required to protect the animal from systemic blast exposure. However, these data have been removed from the current manuscript, which focuses exclusively on CHIMERA. Therefore, references to collateral damage from blast wave exposure are not relevant to the present study.

14. There is no mention of anesthesia of the squirrels prior to cardiac perfusion (p. 16). Is this an omission?

Re: The authors thank the reviewer for the clarifying question. Euthanasia was performed in accordance with NEI ACUC Guidelines. Animals were placed in a sealed chamber and exposed to an overdose of isoflurane delivered in 100% oxygen to rapidly induce deep anesthesia and loss of consciousness, followed by respiratory and cardiac arrest. Prior to initiation of secondary cardiac perfusion, death was confirmed by the absence of spontaneous breathing and heart rate, as well as by physical indicators consistent with death, including pallor of the skin of the paws and of the nose, mouth, and tongue. This detail will be added to page 16 of the manuscript.

15. Please indicate in the methods whether animals were returned to isoflurane between the two CHIMERA injuries. Specify whether naïve squirreals received a similar duration exposure to isoflurane as did the CHIMERA cohort.

Re: The authors thank the reviewer for the specification. Yes, animals were briefly returned to isoflurane anesthesia between the two consecutive CHIMERA injuries while they were repositioned. Naïve squirrels were placed in a similar position and received a similar duration of isoflurane exposure as the CHIMERA cohort; however, this exposure for the naive animals occurred at NIH rather than USUHS, and therefore transport of the naïve animals to USUHS was not required. Only the CHIMERA animals were transported to and from USUHS on the same day (round trip). Animals were only housed at the NIH facility. These details will be added to the Methods section.

Reviewer #2 (Remarks to the Author):

The paper introduces a model of TBI (procedural and animal model) that is suitable for comparison with primates. It is generally well written and is sufficient for the aims of the study, although further experimental evidence could have been provided. why not cross-sectional analysis of retina to show changes in layers. It would be good to be added to the result sections.

Re. We thank the reviewer for this valuable suggestion. We agree that retinal cross-sections could provide additional information about potential changes in populations beyond RGCs. However, the CHIMERA model is based on traumatic head impact with accelerated rotational movement, and previous studies have primarily focused on RGCs and their axons, which are the most vulnerable population in the retina under these conditions. Therefore, in this study, we prioritized the evaluation of RGCs as the primary target population. We acknowledge the potential value of assessing other retinal layers and cell types, and we have added a statement in the Discussion highlighting this as a limitation and a possible direction for future studies.

Please further comment in the discussion on the fact that a lot of changes are not significant, can authors think of more repetition for injury to see higher chances of big differences? Or just adding numbers will resolve it

Re. We thank the reviewer for raising this important point. It is true that increasing the number of animals could potentially reduce variability and increase the likelihood of observing statistically significant differences. However, TLGS are a protected species, which limits our ability to substantially increase sample size. In addition, these animals are not bred in our facilities and can only be obtained during a narrow seasonal window—after weaning in late spring but before the onset of torpor in the fall. We also agree that increasing the number of injury repetitions could enhance the magnitude of observable differences. This point has now been added to the Discussion as a consideration for future studies.

Detailed comments below;
Line 53: species name should be in Italics

Re: The authors thank the reviewer for pointing out this inconsistency, and have corrected this in the text.

Line 76: unusual to abbreviate species names in scientific papers, either TLGS as an abbreviation of species name is rather unusual, Anyhow use TLGS or squirrels, consistently in the text not randomly use one of the two

Re: The authors thank the reviewer for pointing out this custom. The text has been corrected to identify the animals by their full species name.

Line 122-124: this statement is not well observed in the figure 1C, better explanation or clarity on what stage these 3 animals are different would be helpful

Re: The authors thank the reviewer for asking for clarification. The text and the figure have been updated: "Strikingly, three of the five squirrels subjected to CHIMERA in early December exhibited extreme fluctuations in increased body weight (numerically annotated) that were uncoordinated with their expected seasonal cycle (May 13, 2025), suggesting a whole-body metabolic disruption induced by TBI (Figure 1C)."

Line 156; show significance on the graph Fig 1I-J

Re: In light of reviewer 3's comment this Figure 1F-L and any corresponding text has been removed.

Line 162: Fig 2 not 2A as ERG is not shown in 2A

Re: The authors thank the reviewer for pointing out this inconsistency, and have corrected the text.

Line 172: CHIMERA???

Re: The authors thank the reviewer for pointing out this inconsistency, and have removed this from the text.

Line 176: retinal ganglion cells and their axons do not contribute to the ERG recording.correct this sentence then

Re: The authors thank the reviewer for pointing out this inconsistency, and have removed mention of retinal ganglion cells from the description.

Fig 2E: please indicate a and b wave on the graph, Fig 2F: why a and b wave were capitalized in the graph but not in the text. they should be the same

Re: The authors thank the reviewer for pointing out this inconsistency. We have amended the graphs to match the text. All references to a-wave and b-wave are now in lower case.

Fig 3C: in C different groups have to be identified

Re: The authors thank the reviewer for the request for clarification. The animal ID numbers and the corresponding left or right eye are now indicated with colors matching each trace. Additionally, the X-axis has been corrected to show Time (months) instead of milliseconds.

Fig 4A: 9 months looks like the image has a different magnification, correct it. Relevant counting method is not described

Re. We thank the reviewer for raising this point. The scale is actually correct; the apparent difference in magnification arises from the eccentricity factor. It is well documented in many species that RGC somas in the retinal periphery are larger, as they occupy more space, while central regions (such as the macula in primates or the visual streak in squirrels) contain smaller, more densely packed RGC somas.
In fact, we have previously documented this difference using Brn3a to label RGC nuclei: nuclei in peripheral regions such as the dorsal retina are larger, whereas those in the visual streak are smaller and more compact. To aid reviewers in visualizing these differences, we have included a figure from our previous study characterizing RGC populations in TLGS (Xiao et al., 2021, PMID: 34253851).

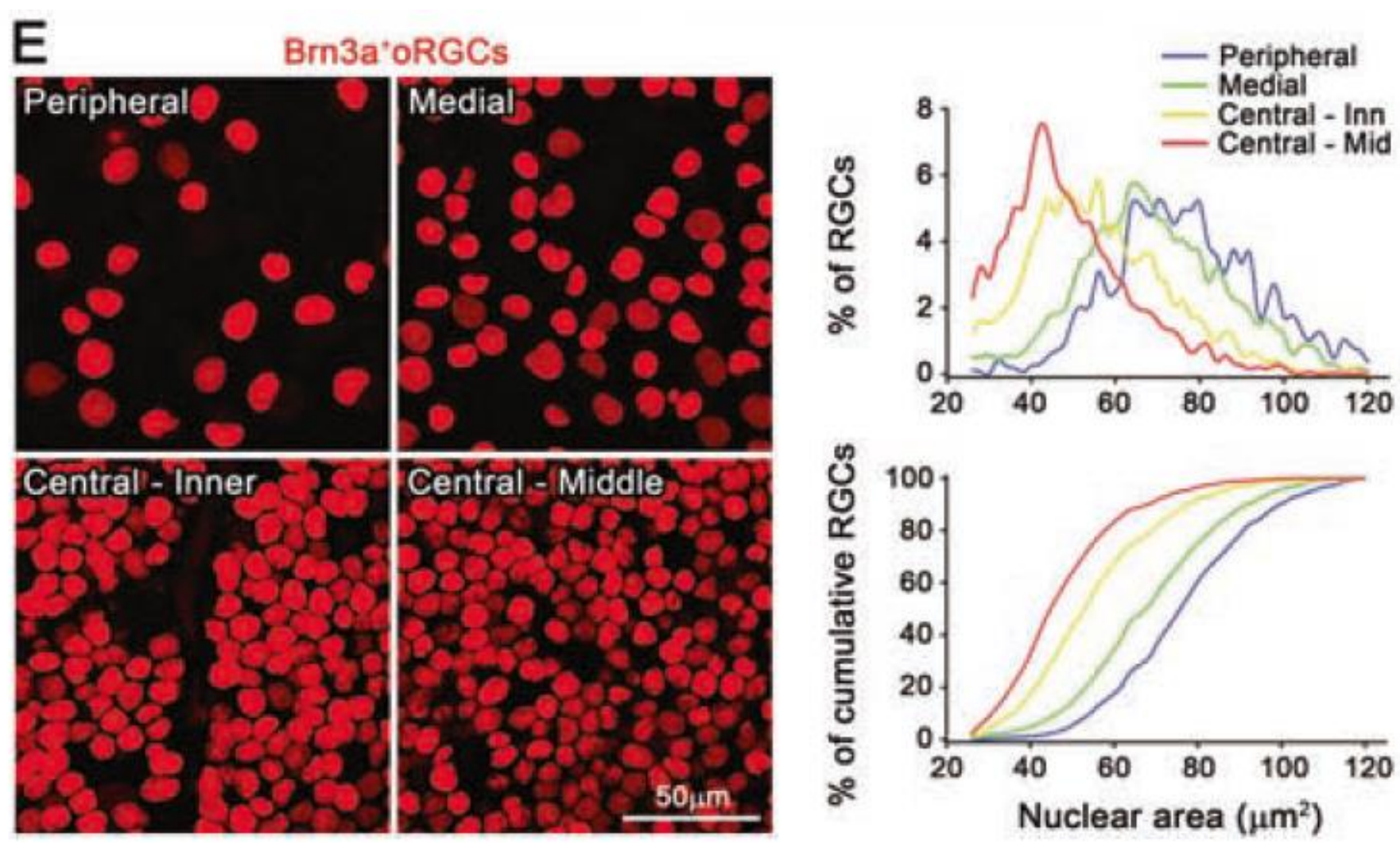

E Different RGC-nuclear areas (µm²) along the retinal surface. Line plots showing the averaged or the cumulative distribution of the Brn3a⁺RGC-nuclei size at four different retinal locations.

The counting method is fully described in the Methods section for clarity on pg. 21.

Line 257-258: ERG does not measure Ganglion cells and their functions, so this statement is not correct

Re: The authors thank the reviewer for pointing out this inconsistency, and have removed mention of retinal ganglion cells from the description.

Line 264: may (repeated word)

Re: The authors thank the reviewer for pointing out this inconsistency, and have removed the repeated word from the text.

Line 266: full stop at the end of sentence

Re: The authors thank the reviewer for pointing out this inconsistency, and have added this to the text.

Line 419: sudden change of font

Re: The authors thank the reviewer for pointing out this inconsistency, and have corrected this in the text.

Re: The authors thank the reviewer for this careful review of our manuscript. We have made all of the above suggested changes.

Reviewer #3 (Remarks to the Author):

This manuscript by Nadal-Nicolas et al. describes work in a useful though "non-traditional" rodent model, the 13-lined ground squirrel (TLGS). This long-lived, diurnal, visually-guided species is arguably a better match for modeling human vision than are short-lived, nocturnal rats/mice. Various published advances in the squirrel's research utility have been made, most recently germline CRISPR-Cas9 gene editing that mimics an inherited retinal degenerative condition. Now, TLGS do hibernate in winter, presenting a physiological background that may or may not affect the species' relevance to the nonhibernating human condition (but also offering intriguing possibilities for physiological resilience). Animal research is always best interpreted through a clear lens of its model species' translational limitations. This manuscript could be a landmark in that it directly addresses this legitimate concern. A series of controlled head impacts provided by the established CHIMERA method were delivered to a small cohort of adolescent TLGS, while an age-matched set of un-injured controls were also employed. Non-invasive data were collected at 3, 6, and 9 mo post-TBI and post-mortem data were collected at 9 mo. Various injuries to retinal ganglion cells and optic nerves were documented using multiple methods linking structure and function. The pathologies detected were not a complete list of those seen in human or traditional rodent models of human, and some measure of repair/recovery appeared 9 mo after injury. This is potentially a very important paper in the Refinement of animal model species that have something to say about the human visual system's response to Traumatic Brain Injury (TBI). However, I observe some shortcomings that must be addressed, followed by re-review.

The most important weakness I perceive is the paper's stated goal of isolating "the metabolic effects of TBI without the potential confounding neuroprotective influence of hibernation" (emphasis mine).

▪ Are TLGS that don't enter torpor (on time or at all) really "healthy subjects" against which a TBI occurs in isolation?

Re: The reviewer raises an interesting and important point. It is true that TLGS are obligatory hibernators and that hibernation is generally recommended; however, our extensive experience demonstrates that preventing hibernation under controlled conditions is not detrimental to animal health.

To enter deep torpor, squirrels reduce their metabolic rate, which allows body temperature to decrease to approximately 25°C. However, animals cannot further reduce their body temperature without an external environmental cue. In natural winter conditions, low ambient temperatures facilitate the progression into deep torpor and full hibernation. In laboratory facilities, this external factor must be provided artificially. If animals are not transferred to a hibernaculum maintained at 4 °C, they fail to enter deep torpor. After several unsuccessful attempts, they cease attempting to hibernate and instead remain in a normal physiological state, with continued access to food and water, a standard light cycle, and apparently normal behavior.
This management approach, together with established veterinary standard operating procedures, allows TLGS to remain awake throughout the winter without any apparent signs of distress or compromised welfare. Importantly, TLGS are a USDA-protected species, and NIH veterinary staff are required to conduct routine, periodic welfare assessments. These necessary interventions can also disrupt rest periods, thereby preventing the onset of torpor or artificially arousing animals that have begun to enter this state. For transparency, the NIH standard operating procedure for the handling of hibernating squirrels is provided.

▪ Could not the demonstrated pre-hibernation changes in TLGS molecular physiology prevent a December TLGS (that has remained euthermic) an imperfect stand-in for a non-hibernating species like the human?

Re: The authors appreciate the point raised by the reviewer. Based on more than 15 years of experience working with TLGS in our facilities, we can state that animals that do not enter torpor remain healthy, and there are no reported or observed health issues correlated with the absence of hibernation. Importantly, we are not aware of any evidence in the literature indicating adverse long-term consequences in TLGS that fail to enter torpor under controlled laboratory conditions.
When TLGS are maintained euthermic during winter, they initially undergo a brief transitional period during which they attempt to enter hibernation. This phase is characterized by a temporary decrease in body temperature, reflecting the expected metabolic shift associated with the onset of torpor. However, if the animals are not provided with the environmental conditions required for deep torpor (i.e., cold ambient temperatures), this attempt is transient. Following this period, body temperature stabilizes and subsequently fluctuates only within a normal circadian range, typically showing minor day–night variations of a few degrees, which are not comparable to the pronounced hypothermia observed during torpor.
Crucially, body temperature is continuously monitored in real time using implanted transponders, allowing us to confirm when animals are no longer attempting to hibernate and have returned to a stable euthermic state. Thus, we can objectively verify that these animals are not in a prolonged pre-hibernation or metabolically compromised condition.
Additionally, because food and water are provided ad libitum throughout the year, TLGS that remain euthermic in December maintain relatively stable body weight and do not exhibit the extreme seasonal weight gain or loss characteristic of squirrels that enter hibernation. Together, these physiological, behavioral, and welfare observations support the conclusion that euthermic TLGS represent healthy subjects and do not display confounding health issues attributable to the absence of torpor.

▪ Don't the following two Discussion points argue AGAINST the Introduction's aim to avoid the hibernation confound?
o (lines 279-80) "TLGS demonstrated relative resilience [to TBI], suggesting species-specific protective mechanisms"
o (lines 314-5) [using TLGS may] "uncover hibernator-specific neuroprotective mechanisms"

Re: The authors appreciate the reviewer's observation regarding this inconsistency and have rephrased the relevant sections accordingly. As noted in the Introduction, this study aimed to eliminate the confounding effects of hibernation by using awake squirrels that did not enter torpor. Nevertheless, as the reviewer correctly highlights, our conclusions must remain cautious. It is currently unclear whether TLGS exhibit relative resilience to TBI—even in the awake state, given that many parameters showed minimal or no significant changes—or whether more pronounced phenotypes could be elicited with additional injury. As now acknowledged in the discussion, future studies incorporating increased injury repetitions may be necessary to fully reveal the extent of TBI-induced effects in this species.

Extending CHIMERA exposure from three consecutive days to five could potentially produce more robust effects. An amendment to the animal protocol to increase the number of CHIMERA days was requested prior to study initiation, but approval by the ACUC was delayed and could not be implemented due to the abrupt notification of the lab's closure. We anticipate that repetitive injury over additional days would yield more discernible phenotypic differences.

Regarding lines 314-5, a study in which hibernating squirrels are subjected to CHIMERA may uncover hibernation-specific neuroprotective mechanisms. This has been rephrased in the text.

▪ Note how this other TLGS paper (https://www.nature.com/articles/s41467-021-23363-x) handled this issue: "One important consideration for this [13LGS] model is the possibility that dramatic seasonal shifts in phenotype with the onset of hibernation may affect metabolic and biochemical parameters of interest, including cerebral hypoxia tolerance. As a result, we have primarily focused on summer tissue collections for 13LGS to avoid any confounding metabolic impacts of hibernation, although we have included some animals collected in winter to bolster sample sizes, which we note in Supplementary Table 6. Any tissues from winter animals included in this dataset were collected during the euthermic interbout arousal period (Supplementary Fig. 11a). We also conducted pilot studies to confirm that seasonal phenotypes did not affect key parameters of interest, such as the abundance and activity of the H2S signal transduction pathway. 13LG squirrels did not show seasonal changes in responses to acute hypoxia breathing, in terms of the levels of sulfide, lactate, and the ratio of NADH/NAD+ in the brain (Supplementary Fig. 11b–d). There were also no seasonal differences in the protein levels of enzymes that metabolize sulfide in the brains of 13LG squirrels (Supplementary Fig. 11e–k), nor differences in H2S pathway enzyme Activities."

Re. We thank the reviewer for raising this important point and providing this manuscript. Indeed, the cited manuscript (Marutani and cols., 2021 (PMID: 34035265)) is consistent with our previous experience; however, there are substantial differences between the experimental paradigms used in that study and in ours.
In Marutani and cols., 2021 (PMID: 34035265), the authors used animals in the interbout arousal (IBA) phase to complete the "awake" group when summer samples were not available. This approach differs fundamentally from our paradigm. Hibernation is not a stable physiological state but a highly dynamic process. During the hibernation season, animals undergo prolonged periods of deep torpor lasting 10–14 days, interspersed with short interbout arousals of approximately 8–12 hours, during which body temperature transiently returns to ~37 °C before the animals re-enter deep torpor again. Importantly, we think animals in IBA remain part of the hibernation cycle and will shortly return to torpor.

In contrast, in our paradigm, animals never entered a true hibernation state: they did not experience deep torpor or interbout arousal phases. Although these animals initially underwent brief, unsuccessful attempts to hibernate, they subsequently ceased these attempts and remained physiologically awake throughout the winter. We therefore consider these animals to be fundamentally different from hibernating animals sampled during IBA. Based on continuous body temperature monitoring, these animals display stable euthermia with only normal circadian fluctuations, supporting the conclusion that they are not engaged in the hibernation program.
While a more comprehensive molecular characterization would be required to definitively demonstrate complete equivalence to summer-euthermic physiology, the Marutani and cols., 2021 (PMID: 34035265) study itself provides supporting evidence that even animals sampled during IBA exhibit physiological parameters comparable to awake conditions. Thus, taken together, these observations support our interpretation that TLGS that did not hibernate during winter are more representative of normal awake conditions than of hibernating states, and that our results are unlikely to be confounded by hibernation-related physiology.
To address this point transparently, we will add a paragraph to the Discussion explicitly acknowledging this limitation and citing the Marutani and cols., 2021 (PMID: 34035265) manuscript as supporting evidence.

▪ Don't get me wrong: TBI in TLGS is a terrific model with much to learn from, and this "isolating" issue should not discount its use. Indeed, the 9 mo post-TBI survival time provides a wealth of longitudinal analysis (though some acute response data would have been nice, too). Just don't claim to do what probably can't be done. The most you can say is that the confound of TORPOR was avoided (and then only if torpor was demonstrably carefully monitored from animals arrival at the lab until the 9 mo post-TBI time point).
The second most important weakness I perceive is that the current version is inconsistent in its comparative strategy of data presentation and discussion. The Introduction makes a good case for the TLGS's applicability to human vision and also cites this species' status as a hibernator, which includes an evolved resilience to the multiple metabolic challenges of torpor-arousal state changes over a calendar year (lines 87-89). The point is then made that, as a TBI model that hibernates, the TLGS may reveal novel therapeutic strategies. I agree, but as usual with TLGS, this puts a burden of proof on the paper to clearly discern where the TLGS model DOES and DOES NOT appear to recapitulate the human condition. This paper's findings would be strengthened greatly by added explicit comparison of TLGS results to both human (the translational "gold ring") and to traditional nocturnal rodent models (to which the paper argues TLGS provide a valuable supplement). Several places, "previous reports" or "prior studies" are cited, but without identifying whether they are based on human or rodent data (e.g. line 259, 263). A new Table in the Discussion comparing species' responses to TBI (headlined by human data, the translational "gold ring") would go far to convince the reader of the TLGS's comparative utility. Admitting where there are "signs of resilience" in TLGS vs. rats/mice might help put the hibernation program running in the background into helpful context, rather than something we'd like to avoid.
The third most important weakness is that a measure of RGC loss from the vital central retina (visual streak in TLGS, fovea in human) is apparently impossible to detect with certain methods (mentioned in lines 300-302), limiting some methods for detecting RGC loss to peripheral retina instead. Yet central retina is the area where documented damage would presumably correlate with the loss of acuity found in the paper's OMR data. A revised Discussion might clarify points like this and, alongside the new Table suggested above, help the reader to know which measures of RGC loss are less equivocal than others.

Re. This is the first study applying this TBI model in the TLGS. Because of the species' distinct anatomy and its classification as a protected species, the experimental design required careful optimization to minimize animal numbers and experimental attempts while still allowing us to determine whether TBI induces measurable alterations in the visual pathway. These anatomical constraints, especially skull thickness, necessitated cautious calibration of the CHIMERA paradigm to achieve reproducible injury without compromising animal welfare.

Regarding the reviewer's second point, we agree that a comparative table summarizing TBI responses across species could be informative. While it is certainly feasible to revise the Discussion to more clearly indicate which prior studies are based on human versus rodent data and to emphasize findings most relevant to human pathology (the translational "gold ring"), we believe that a comprehensive cross-species comparison table would be better suited to a dedicated retrospective or review article rather than a first mechanistic study introducing this model in TLGS. Nevertheless, we will revise the text to clarify species-specific references where appropriate.

With respect to the third point, we note that TLGS do not possess a fovea or a true fovea-like structure. Instead, they exhibit a visual streak, which more closely resembles the human macula than the fovea itself. We agree with the reviewer that the visual streak is primarily responsible for high-acuity vision in TLGS, and therefore the reduction in visual acuity detected by the qOMR test can reasonably be attributed to dysfunction affecting this region.

In this study, RGCs were identified using RBPMS, a widely accepted pan-RGC marker that labels RGC somata. However, in the TLGS visual streak and ganglion cell layer, RGCs are arranged in multiple densely packed rows. When confocal z-stacks are collapsed into two-dimensional images for analysis, individual RGC somata frequently overlap or contact each other, preventing reliable and unbiased quantification in these high-density regions.

Previously, we successfully quantified the distribution and density of RGCs across the entire TLGS retina using the nuclear pan-RGC marker Brn3a (Xiao et al., 2021; PMID: 34253851). Because Brn3a labels RGC nuclei, it allows more accurate automated quantification in 2D images, even within the visual streak. Unfortunately, the antibody used in that study has since been discontinued, and currently available alternatives do not provide comparable labeling quality. As a result, in the present study we relied on RBPMS, which is a robust and reliable RGC marker but is less suitable for quantitative analyses in regions of high cellular density such as the visual streak. Consequently, RGC quantification in this region would not be sufficiently reliable.

Given that CHIMERA-induced injury is expected to primarily affect the visual pathway at the level of RGC axons (optic nerve, chiasm, or optic tracts), we reasoned that axonal damage would impact subsets of axon bundles and, by extension, corresponding retinal regions. The TLGS optic nerve head is elongated horizontally and almost divides the retina into dorsal and ventral regions. Therefore, although we attempt quantification within the visual streak, we end evaluated RGCs in both dorsal and ventral retinal regions to detect whether degeneration was occurring at all, and whether it exhibited regional differences.

We agree with the reviewer that direct assessment of the visual streak would be ideal. However, given current technical limitations, we cannot confidently quantify RGC loss in this region. Nevertheless, the observed reduction in visual acuity measured by qOMR strongly suggests that the visual streak is functionally affected. We will revise the Discussion to explicitly acknowledge this limitation and to clarify the rationale behind our regional analysis strategy.

More specific revision suggestions:

- A revision is needed to remove redundancies such as:

o TLGS being defined twice (lines 528-9)

Re: The authors thank the reviewer for pointing out this inconsistency. In light of reviewer #2's comments, we have removed all usage of "TLGS" in favor of "thirteen-lined ground squirrel."

o Identical sentences about anesthesia in most Methods sections repeating verbatim.
▪ After anesthesia is detailed the first time, simply say "Anesthetized animals were…"

Re: The authors appreciate the reviewer's observation regarding this inconsistency. In light of the journal's comments on descriptions of anesthesia ("as different anaesthesia protocols were used for different applications, we do not consider removing repetitions in your Methods necessary if they allow for accuracy"), we have decided to keep the descriptions as is to maintain accuracy.

▪ Re:anesthesia, the reader needs to be told if Tbody was supported during anesthesia at all times. If not, could a drop in Tbody have triggered some "hibernation" physiological responses in the TLGS, especially considering the different times of year that data were apparently collected (December TBI then data collection at March 3 mo, June 6 mo, September 9 mo)?

Re: The authors appreciate this important question. Body temperature in awake TLGS does not decrease to hibernation-associated levels (e.g., ~4 °C) during anesthesia. Moreover, the anesthesia and CHIMERA procedure are very brief, lasting approximately 3–5 minutes in total, with the majority of this time spent in the induction chamber. As a result, body temperature does not fluctuate substantially during the procedure.
Importantly, during recovery from anesthesia, animals are placed on a heating pad maintained by circulating warm water at approximately 37 °C until they are fully awake. Thus, body temperature is actively supported during the post-anesthetic period. Given the short duration of anesthesia, the limited opportunity for body temperature reduction, and the active thermal support during recovery, it is highly unlikely that anesthesia induced any hibernation-like physiological responses in these animals.

• A revision is needed to correct punctuation (e.g. line 84 has a misplaced comma and line 326 needs a comma after "behavior"), correct misspellings (e.g. line 530 "Trauamtic"), complete any incomplete sentences (e.g. lines 447, 472-4), missing information (e.g. line 445 "(concentration)", and provide missing information (e.g. the instrument used to collect ICGA images is never identified).

Re: The authors thank the reviewer for pointing out these inconsistencies, and have corrected these in the text.

o Sometimes, information that is perhaps better placed (or repeated in) Introduction, Methods, or Discussion is found in Results. That may be a requirement of journal formatting, but should be used with caution. A couple of examples:
▪ Results lines 113-17 might work better in Introduction, so that the reader understands going in why obesity is a consideration having some relationship to optic neuropathy.

Re: The authors thank the reviewer for pointing this out, and have moved these sentences to the introduction.

▪ Results lines 96-101 might work better in Methods, as they cite references (from other species apparently) in which a single impact wasn't sufficient to the paper's purpose. The reader is left wondering what species those were (presumably nonhibernators?). It's then not clear how a series of 6 impacts was settled on for TLGS, nor how that series of 6 relates to what is required for a criterion response in the others species referenced.

As noted in another response below and now clarified in the manuscript, single CHIMERA impacts (0.5 J, 1 J, 1.5 J) were initially tested in thirteen-lined ground squirrels but did not produce measurable changes in RGC number. Consecutive impacts over multiple days were then attempted, which similarly did not yield detectable changes one month post-injury. In the current study, the number of consecutive injury days was increased to three, at which point some changes were observed. The fact that 100% of animals survived this protocol suggests that additional days of injury could potentially produce more robust phenotypic effects.

• Inclusion criteria for the study's TLGS are a basic element of experimental design.
Re. We thank the reviewer for pointing this out. We have now explicitly included the inclusion criteria in the Materials and Methods section, under the Animals subsection. Specifically, we included adult TLGS within their first year of age that were apparently healthy, as assessed by normal body weight, active and alert behavior, normal coat coloration, and absence of hair loss or other visible signs of illness. In addition, animals were selected to achieve a balanced representation of sexes, with approximately equal numbers of male and female TLGS included in the study.

Picking through Results, I find that body weight was monitored in these hibernation-naïve adolescents upon their arrival from the provider in Wisconsin, and five (5) individuals that were not "observed to show signs of torpor" (line 119) were then subjected to CHIMERA in December at the age of 6.5 mo (line 355), i.e. adolescents facing their first-ever winter season.
o How exactly were PRE-CHIMERA "signs of torpor" monitored and their lack determined?

Re: The authors thank the reviewer for requesting clarification. Signs of torpor as described in the SOP include: appearing ataxic, slow to respond or unresponsive, and cold. Torpor related to hibernation is accompanied by a reduction in consumption of food and reduced production of feces and urine. The Animal Care Technicians (ACT), noting torpor-like conditions will scan the body temperature of the animal (s), and immediately complete a Small Animal Clinical Report to notify the Animal Health Technician(s).

o Were signs of torpor monitored POST-CHIMERA?

Yes; however, the animals were maintained on standard housing racks outside of the hibernaculum at ambient room temperature, with ad libitum access to normal food and water. They were not placed on the winter feeding regimen, which would normally involve a gradual reduction in food availability. These conditions promoted continued activity and wakefulness and were not conducive to the induction of hibernation or torpor.

• As obligate hibernators, a circannual rhythm still has a say in TLGS physiology, as they are known to undergo test bouts of torpor in a 70 deg room with a 16:8 L:D photoperiod and daily disturbance of feeding and cleaning. This manuscript aims to "isolate the metabolic effects of TBI without the potential confounding neuroprotective influence of hibernation" (emphasis mine). Might the best

way to do that is to perform TBI in July/August, rather than December? I'm not saying "do your experiments over", I'm saying frame your experiments as performed in a realistic way.

We agree that, in retrospect, greater control over the timing of the experiments would have been ideal. However, approval of the animal protocol by the NIH Animal Care and Use Committee (ACUC) required several years. Even after approval, the ACUC initially authorized only a pilot study (n = 1 per condition) for each CHIMERA impact parameter (0.5 J, 1.0 J, 1.5 J, and 2.0 J). At that time, we were also evaluating the feasibility of using an advanced blast simulator (ABS; 10 ps, 20 psi) to induce injury. The ACUC required that each injury parameter be fully tested and evaluated before proceeding to the next.

Each animal was monitored for approximately one month post-injury prior to retinal collection, resulting in an initial pilot phase that spanned roughly six months. Importantly, this pilot phase involved only a single CHIMERA impact or a single ABS exposure. Subsequent amendments were required to permit repeated injuries in the same animal, again necessitating n = 1 testing for each parameter and a one-month observation period to confirm animal welfare and survival.

Through this iterative process, we ultimately established a protocol consisting of two impacts per day over three consecutive days, which resulted in a 100% survival rate. Given the cumulative delays associated with protocol approval and amendments, and the impending closure of the laboratory in September 2025, the experiments were necessarily initiated in December.

In addition, when we stated that our goal was to "isolate the metabolic effects of TBI without the potential confounding neuroprotective influence of hibernation," we meant to specify that we did not study the TBI paradigm in animals undergoing deep torpor or hibernation to investigate the neuroprotective effects of hibernation itself. As we addressed in earlier responses for the reviewer, the animals used (even during winter) did not enter any hibernation phase and were maintained in euthermic conditions throughout the entire study period. Aside from brief and unsuccessful attempts to enter torpor, the animals remained physiologically awake during the winter.

We agree that due to the timing of the experiment we need to be careful on how we frame the experiments. We have made these changes to the introduction and conclusion.

o Labs that maintain TLGS report a standard process of fall "hibernation trials" (for all ages) in which food & water are removed; the animals are placed in a cold, dark hibernaculum; and, within a couple of days, they are hibernating (e.g. Mousavi et al. 2023 https://pubs.acs.org/doi/10.1021/acschemneuro.3c00268). Would it not have been better to choose subjects that FAILED such fall "hibernation trials", rather than those that were never subjected to trials at all?

Re. We thank the author for bringing this important point up. Importantly, in our animal facilities, all animals are monitored during the year. All animals show signs of torpor at some point during the initial pre-hibernation period. Thus, although we selected the animals for this study (n=5, CHIMERA) months before the winter season, all showed pre-hibernation signs, but these animals were not placed into the hibernaculum preventing hibernation induction.

▪ Alternatively, those that failed to exhibit torpor with or without a trial might be "less healthy" than those that did so. Thoughts?

Re. We thank the reviewer for raising this interesting point. To date, we are not aware of any published evidence indicating that failure to hibernate in a given season induces health issues in TLGS. Our experience shows that animals that do not hibernate in one year are able to hibernate normally in subsequent years without any apparent health consequences. While we acknowledge that TLGS are obligatory hibernators and that hibernation is a natural physiological behavior, our observations suggest that missing a hibernation cycle is not critical for health. Furthermore, routine health assessments conducted by our animal care staff did not reveal any signs of compromised health in animals that failed to enter torpor.

o What is one to make of published work reporting seasonal adjustments to metabolicallyrelevant mRNAs or peptides? What evidence might the manuscript present that would help us know whether or not a euthermic TLGS in December (when hibernation would generally be well underway) is molecularly equivalent to a euthermic TLGS in May (when prolonged euthermia concomitant with reproduction would generally be well underway)?
▪ TLGS: Epperson & Martin, 2002
https://journals.physiology.org/doi/full/10.1152/physiolgenomics.00004.2002
▪ Golden-mantled GS: Williams et al., 2005
https://journals.physiology.org/doi/full/10.1152/physiolgenomics.00301.2004
▪ TLGS: Mousavi et al., 2023
https://pubs.acs.org/doi/10.1021/acschemneuro.3c00268

Re. We thank the reviewer for highlighting these important studies. We fully acknowledge that published work in TLGS and other ground squirrel species (e.g., Epperson & Martin, 2002; Williams et al., 2005; Mousavi et al., 2023) demonstrates seasonal adjustments in metabolically relevant mRNAs, peptides, and other molecular mechanisms. These studies provide valuable insight into the molecular plasticity associated with hibernation and prolonged euthermia <u>*and are now cited accordingly.*</u>
However, we would like to emphasize several points relevant to the present study:

1. ***Focus on anatomical outcomes:*** *Our study specifically examines structural and anatomical changes in the retina and visual pathway, which are fundamentally different from molecular-level changes. While seasonal molecular fluctuations are well documented, our experience with TLGS over many years does not indicate major health issues associated with euthermia during winter; animals remain active, alert, and physiologically normal.*
2. ***Study duration and timing:*** *The current study spans nine months post-TBI. Since the injury was induced in summer, the subsequent nine months encompass sufficient time for degeneration to occur. Any degeneration resulting from the injury should still be detectable in our anatomical analysis. To perform a strictly seasonal molecular comparison (e.g., December vs. May) would have required the animals to enter at least one full hibernation period, which was not compatible with our experimental design. We intentionally maintained the animals in euthermic conditions to isolate the effects of TBI, without the confounding influence of active hibernation.*
3. ***Physiological relevance:*** *Although molecular profiles may fluctuate seasonally, our anatomical and functional outcomes are unlikely to be masked or significantly altered by these molecular adjustments. The animals in December were physiologically awake, and our data indicate that retinal and visual pathway degeneration is measurable and interpretable under these conditions.*

• Because of the possible confound of a circannual rhythm of metabolic adjustment in the absence of expressed torpor, I would strongly suggest adding a Methods timeline graphic ranging from just before CHIMERA to last timepoint of data collection, with a parallel display of calendar month of

data collection, animal age, room temperature and photoperiod, and what type(s) of hibernation monitoring was ongoing:

o Apparently no "naïve"/baseline data collection in December, just before CHIMERA was applied to the TBI group animals
o 3 mo post-TBI data collection at age 9.5 mo (presumably March, when the circannual rhythm would be calling for sexual maturity) (were signs of torpor monitored daily up to this time point? If so, say so)
o 6 mo post-TBI data collection at age 12.5 mo (presumably June, when the circannual rhythm might still be calling for last-chance reproduction) (were signs of torpor monitored daily up to this time point? If so, say so)
o 9 mo post-TBI data collection at age 15.5 mo (presumably September, when the circannual rhythm would be in hibernation preparation mode, and indeed some TLGS this age do exhibit test bouts of torpor beginning in August) (were signs of torpor monitored daily up to this time point? If so, say so)

Re. We thank the reviewer for raising this important and interesting point. Torpor signs in TLGS are typically assessed only in the fall, when animals are naturally preparing for hibernation. Our animal facilities perform routine health checks on all animals throughout the year (twice per week). While these checks do not specifically monitor for torpor outside the fall season, any behavior resembling hibernation during other times of the year would be noted as abnormal and flagged by veterinary staff as a potential health concern. Therefore, although daily torpor monitoring was not performed during non-hibernation periods, any inappropriate torpor-like behavior would have been detected.
We will clarify this in the Methods and include a timeline graphic (Figure 1B) showing experimental interventions, calendar months, animal ages, room temperature, photoperiod, and hibernation-relevant monitoring periods to improve clarity for the reader.

• Starting in Methods at line 337, the manuscript cites an N=5 in the TBI group and an N=4 of in the"Naïve" (never-TBI) group. o Non-invasive data were collected at a time consistent with 3-6-9 mos post-TBI, with "additional control animals" included at 6-9 mos. In contrast, post-mortem data (e.g. IHC) data were collected only at 9 mos, meaning that certain acute TBI-induced changes were missed.

Re. We thank the reviewer for raising this important point. As this is the first study applying the TBI paradigm in a hibernating species, and given the limited availability of TLGS, we prioritized long-term evaluations to capture degenerative changes over a prolonged period. All animals were maintained in active (euthermic) conditions, and tissue collection at 9 months post-TBI allowed us to assess outcomes after sufficient time for degeneration to manifest.
It is also important to note that, based on previous studies in other models, many physiological alterations following TBI—beyond the direct acute injury—often appear well after the initial insult. These long-term changes could correlate with the vision loss observed in military personnel months or even years after injury. Therefore, we focused on long-term changes rather than the acute phase, during which we would not expect substantial RGC degeneration. Early post-mortem analyses could be informative in future studies, but for this first trial, our design maximized the ability to detect meaningful long-term retinal and visual pathway changes.

o When the revision lays out a clear comparative summary of how well TLGS parallels documented human/rat/mouse acute responses to TBI, it should be careful to identify where "no data" were available in this TLGS longitudinal study. Might merit a mention in revised Study Design.

Re. As stated in an earlier response for the reviewer, we agree that a comparative table summarizing TBI responses across species could be informative. While it is certainly feasible to revise the Discussion to more clearly indicate which prior studies are based on human versus rodent data and to emphasize findings most relevant to human pathology (the translational "gold ring"), we believe that a comprehensive cross-species comparison table would be better suited to a dedicated retrospective or review article rather than a first mechanistic study introducing this model in TLGS. Nevertheless, we will revise the text to clarify species-specific references where appropriate.

• I don't find the light dark box data useful because: wild TLGS hide in dark burrows. Indeed, they build short (~24") burrows solely as "escape hatches" that have no connection to the complex home burrow with nest, food cache, and defecatorium chambers. I would predict anxiety to enhance dark bias in TLGS, just as it does in nocturnal rats/mice (lines 305-6). Yet the paper suggests that light bias reflects anxiety in TLGS (lines 309-10). I just see too many alternative explanations to draw that conclusion (listed below), unless/until the appropriate un-injured TLGS controls are tested in exactly the same way across seasons -- and no such controls were used in
this study.
o Could TLGS post-injury light bias be due to damage to the fear centers?
o Could TLGS post-injury light bias be due to acclimation to the procedure?
o Could TLGS post-injury light bias be due to circannual physiological state changes that don't depend on whether torpor itself has occurred, or not? (the 9 mo time point
corresponds to September when the subjects' second hibernation season would be
imminent)

Re: The authors appreciate the reviewer's interpretation and acknowledge that the light–dark box test in the thirteen lined ground squirrel is subject to multiple interpretations, which may limit its utility. We will remove Figure 1 F-L from the manuscript and any corresponding text.

• Immediately post-injury, TLGS were offered acetaminophen-dosed drinking water for 24 hr before and after TBI (line 363). It is not clear if NO-TBI controls also had this intervention. More importantly, the same citation (#47) used to justify this analgesic approach questions its efficacy (on p. 482, #47 says about drinking water acetaminophen: "consistent themes in all of these studies were variability in consumption, both in quantity and time of day that led the authors to
conclude that these methods may be unreliable for provision of consistent and continuous analgesia." For the future studies mentioned, I might recommend subcu injections of Carprofen, 24&48 before and 24&48 days after TBI. We have found this quite effective on TLGS undergoing surgery.

Re. We thank the reviewer for this important point and for the thoughtful suggestion regarding alternative analgesic approaches. In our study, acetaminophen-dosed drinking water was provided 24hr before and up to 24hr after the final TBI exposure based on the recommendations of our collaborators at USUHS who have years of experience with the CHIMERA model in a variety of species and the approval of our veterinary staff. Uninjured control animals did not receive the acetaminophen intervention.

We acknowledge the cited reference's concerns regarding variability in analgesic intake from drinking water. However, as researchers, we are required to follow the analgesia protocols approved by our institutional animal care committee, as well as the Uniformed Services University Health Sciences (USUHS) animal protocol, and any recommendations by the veterinarian staff. Any modification to

the type, timing, or route of analgesia would need to undergo formal review and approval. We would be pleased to consider alternative analgesics, such as subcutaneous Carprofen, in future studies, pending committee approval. However the lab is officially closed and no further studies will be allowed by the institute. For the current study, we strictly adhered to the approved protocol to ensure compliance with all ethical and regulatory guidelines.

My concerns above are not an indictment of the work described in the manuscript under review. Because torpor is just one aspect of the circannual expression of hibernation physiology, I'm not convinced that a 9-month-long study reliant on body mass measurements and the lack of "observed signs of torpor" is truly capable of producing data "without the potential confounding neuroprotective influence of hibernation". The most this study can say, perhaps, is to have removed the potential confound of TORPOR. Reframing the study should reflect this reality.
I encourage the study authors to submit a revised version for re-review. The TLGS model has so much to offer, both in its relevance to the human condition and in its potential for deciphering novel protective strategies. That "double edged sword" is unavoidable, but ALL animal models – and indeed the variability amongst human patients – have their limitations of comparison. Thank you for the opportunity of reviewing this interesting work.

Re. We thank the reviewer for this thoughtful and constructive comment, and we fully agree with the perspective expressed. We acknowledge that torpor is only one aspect of the circannual hibernation physiology and that our 9-month study, based on body mass and the absence of observed torpor, cannot definitively claim to remove all potential hibernation-related influences.
To reflect this more accurately, we will reframe the manuscript to clarify that our study specifically removed the confound of torpor, rather than all aspects of hibernation physiology. We will update the text throughout the manuscript—including the Abstract, Introduction, and Discussion—to ensure this distinction is explicit.
We also appreciate the reviewer's encouragement regarding the translational relevance of the TLGS model and its potential for uncovering novel neuroprotective strategies. We agree that, as with all animal models, there are inherent limitations, and we will highlight these in the revised Discussion to provide appropriate context for our findings.

We hope you will find the referees' comments useful as you decide how to proceed. Should further experimental data or analysis allow you to address these criticisms, we would be happy to look at a substantially revised manuscript. In particular, the reviewers raised concerns if the study was adequately powered. We recommend a power analysis or at the very least discussions on sample size and report effect size for statistics; specify sex distribution on figure legends and provide sex annotated source data, and expand discussion to fully consider the strengths and weaknesses of the proposed model. You must also improve the reporting of methods and experimental design (see link to ARRIVE guidelines below), such as the administration of anaesthesia prior to perfusions (note that as different anaesthesia protocols were used for different applications, we do not consider removing repetitions in your Methods necessary if they allow for accuracy).

We are committed to providing a fair and constructive peer-review process. Do not hesitate to contact us if you wish to discuss the revision or if there are specific requests from the reviewers that you believe are technically impossible or unlikely to yield a meaningful outcome.

If you decide to submit a revised version, we ask that your manuscript complies with our editorial policies. Specifically:

For all graphs depicting a single point value (e.g., mean) with error bars, you must add individual data points or convert the graph to a boxplot or dot-plot to show data distribution.

It's mandatory to provide access to the numerical source data for graphs and charts either through a repository or by providing the data in a Supplementary Data file (in excel format).

All blots/gels must be accompanied by size markers in every figure panel. Uncropped and unedited blot/gel images must be included as Supplementary Figure(s) in the Supplementary Information pdf.

Please ensure that you have complied with the data deposition policies at the Nature Portfolio, please see here.

Please ensure that you have complied with our policies on research involving animals and humans, see here

Please follow the ARRIVE guidelines for reporting animal experiments. Please fully complete an ARRIVE checklist including both the essential and recommended set of items (adding information to the manuscript where needed) and upload this with your revised manuscript.

Please also see our revision checklist for guidance on formatting the manuscript and complying with our policies. A comprehensive guide to our formatting requirements for final submissions is also available for your reference here.

National Institutes of Health
National Eye Institute
Bethesda, Maryland 20892

Title: Impact-Acceleration Head Injury Results in Optic Neuropathy in the Thirteen-Lined Ground Squirrel

We thank the Editors and Reviewers 1, 2, and 3 for their careful, constructive, and insightful evaluation of our manuscript. We are grateful for the time and effort they devoted to a thorough review, and we believe that their comments and suggestions have significantly improved the clarity, rigor, and overall quality of the manuscript. All comments have been addressed point by point in the rebuttal below, and the manuscript has been revised accordingly. We hope that these revisions fully address the Editor's and Reviewers' concerns and meet the objectives of the journal.

Comments:

Editors:

(1) Thank you for including a power analysis in the Methods, though this seems to state that you need n=10 per group, when in reality you have used 5 animals/group? Please clarify this point in the rebuttal (for reviewer benefit) and main text, and in any case please also report (in the Supplementary Information) the statistical comparisons used to justify pooling sexes for analyses.

Re. We thank the Editor for this important point and for the opportunity to clarify our experimental design and sample size. This point was also reported by **Reviewer 1 (point #1).**

In the present study, we analyzed both eyes and optic nerves from each animal for functional and anatomical assays (ERG, PERG, OCT, and ex vivo anatomy). In most of the cases it results in n = 10 experimental samples, derived from 5 biological subjects for the CHIMERA group, and n = 8 samples, derived from 4 biological subjects for the Naïve group; except for the behavioral test (OMR) in which each animal was considered as an sample. We agree that, strictly speaking, this corresponds to 5 biological samples and 10 independent experimental samples (eyes and ONs), and we have revised the Methods and Supplementary Information to explicitly state this distinction for clarity.

Our rationale for considering each eye and ON as an independent experimental unit is based on both the **nature of the injury model** and existing evidence in the literature. CHIMERA induces a diffuse head impact rather than a unilateral ocular or optic nerve insult, which can differentially affect one visual pathway and, consequently, one eye more than the other. Additionally, damage to one visual pathway or eye is not expected to directly induce significant retinal ganglion cell degeneration in the contralateral eye, because:

1. Previous studies in rodents have demonstrated that **axotomy or injury to one optic nerve does not lead to significant contralateral retinal ganglion cell degeneration** in the uninjured eye, supporting the independence of RGC survival between eyes (Nadal-Nicolas et al., 2015 PMID: 26393669; Nadal-Nicolas et al., 2017, 29121969, references now included).
2. While contralateral glial activation (e.g., astroglial or microglial activation) has been reported in certain conditions such as glaucoma (Ramirez et al., 2015, PMID: 26497789), this response does not necessarily translate into contralateral visual impairment or RGC loss (which is the primary anatomical outcome evaluated in this study).

Moreover, we emphasize that any pathological event detected in an eye or ON, whether arising from direct injury or secondary/indirect mechanisms potentially linked to contralateral effects, is **within the scope of this study**. Our goal was not to isolate primary axonal degeneration alone, but rather to determine <u>whether CHIMERA induces **any pathological changes along the visual pathway**, particularly at the level of the optic nerves and retina, that could explain the **functional and behavioral visual deficits** observed after traumatic brain injury in military personnel and civilians</u>. For this reason, our experimental design focused on **later anatomical**

time points, allowing detection of both primary and secondary degenerative processes rather than early, transient changes.

Additionally, we increased the sample size in several assays, such as, the OMR and ERGs. For OMR, the same naïve animals were re-evaluated 9 months later to match age with animals at 9 months post CHIMERA. For ERGs we increased the sample size of the naïve by adding 6 age-matched animals for the 9 months post CHIMERA. It is also worthy to clarify that for OCT measurements, we used the baseline from the same animals measured previously to CHIMERA induction as our ¨control¨ group for comparisons. On the other hand, some anatomical analyses were performed on a subset of the available samples.

Thus, we fully acknowledge that this distinction should be made explicit. Accordingly, we have revised the manuscript to clearly state the sample sizes in the methods, results section and in the figure legends, and we have also clarified this point in discussion section in the transparency, rigor and reproducibility statement. To facilitate trackability of the sample size, we have now re-plotted all graphs to segregate single dot as a sample (animal, retina, or ON) within the corresponding graph.

We have included the following paragraphs, lines:

419-426: *"This approach is justified by the diffuse nature of the CHIMERA head injury, which can differentially affect individual visual pathways, and by prior evidence showing that unilateral ON injury does not induce significant contralateral RGC degeneration (Nadal-Nicolas et al., 2015, 2017). Although contralateral glial activation has been reported in certain conditions (e.g., glaucoma; Ramírez et al., 2015), this does not necessarily result in visual impairment or contralateral RGC loss, which are the primary anatomical outcomes assessed in this study. Accordingly, pathological changes detected in either eye or ON, whether arising from direct or secondary mechanisms, were considered a consequence of the CHIMERA challenge.*
For all groups, both eyes and ONs from each animal were analyzed and treated as independent experimental units (OCT, ERG, PERG, and ex vivo anatomical analysis), except for the behavioral assessment (OMR), where each animal was an experimental unit. In some cases, analyses were performed on a subset of the available samples; accordingly, the specific sample size for each analysis is indicated in the corresponding figure legends. Additionally, no significant sex-dependent differences were detected in the primary functional, behavioral, or anatomical outcome measures. Accordingly, data from males and females were pooled for all analyses to focus on injury-dependent effects and to increase statistical power."

552-554: *"OCT imaging was performed in both eyes (n=10 eyes) from the same animals at three time points: baseline (pre-injury), and 4.5-, and 9-months post-CHIMERA"*

579-580: *"ERG measurements were obtained from both eyes (n=10 eyes) of the same animals at three time points: baseline (pre-injury), and 4.5-, and 9-months post-CHIMERA. Additionally, 5 naïve animals (n=10) were also evaluated (see figure and figures legends for exact sample sizes)."*

622-626: *"OMR measurements were obtained from naïve animals (n=4) and from CHIMERA-injured animals re-evaluated at 3-, 6-, and 9-months post-injury (n=5). Additionally, same naïve animals were re-evaluated 9-months later to match the age of the final post-CHIMERA assessment, thereby excluding aging as contributing factor to changes in visual acuity.*

635-636: *"PERG measurements were obtained from both eyes (n=10 eyes) of the same animals (n=5) at baseline (pre-injury) and 3-, 6-, and 9-months post-CHIMERA."*

656-657: *"Measurements were measured from both eyes for each animal (n = 10 and 8 eyes for naïve and 9-months post-CHIMERA, respectively)."*

725-727: *"RGC numbers were calculated in dorsal (n=5 and 9 for naïve and 9 months post-CHIMERA, respectivelly) and ventral (n=5 for naïve and 9 months post-CHIMERA) retinas with same area (0.64x0.64mm) and divided by 0.4096 mm^2 to obtain their density (cells/mm^2)."*

739-741: *"From all retinas we used a subset (n=4 and 3 for naïve and 9 months post-CHIMERA, respectively) per group to evaluate the microglia, and we evaluated the density per area. For ONs we evaluated n=6 for naïve and n=10 for TBI."*

(2) Related to the above point, please provide sex-annotated source data underlying the main figures as a Supplementary Data file (in spreadsheet format) for reviewer input.

Re. We thank the Editor for this important point and for the opportunity to show the row data, required data has been now included in a spreadsheet format for reviewers' revision (CommsBiol RowData v2.1).

(3) A complete ARRIVE checklist (more details below) and clarification of the ethical approval for the study, and for better reporting of how administration of anesthesia prior to perfusions was conducted (even if some of these details are included in the main text). As before, since different anaesthesia protocols were used for different applications, we do not consider removing repetitions in your Methods necessary if they allow for accuracy.

Re. We thank the Editor for this request and for emphasizing the importance of transparent reporting. A completed ARRIVE checklist has now been included with the revised submission. In addition, we have clarified the ethical approval information and expanded the description of the anesthesia procedures used prior to perfusion, including relevant details for each application. As suggested, we have retained methodological repetitions where necessary to ensure accuracy and clarity.

(4) Per comments from Reviewer #1 about overstating statistical significance in the original Fig 1d and 2f-g, we would encourage you to retain these results (perhaps in the Supplementary Information) but just to ensure they are appropriately discussed (e.g. as non-significant) in the main text.

Re. We appreciate the **Editor's** consideration in retaining data and plots from Fig 1D and Figs 2 F-G. We maintained Fig. 2F-G, and we have now included Fig. 1D in supplementary fig. S1, and we have included paragraphs in the method, results and discussion sections to state that those results are not statistically significant (see line numbers and paragraphs attached below).

However, per **Reviewer #3's comment in Point 22** regarding the usefulness of the light-dark box data, we agree with **Reviewer #3** and have decided to remove all mention of the light-dark box data from the text and figures.

149-156: *"Because these three animals appeared to reach near-maximal weights earlier than anticipated, we assessed spleen and kidneys weight, as enlargement of these organs is commonly associated with obesity-related body composition in humans, and reported spleen size changes after TBI (Ritzel et al., 2018). However, average spleen weights in TBI animals (0.18 g ± 0.05, n=5) were not significantly different (p = 0.35) from those of control animals (0.24 g ± 0.09, n=4; **Supp. Figure S1**). Similarly, kidney weights did not differ appreciably between naïve (0.80 g ± 0.08, n=8, two kidneys per animal) and TBI animals (0.84 g ± 0.14, n=10), with no statistical difference detected (p = 0.44; **Supp. Figure S1**)."*

332-339: *“Interestingly, three of the five CHIMERA-injured animals exhibited abnormally elevated body weight earlier than expected. Because the spleen is of particular interest in the context of traumatic brain injury -where it is known to undergo dynamic changes in size reflecting the activation and mobilization of immune cells (Ritzel et al., 2018)- and because this response has been linked to the so-called “brain–spleen axis,” a bidirectional communication pathway between the nervous and immune systems engaged after brain injury (Wei et al., 2022, Mota & Madden, 2022, Rasouli et al., 2011, Buchmann Godinho et al., 2021), we evaluated spleen and kidney weights. However, no significant differences were detected between CHIMERA-injured and naïve animals, which may reflect the higher resilience or a more localized systemic response to TBI in thirteen-lined ground squirrels compared with other small rodents.”*

686-688: *“The spleen and kidneys were also removed after perfusion. Fat was carefully dissected away from each organ under a dissection microscope, and the wet weight was measured using a laboratory scale.”*

*186-190: “Neither the ERG a-wave nor the b-wave showed significant changes in amplitude (**Figure 2F-G**) or implicit time (**Figure 2H-I**) in CHIMERA-injured animals (n = 10 eyes, 5 animals) compared with age-matched naïve controls at the 4.5-month (n = 8 eyes, 4 animals) and 9- month (n=12 eyes, 6 animals) post-injury time points. Together, these results indicate that CHIMERA injury does not impair outer retinal function.”*

(5) We note in your rebuttal to Reviewer #1 that you have another manuscript in preparation about these squirrel models, presumably in the context of aging. Please provide us with a copy of the manuscript so we can gauge whether it is conceptually distinct from the current study. Depending on the degree of overlap or shared data between studies, we may also ask to share it with the reviewers for their input. Likewise, please include the preliminary data mentioned in Lines 102-104:
"In our preliminary studies (data not shown), a single impact of varying energies (0.5J, 1J, 1.5J) did not generate sufficient injury to produce lasting pathology, nor did consecutive impacts within 30 seconds of each other on the same day"

Re. We appreciate the Editor’s interest in our ongoing work and careful consideration of potential overlap between related studies. That study is an ongoing study in a very early stage of writing. We can confirm it is conceptually and experimentally distinct from the present study. Although both studies involve ground squirrels, they use **different animal cohorts**, address different biological questions, and rely on independent experimental designs and analytical approaches. There will not be overlap in datasets, analyses, or conclusions between the two studies.

For editor and reviewers’ particular knowledge, we share the ID of the animals used on the aging study to prove different animal cohorts:

1-year:	1508 (female)	DOB: 2022
	1509 (female)	
	1530 (male)	
	1547 (female)	
4-5-years:	1205 (female)	DOB: 2018
	1217 (male)	
	1221 (male)	
	1248 (male)	
	1249 (male)	

In addition, preliminary data along with previously published data were presented at 2022 European Association for Vision and Eye Research Festival, 13-15 October 2022, Valencia. Abstract on Special Issue: Acta Ophthalmologica, Volume100, IssueS275. Is the retinal senescent due to neuronal disfunction or neuronal

death? Francisco M. Nadal-Nicolas. First published: 20 December 2022. https://doi.org/10.1111/j.1755-3768.2022.15359. However, we did not report specific results from ground squirrels, but some common features among species with aging.

Thus, this other manuscript is in a **very early stage of development**. A substantial portion of the experimental work and tissue processing is **still ongoing**, and many of the planned analyses, (particularly the anatomical and histological assessments), have not yet been completed. At present, only limited functional and behavioral observations are available, which are not yet supported by corresponding anatomical data. As such, the study is far from finalized and will require extensive additional experimentation, tissue analysis, and validation, and we anticipate that its completion will take well over a year.

For these reasons, we respectfully prefer not to circulate or disclose this unpublished and incomplete work at this stage, as doing so could be misleading and does not accurately reflect the final scope or conclusions of that independent study. We wish to emphasize that the current manuscript is fully self-contained and does not rely on, reuse, or overlap with any data from this ongoing project. It is worth noting that thirteen-lined ground squirrels maintained in captivity can live up to approximately 6 years; therefore, we consider that animals aged 15 months, as included in the present study, are considered young adults rather than aged individuals. Accordingly, we do not expect age-related degeneration to be a contributing factor in the outcomes reported here. So, we have included the following text, also in **Reviewer #1 (in rebuttal as point #0)**

Regarding the preliminary data mentioned in Lines 102–104, these experiments were conducted during early model optimization. Specifically, we evaluated single impacts of varying energies (0.5 J, 1 J, and 1.5 J), as well as consecutive impacts delivered within 30 seconds on the same day. These conditions did not induce consistent or lasting pathological changes and were therefore excluded from the final experimental design. As these exploratory experiments were not powered for hypothesis testing and were not incorporated into the finalized study, they were cited only to justify the selected injury paradigm. We would be happy to provide a concise summary or representative examples of these preliminary observations if the Editor deems it necessary for editorial assessment.

As replied for **reviewer #1 (point #9), and reviewer #3 (point #11)**, we included those exploratory experiments in the material and methods, in CHIMERA induction section, lines:

564-576: *"To establish the CHIMERA model in thirteen-lined ground squirrels, pilot experiments were performed using one animal at a time to assess feasibility, safety, and the induction of measurable pathology. These pilot animals were used exclusively for model optimization, were not included in the final experimental cohorts, and were not reused for subsequent experiments. Single impacts at varying energies (0.5 J, 1 J, and 1.5 J), as well as consecutive impacts delivered within 30 seconds on the same day, were tested. None of these conditions produced detectable pathological changes in the visual pathway probably due to the CHIMERA model, the injury location, the animal model and/or the animal anatomy, such as the scull thickness, despite multiple publications that report damage with one impact (Namjoshi et al., 2017; Sauerbeck et al., 2018; McNamara et al., 2022; Yang et al., 2023; Alkaslasi et al., 2025).*
Throughout model development, animals were closely monitored for unexpected adverse effects, including failure to recover after impact. In such cases, predefined humane endpoints approved by the institutional protocol would have been followed (euthanasia using isoflurane overdose in 100% oxygen, followed by confirmation of death and cervical dislocation)".

(6) Per point 11 from Reviewer #1 (exclusion of an outlier from Fig 3d), and any other relevant figures, please report in the rebuttal letter whether results have changed after an analysis has been repeated/altered.

Re: We thank the Editor for this point. In response to Reviewer #1's comment (point #11), the data were reanalyzed using GraphPad Prism with outlier detection turned off; **no data points were removed or excluded from the analysis**. The updated figure is provided with the revised statistical analysis.

Importantly, the main results and overall conclusions of the study remain unchanged. While the revised analysis resulted in some differences in statistical significance values, these changes do not alter the direction of the effects or the conceptual interpretation of the findings.

We have updated the figure and the corresponding legend to clearly indicate the revised analytical approach for full transparency.

Reviewer #1 (Remarks to the Author):

This submission characterizes changes to the visual system following repeated mild, diffuse TBI induced with the CHIMERA model in the thirteen-lined ground squirrel. Damage to the optic nerve, retina and visual pathway have been established in multiple models of closed head TBI. The authors make the case that because this squirrel species has cone dominated retina, they more closely resemble humans than do rodents (nocturnal animals with rod dominant retina) and would provide a more clinically relevant model. The authors examine visual responses and retinal functional measures as well as histology of the retina and optic nerve. While the premise for developing a model using the ground squirrel is interesting, the study does not contain the necessary controls. This fundamental flaw prohibits drawing meaningful conclusions regarding the effect of repeated mild TBI over a 9-month postinjury period. There are also numerous substantial issues with the experimental approach and rigor, analyses and interpretations. Some of these issues, although not an exhaustive list, are provided below.

0. The controls are stated to be age-matched, but their age is not provided and it is not clear whether they are aged-match at the time of the induction of injury (6.5 months old) or if they are age-matched to the 9 month euthanasia time point for the CHIMERA group (15.5 months old). Further, the control animals do not appear to be monitored over time, to provide age-matched control data to parallel the time course of the injured squirrels (measurements at 3, 4.5, 6 and 9 months after TBI). This is a fundamental flaw in the design. There is no apparent reason why assessments could not have been done repeatedly in the control (naïve) animals. Without parallel measurements in age-matched controls at each time point, the results in the study could merely reflect age-related changes. Changes in the CHIMERA group cannot be attributed to TBI with the current design.

Re: We thank the reviewer for this careful assessment and appreciate the opportunity to clarify the experimental design and control comparisons. This concern has been also clarified for **Editors (please see point #5).**

For behavioral measures such as optomotor response (OMR), control animals consisted of naïve, uninjured squirrels that were age-matched to the experimental animals at the time of CHIMERA induction (6.5 months of age). These same control animals were also assessed longitudinally and measured again 9 months later, corresponding to the 9-month post-injury time point in the CHIMERA group. Because no age-related differences were observed over this relatively short interval, these data were previously pooled. In response to the reviewer’s concern, we have now separated control data by time point and revised the figures and figure legends accordingly to explicitly indicate the age at assessment.

For other outcome measures where only a single comparison was performed (e.g., CHIMERA vs. control at 9 months post-injury), the control animals were age-matched to the CHIMERA group at the terminal time point (15.5 months of age). This was now clarified in the material and methods section, lines:

524-529: *“In some experiments (ERG), additional naïve animals were included at later time point (9 months; n=6 animals, mixed sex; 3 males and 3 females, at ~15 months of age, yielding n = 12 eyes) to minimize the influence of age on baseline measurements. At the conclusion of the 9-month study, the retinas and ONs were collected from the four naïve animals and the five thirteen-lined ground squirrels subjected to repeated CHIMERA injury (**Figure 1B**).”.*

Importantly, we have conducted longitudinal visual function and structural studies in both mice and ground squirrels. We are currently preparing a separate manuscript detailing age-related changes. In that study, squirrels were followed for up to 4-5 years of age. It is worth noting that thirteen-lined ground squirrels maintained in captivity can live up to approximately 6 years; therefore, animals aged 15.5 months, as included in the present study, are considered young adults rather than aged individuals. Accordingly, we do not expect age-related degeneration to be a contributing factor in the outcomes reported here.

Based on these data, the 9-month interval examined here (6.5 to 15.5 months of age) is insufficient to produce measurable age-related changes in visual function, as assessed by PERG amplitudes, ERG amplitudes, and OCT thickness. In our previous analysis, ERG a-wave and b-wave amplitudes did not differ significantly between the 4.5- and 9-month time points; therefore, these data were combined and reported as a single control group. In the current analysis, the data are separated by age, and we find that the previously reported slowing of the ERG responses is no longer significant at 9 months. This correction has been made in the revised manuscript.

The squirrels only breed once a year (within the first two weeks from emerging from hibernation) and are obtained from the University of Wisconsin. We did not obtain any new squirrels this year 2025 due to the freeze in purchases through the Office of Logistics and Acquisition (OLAO) in the federal government.

Finally, ground squirrel availability imposes practical constraints on experimental design. Animals are obtained from the University of Wisconsin and breed only once per year, within a narrow window shortly after emergence from hibernation. Additionally, no new animals were obtained in 2025 due to a federal government-wide freeze on purchases through the Office of Logistics and Acquisition (OLAO). Despite these constraints, the inclusion of age-matched terminal controls, longitudinal assessments of the CHIMERA-injured animals before and after injury, and insights from an ongoing study in aged squirrels (manuscript in a **very early stage of development**) ensure that the observed changes in the CHIMERA group cannot be attributed to aging alone.

Other issues:

1. The study is underpowered, with 4 controls and 5 injured animals.

Re: Thank you for giving us the opportunity to clarify this point. As discussed in detail **in response to Editor (please see point #1)**, the apparent discrepancy arises from the distinction between the number of animals used and the effective sample size for different experimental readouts.

As this is primarily a visual study characterizing the effects of traumatic optic neuropathy in the TLGS, we leveraged data from both eyes. Both eyes are expected to be independently and equivalently affected due to the precise central localization of the piston impact in the CHIMERA device.

We selected (n=4) animals to serve as controls (8 eyes) to be compared to the animals subjected to CHIMERA (n=5) animals (10 eyes). Because squirrels are a USDA-protected species, the NIH Animal Care and Use Committee requested that we limit the number of animals used in this study. To address the reviewer's concerns we have reperformed the statistical analysis using repeated-measures ANOVA where appropriate.

We have also included in discussion, in the transparency, rigor and reproducibility statement the paragraphs in lines 416-432 (and in **Editors, point #1).**

2. The authors should not use the term 'control' or 'naïve' to refer to baseline of injured squirrels These are not equivalent in terms of the design and confuse the interpretation (see for example, Figure 3D where graph is labeled as 'naïve' but legend describes this group as 'pre-injury'). Data are not presented consistently; for example, some outcomes appear to lack the naïve control group entirely (see Figure 2D).

Re: The authors thank the reviewer for asking for clarity. We have gone back through the figures and text to make sure that we are more precise in our descriptions and to not use these terms interchangeably. We will use the terms "baseline" or "pre-injury" to refer to the animals in the CHIMERA condition before being exposed to the CHIMERA paradigm. We will use the term "naïve" to refer to animals in the control condition that are never exposed to CHIMERA.

3. The authors fail to clearly describe what constitutes 'n' for each outcome measure and to report the n's per group for each outcome. While the methods state there are 5 CHIMERA squirrels and 4 naïve controls, the authors refer to some additional controls that are added at later time points. The numbers, sex and ages of these must be specified as well as whether these were used for all outcomes or only selected outcomes. If the latter, a strong rationale should be provided. Oftentimes there are many more data points than 5 in injured groups (see Figures 2, 3, 4) without adequate description of how these larger groups were derived. If multiple measures are taken within a given animal (as in from the two eyes/nerves or multiple measurements within one sample), these cannot be considered as independent samples, and must either be averaged to yield one data point per animal or treated as a repeated measure within an ANOVA design.

Re: The authors thank the reviewer for asking for clarity. This concern was revised in the **response to the Editor (see point #1)** that provide a detailed clarification of this experimental design and in discussion.
We believe the confusion arises from the use of the same animals across different experimental approaches, which results in different effective sample sizes depending on the outcome measured. For behavioral experiments, each animal constituted a single experimental unit (n = 4 for naïve controls, and n = 5 for the TBI group). In contrast, for ERG recordings and anatomical analyses—including retinal OCT thickness, ICG angiography, retinal ganglion cell and microglia quantification, as well as optic nerve assessments (microglia number, microglial activation, and astrocytic intensity)—each eye or optic nerve was treated as an individual experimental unit. This approach was adopted because CHIMERA-induced TBI can differentially affect each visual pathway, and injury to one pathway is not expected to propagate to the contralateral side. However, for ERG we included 6 animals at matched ages of the animals subjected to CHIMERA at 9 months (See the animal list below). And in some cases, analyses were performed on a subset of the available samples; accordingly, the specific sample size for each analysis is now clearly indicated in the corresponding figure legends.

-Definition of n

For all outcome measures, n represents the number of individual animals unless otherwise explicitly stated. Where applicable, bilateral measures were analyzed using repeated-measures ANOVA designs. All statistical analyses have been rechecked and revised to ensure appropriate handling of repeated measures.

-Experimental Groups

-The CHIMERA-injured group consisted of five squirrels:

1799 (female), DOB: 5-20-24
1803 (female), DOB: 5-21-24
1812 (male), DOB: 5-22-24
1813 (male), DOB: 5-22-24
1814 (male), DOB: 5-22-24

-The naïve (uninjured) control group consisted of four squirrels:

1779 (male), DOB: 5-20-24
1827 (male), DOB: 5-23-24
1833 (female), DOB: 5-23-24
1846 (female), DOB: 6-6-24

-The naïve (uninjured) control group (Hibernating (cyan), Figure 1C) consisted of 6 squirrels:

1776 (male), DOB: 5-20-24
1790 (female), DOB: 5-20-24
1804 (female), DOB: 5-21-24
1817 (male), DOB: 5-20-24
1824 (male), DOB: 5-20-24

1845 (female), DOB: 6-4-24

Sex and age at the time of assessment are now explicitly reported in the Methods.

-Age Matching and Cohort Consistency. Ground squirrels hibernate for nearly six months per year and therefore produce only a single litter annually. Mating occurs within a narrow window immediately following emergence from hibernation, and offspring must mature rapidly before the next hibernation period. Consequently, all animals born within a given year—across different mating pairs—are born within an approximately one-month window (typically April through early May). As a result, animals obtained from the same cohort differ in age by no more than one month, ensuring a high degree of age consistency across experimental and control groups.

-Use of Controls Across Outcome Measures. There are no age-dependent changes in OMR, PERG, ERG, or OCT retinal thickness in squirrels between 6.5 months (time of injury) and 15.5 months (terminal collection), based on our preliminary longitudinal aging study in this species (**currently, an ongoing project**). Accordingly, for these functional measures, longitudinal tracking of naïve controls was not required, as each CHIMERA-injured animal served as its own pre-injury baseline control. Statistical analyses for these outcomes have been revised to use repeated-measures ANOVA where appropriate. For outcome measures assessed only at the terminal time point, age-matched naïve controls (15.5 months old) were used for comparison.

-Clarification of Apparent Sample Size Inflation. Figures in which more data points appear than the total number of animals reflect multiple measurements obtained within the same animal (e.g., bilateral eyes). These data points are now clearly identified as within-animal measures and are statistically treated as repeated measures. The analyses no longer treat these data as independent biological replicates, and the figures have been updated to reflect the new statistical analysis.

-Aging Reference Data. As noted above, we are characterizing age-related changes in visual and retinal outcomes in both squirrels and mice for a separate manuscript that will describe interspecies differences. Data confirming the absence of age-dependent effects in squirrels for OMR, PERG, ERG, and OCT retinal thickness over the relevant age range are provided to the reviewer for reference only and are not intended for inclusion in the present manuscript.

-Representative measured values at 12 months of age (naïve squirrels, for reference only):

PERG: 92.29 ± 9.51 µV (amplitude) @ 1 year old.

OCT: 283.22 +/- 4.86 µm (total retinal thickness) @ 1 year old. Measured with a 30 degree lens.

Note: the data in this manuscript was measured using the 55 degree (wide field) lens.

In the current manuscript we now cite Follett et al (2025) that performed a longitudinal study on OCT thickness in thirteen lined ground squirrel earlier this year and concluded that the retinal thickness of elderly animals (5-8 years old) was comparable to younger eyes (12 months old) (see the sentence bellow). And we have also added the sample size in the method section and figure legends (see **Editors, point #1**).

164-165: *"Prior studies have shown that retinal thickness in thirteen lined ground squirrels is unaffected by aging (Follett et al., 2025)."*

Figure-Specific Clarifications

-Figure 2A–C. OCT measurements were obtained from *n* = 5 CHIMERA-injured squirrels at baseline (pre-injury), 4.5 months post-injury, and 9 months post-injury. Measurements were taken from both left and right eyes,

yielding 10 data points per time point. These bilateral measurements were treated as repeated measures within animals.

-Figure 2D–O. Panels depict averaged responses from the 10 eyes at each time point (baseline, 4.5 months post-injury, and 9 months post-injury).

-Figure 3B. Naïve OMR data were obtained from a separate group of age-matched, uninjured squirrels. These animals were assessed at 0 months and again at 9 months to demonstrate the absence of age-related changes in visual acuity. Data from these two time points are now displayed separately to make this distinction explicit.

-Figure 3C. Squirrel identification numbers and left (L) and right (R) eyes are now labeled. The x-axis has been corrected to "Time (months)."

-Figures 3D, 3E, 3G, 3H, and 3I. Baseline measurements are now explicitly labeled as "Pre-injury."

4. The authors state that males and females were used. Given the potential for sex-dependent responses, the number of males and females should be reported. The proportion of males:females should be comparable in control (naïve) and CHIMERA groups and the responses differentiated in the graphs when possible (e.g. different color or shape symbols within the group).

Re: The authors thank the reviewer for requesting clarification. The proportion of males to females was comparable between the CHIMERA-injured and naïve control groups. No sex-dependent differences were observed for any outcome measures; therefore, data from males and females were pooled for all analyses. The number of males and females represented in each group has now been clarified in the Methods, lines:

487-489: *"subjected to CHIMERA (**Figure 1A**) in early December (mixed sex; 3 males and 2 females, ~6.5 months of age at the time of injury), yielding n = 10 eyes and ONs. An additional four non-hibernating age-matched animals served as naïve controls (mixed sex; 2 males and 2 females, ~6.5 months of age), yielding n = 8 eyes and ONs"*

5. The manuscript lacks a statistical analysis methods section. Only in a few cases are statistical approaches detailed (with text in results or figure legend). A comprehensive statistical analysis section should outline the approach used for each outcome measure. Further, statistical methods are at times inconsistent (e.g. the data in Fig 3B and 3D analyzed using different approaches despite identical experimental constructs). Some data are interpreted in the absence of any statistical analysis (e.g. Fig 1I, J). The approaches do not incorporate the use of repeated sampling within an animal and do not consistently incorporate the repeated measures design within the CHIMERA group. For any ANOVA, the F test results, including p value, should be reported.

Re: The authors thank the reviewer for raising this missing section. The suggested changes will be incorporated into the Methods (see below) and figure legends.

751-769: *"Statistical Analysis*
OCT measurements of RNFL and total retinal thickness were analyzed using repeated-measures one-way ANOVA in GraphPad Prism (version 10.5.0), as the dataset included pre-injury baseline measurements and follow-up assessments 4.5- and 9- months post-injury. OMR visual acuity measurements were analyzed using one-way ANOVA, as age-matched naive control animals and CHIMERA-exposed animals were compared. Repeated-measures analysis was not applied because the naïve control animals were not longitudinally matched to the

CHIMERA cohort; control animals were assessed at 0 and 9 months, whereas CHIMERA animals were assessed at 3-, 6-, and -9 months post-injury. Pattern ERG amplitudes and implicit times were analyzed using repeated-measures one-way ANOVA, consistent with the inclusion of pre-injury baseline measurements and follow-up measurements at 4.5- and 9- months post-injury. Retinal vasculature parameters were compared between baseline pre-injury. Retinal vasculature parameters were compared between baseline pre-injury images and 9 months post-CHIMERA injury using Student's t tests. Levene's test was used to compare mean absolute deviations as a measure of variability in indocyanine green (ICG) intensity profiles. Quantification of RGCs and microglia (cells per unit area) was compared between naïve (uninjured) animals and animals at 9 months post-CHIMERA injury using Student's t tests. Glial fibrillary acidic protein (GFAP) intensity in ON images was quantified in ImageJ as integrated density and compared using Student's t tests."

6. A number of datasets are overinterpreted. When a statistical test indicates no difference, a difference should not be reported. For example, there is no difference in spleen weight (Fig 1D) but the authors report a decrease in spleen weight. This supposed difference is driven by one naïve animal with a spleen nearly 2x the size of the other naïve squirrels, which seems highly abnormal. Thus, conclusions about changes in spleen weight due to TBI are unwarranted. See also Fig 2F, 2G where there is no statistical difference (although the statistical test was not described) but the authors report a difference.

Re: The authors thank the reviewer for expressing this concern, and we agree that these insignificant differences should not be reported. As stated in response to the **Editors' Point #4**, we have now included Fig. 1D in supplementary fig. S1, and we have included paragraphs in the method, results, and discussion sections to state that the results in Supp. Figure S1 and Figures 2F-G are not statistically significant, lines:

149-156: *"Because these three animals appeared to reach near-maximal weights earlier than anticipated, we assessed spleen and kidneys weight, as enlargement of these organs is commonly associated with obesity-related body composition in humans, and reported spleen size changes after TBI (Ritzel et al., 2018). However, average spleen weights in TBI animals (0.18 g ± 0.05, n=5) were not significantly different (p = 0.35) from those of control animals (0.24 g ± 0.09, n=4; **Supp. Figure S1**). Similarly, kidney weights did not differ appreciably between naïve (0.80 g ± 0.08, n=8, two kidneys per animal) and TBI animals (0.84 g ± 0.14, n=10), with no statistical difference detected (p = 0.44; **Supp. Figure S1**)."*

332-339: *"Interestingly, three of the five CHIMERA-injured animals exhibited abnormally elevated body weight earlier than expected. Because the spleen is of particular interest in the context of traumatic brain injury—where it is known to undergo dynamic changes in size reflecting the activation and mobilization of immune cells (Ritzel et al., 2018)—and because this response has been linked to the so-called "brain–spleen axis," a bidirectional communication pathway between the nervous and immune systems engaged after brain injury (Wei et al., 2022, Mota & Madden, 2022, Rasouli et al., 2011, Buchmann Godinho et al., 2021), we evaluated spleen and kidney weights. However, no significant differences were detected between CHIMERA-injured and naïve animals, which may reflect the higher resilience or a more localized systemic response to TBI in thirteen-lined ground squirrels compared with other small rodents."*

186-190: *"Neither the ERG a-wave nor the b-wave showed significant changes in amplitude (**Figure 2F-G**) or implicit time (Figure 2H-I) in CHIMERA-injured animals (n = 10 eyes) compared with naïve controls (n = 8 eyes for animals matched to 4.5 months, respectively). Furthermore, when compared with naïve animals matched to the 9-month time point, animals examined at both 4.5- and 9-months post-injury did not exhibit significant alterations in the kinetics of either the a-wave (**Figure 2F,** H) or the b-wave (**Figure 2G,** I). Together, these results indicate that CHIMERA does not impair outer retinal function."*

7. More detail should be provided about housing. There is no assurance that naïve controls were housed for similar duration/time of year as squirrels designated for CHIMERA. This is especially important given the authors desire to link injury to disruption of seasonal/metabolic patterns. Further, it is unclear whether the 'hibernating' and 'nonhibernating' animals in Fig 1C are all naïve squirrels. It is not clear how long before being assigned to injury the squirrels were housed in the vivarium. This is critical to their characterization as 'non hibernating'. It appears the injury was induced at the beginning of the 'hibernation season.' The authors acknowledge that the timing of initiation of torpor (indicating hibernation) varied across animals through the fall and winter, so it is not clear how the authors know whether the squirrels designated for CHIMERA would or would not have hibernated.

Re: The authors thank the reviewer for this clarification. The naive controls were age matched and housed on the same cage rack as the squirrels designated for CHIMERA. They were maintained for the same duration and time of year as the CHIMERA squirrels.

In Figure 1C, all hibernating (cyan) and non-hibernating (gray) animals were naive. As stated in the methods only five animals were subjected to CHIMERA (magenta).

Animals were received in August and, following a brief quarantine period, were introduced into the colony (September 2024). Body weight measurements were performed by veterinary staff as part of routine animal welfare monitoring.

For seasonal hibernation to occur, animals must be physically transferred by veterinary personnel from the standard housing rack (ambient room temperature) to the hibernaculum maintained at 4 °C, which is located in an adjacent room. Although animals may attempt to enter torpor during the fall, they will not hibernate for the season unless transferred to the hibernaculum. Veterinary staff were instructed not to move any animals used in this study (both naïve and CHIMERA) to the hibernaculum, even if they exhibited signs of attempted torpor. As a result, all study animals remained awake throughout the season.

The injury was induced in the beginning of December to allow sufficient time to complete the study before the lab closure which was scheduled for the end of the fiscal year Sept 30th, 2025. All squirrels used in the study were monitored daily by veterinary staff and did not hibernate for the season.

We have added the following paragraphs in methods, lines:

448-460: *"Adult male and female thirteen-lined ground squirrels (Ictidomys tridecemlineatus) were obtained from Dr. Dana Merriman (University of Wisconsin-Oshkosh) and transported to the NIH animal facility. Upon arrival, animals were clinically evaluated by veterinary staff and placed under a quarantine period, after which a second veterinary assessment confirmed health and suitability for experimentation. Animals were then housed individually under standard laboratory conditions with seasonally* adjusted light–dark cycles *designed to replicate* natural photoperiods in Wisconsin*. Food and water were provided ad libitum, with seasonal dietary enrichment supplied in accordance with approved standard operating procedures (SOPs) as previously reported (Merriman et al., 2012). Environmental enrichment, consisting of a red plastic cylinder and a wooden cube, was provided throughout the study to promote exploratory behavior and animal welfare. Throughout the study period, thirteen-lined ground squirrels were maintained under these conditions and monitored twice a week by trained personnel to assess general health and well-being. "*

490-494: *"Inclusion criteria: all animals selected were adults within their first year age that were apparently healthy as assessed by normal body weight, active and alert behavior, normal coat coloration, and absence of hair loss or other visible signs of illness. In addition, animals were selected to achieve a balanced representation of sexes, with approximately equal numbers of male and female animals included in the study."*

501-522: *“Thirteen-lined ground squirrels are obligate hibernators who start hibernating in the fall through the winter, with many documented molecular, genetic and anatomical changes during hibernation (Epperson et al., 2015; Mousavi et al., 2023; Williams et al., 2005; Mrosovsky et al-. 1970; Schartz and Andrews, 2013). During winter, animals often initially undergo a brief transitional period during which they attempt to enter hibernation, a phase characterized by a temporary decrease in body temperature, which reflects the expected metabolic shift associated with the onset of torpor. However, if the animals are not provided with the environmental conditions required for deep torpor (cold ambient temperatures), torpor occurs transiently. Following this period, body temperature stabilizes and subsequently fluctuates only within a normal circadian range, typically showing minor day-night variations of a few degrees, which are not comparable to the pronounced hypothermia observed during torpor. Consequently, in the 9-month period of our experiments, all animals in the study were kept in euthermic conditions (ambient temperature, food, and water) to prevent confounding torpor-related readouts. In fact, it has been described that animals collected at the interbout arousal (IBA), a transitory phase of hibernation where animals wake up and their body temperature increases to euthermic values, resemble thirteen-lined ground squirrels more in active conditions than those in deep torpor (Marutani et al., 2021). Body temperature in the selected animals was continuously monitored in real time using implanted transponders to confirm animals were no longer attempting to hibernate and returned to a stable euthermic state. Thus, we objectively verified that these animals were not in a prolonged pre-hibernation or metabolically compromised condition. In addition, animals that did not undergo a hibernation cycle were evaluated twice per week throughout the study, and no health issues were observed; to date, we are not aware of any evidence indicating that missing a single hibernation cycle adversely affects health in thirteen-lined ground squirrels.”*

8. The conclusions about weight changes are complicated by the apparently lack of body weight measurements just prior to injury in the CHIMERA cohort (see Fig 1C). Thus, the large weight change between 1 month prior to injury and the first post-injury measurement in some injured squirrels cannot reliably be attributed to the injury as all or some may have occurred in the one month prior to the injury.

Re: The authors thank the reviewer for the careful revision. The main body weight difference described in the manuscript occurs in May 2025 and is now explicitly denoted in Figure 1C.

9. The statement that prior reports show a single impact is insufficient to generate injury pathology (Results 1st paragraph) is misleading. Many papers have documented pathology with a single impact, while others report no pathology. This is highly dependent on factors such as the type of model, injury location, injury severity, and outcome parameter.

Re: We thank the reviewer for requesting clarification. In the process of establishing this squirrel model we did attempt single impacts of various impact energies (0.5J, 1J, 1.5J) as well as consecutive impacts (within 30s of each other on the same day). None resulted in any measurable pathological changes. We included the following paragraph to depict our pilot studies, while acknowledging that in other cases degeneration was achieved by single impact (also clarified for **Editors (point #1), and reviewer #3 (point #11), lines**:

564-576: *“To establish the CHIMERA model in thirteen-lined ground squirrels, pilot experiments were performed using one animal at a time to assess feasibility, safety, and the induction of measurable pathology. These pilot animals were used exclusively for model optimization, were not included in the final experimental cohorts, and were not reused for subsequent experiments. Single impacts at varying energies (0.5 J, 1 J, and 1.5 J), as well as consecutive impacts delivered within 30 seconds on the same day, were tested. None of these conditions produced detectable pathological changes in the visual pathway probably due to the CHIMERA model, the injury location, the animal model and/or the animal anatomy, such as the scull thickness, despite multiple publications*

that report damage with one impact (Namjoshi et al., 2017; Sauerbeck et al., 2018; McNamara et al., 2022; Yang et al., 2023; Alkaslasi et al., 2025).
Throughout model development, animals were closely monitored for unexpected adverse effects, including failure to recover after impact. In such cases, predefined humane endpoints approved by the institutional protocol would have been followed (euthanasia using isoflurane overdose in 100% oxygen, followed by confirmation of death and cervical dislocation)".

10. There is no description of the quantification approach used for optic nerve immunostaining (or CD68 immunostaining). The description of quantification of RGCs is incomplete and should specify factors such as the magnification used for imaging, size or number of fields assessed, how these were selected in a unbiased fashion, and method for cell counting. For example, was this object counting within the analysis software? If so, was there an intensity threshold used for cell detection or size inclusion/exclusion criteria?

Re: The authors thank the reviewer for the clarification. The RGCs were quantified using the NIS Elements software. This was described in the methods, but it is now moved into a separate section called Image Quantification of RGCs and Microglia. The Iba1 immunostaining was manually dotted and counted using a script written in image J. This description is now included in the methods section. Images were taken using a 20x objective on a Nikon A1r microscope (now in a new ¨Image acquisition section¨). CD68^{+}Microglial cells were reported qualitatively and not quantified because most of the ON sections evaluated did not present an overall activation, despite we clearly observed activation in branches in some of the ONs. Importantly, we did not examine all ON sections because the squirrel ONs are thicker than mouse or rat´s ON, and there is a chance we missed the branch with degeneration and microglial activation. Thus, we preferred to report qualitatively (instead of quantitative) the presence of Microglia activation as shown in figure 4.

These modifications are now included in the method section, lines:

711-749: *"Image Acquisition.*
Images from whole-mount retinas and entire retinal sections were obtained using a 20x objective on a Nikon A1R microscope. Additionally, images from different retinal areas (dorsal, visual streak, and ventral periphery) were acquired to evaluate regional alterations.

Image Quantification of Retinal ganglion cells and Microglial Cells.
For RGC quantification, image processing was performed using NIS-Elements AR (General Analysis 3, GA3), which enables integration of conventional segmentation with AI-assisted tools to create customizable measurement workflows. After selecting the appropriate fluorescence channel (red) corresponding to RGCs, the Bright Spots function was used to identify and mask circular, high-intensity features. The typical diameter parameter was set to 10.0 µm, contrast was set to 0.01, symmetry was set to detect all objects, and the intensity range was defined as 0–255. The Grow feature, which expands pixels outward from detected objects, was disabled. For visualization, the output was configured to display circular regions outlining the detected RGCs. RGC numbers were calculated in dorsal (n=5 and 9 for naïve and 9 months post-CHIMERA, respectivelly) and ventral (n=5 for naïve and 9 months post-CHIMERA) retinas with same area (0.64x0.64mm) and divided by 0.4096 mm2 to obtain their density (cells/mm2).
The density of microglia cells (Iba+cells) were quantified manually from whole-mount retinas and ON longitudinal sections. For retinas, we extracted 3 retinal areas (dorsal, visual streak, and mid-ventral) with the same frame size (0.5x0.5mm). The dorsal frame was obtained from 2-3mm above the horizontal ON head, the visual streak was obtained from the central region of the visual streak (that corresponds approximately from 1.5-2.5mm below the ON head), and the mid-ventral frame was extracted from approximately 4-6mm below the ON head (for retinal and ON head position, see Xiao et al., 2021). For ON sections, a single frame (1.0x0.2mm) was extracted

along the ON axis. Then, all images were manually dotted using image editing software (Adobe Photoshop v26.6.1). The manually annotated microglia cells (10pix round dots representing Iba+cells) were subsequently quantified using an automated algorithm in FIJI (NIH, Xiao et al., 2021). To obtain the density, we divided the number of cells by the image frame area (0.25mm2 and 0.2mm2 for retina and ON images, respectively). From all retinas we used a subset (n=4 and 3 for naïve and 9 months post-CHIMERA, respectively) per group to evaluate the microglia, and we evaluated the density per area. For ONs we evaluated n=6 for naïve and n=10 for TBI.
Microglial activation in the ON, assessed by CD68 immunoreactivity, was reported qualitatively rather than quantitatively. This decision was based on the observation that most ON sections did not exhibit widespread activation, although focal CD68-positive microglial activation was clearly detected in specific branches in a subset of ONs. Given the relatively large diameter and complex organization of the ON of the thirteen-lined ground squirrel compared with those of mice or rats, not all sections were examined, raising the possibility that localized regions of degeneration and associated microglial activation may have been missed. For these reasons, we elected to report microglial activation qualitatively, as illustrated in Figure 4.

11. Exclusion of a data point for the analysis for data in Figure 3D is not appropriate. The groups are too small to robustly detect 'outliers' and this data point is well within the experimental range across the controls and the injured animals at different time points.

Re: We thank the reviewer for this point. The data was analyzed with PRISM, and we have manually turned off outlier detection and included this point in the analysis. The new figure 3D is provided with updated statistical analysis.

12. Speculation about early changes in GFAP expression in optic nerves should be removed from the methods.

Re: We thank the reviewer for making this point, now a short paragraph is included in discussion, lines:

317-319: *"In addition, although previous publications reporting an increase of GFAP signals after TBI (Zhang et al., 2025; Wang et al., 2025; Nylen et al., 2006), we observed a decrease in GFAP levels in the astrocytes of the ON that may reflect a different stage of the neuroinflammation or axon degeneration."*

13. It is unclear what is meant by 'collateral damage' on p. 16

Re: The authors thank the reviewer for their careful revision. This statement can be removed. During initial testing of CHIMERA and the advanced blast simulator (blast wave), body shielding was required to protect the animal from systemic blast exposure. However, these data have been removed from the current manuscript, which focuses exclusively on CHIMERA. Therefore, references to collateral damage from blast wave exposure are not relevant to the present study.

14. There is no mention of anesthesia of the squirrels prior to cardiac perfusion (p. 16). Is this an omission?

Re: The authors thank the reviewer for the clarifying question. Euthanasia was performed in accordance with NEI ACUC Guidelines. A detailed description for tissue processing is now added in lines:

674-679: *"Tissue processing.*
Prior to cardiac perfusion, Animals were placed in a sealed chamber and exposed to an overdose of isoflurane delivered in 100% oxygen to rapidly induce deep anesthesia and loss of consciousness, followed by respiratory

and cardiac arrest. Prior to initiation of secondary cardiac perfusion, death was confirmed by the absence of spontaneous breathing and heart rate, as well as by physical indicators consistent with death, including pallor of the skin of the paws and of the nose, mouth, and tongue…"

15. Please indicate in the methods whether animals were returned to isoflurane between the two CHIMERA injuries. Specify whether naïve squirreals received a similar duration exposure to isoflurane as did the CHIMERA cohort.

Re: The authors thank the reviewer for the specification. Animals were briefly returned to isoflurane anesthesia between the two consecutive CHIMERA injuries, and naïve squirrels followed similar procedure without receiving any CHIMERA impact. Now detailed in lines:

545-546 *"… during which animals were briefly returned to isoflurane anesthesia while they were repositioned."*

553-558: *"Naïve thirteen-lined ground squirrels (n=4) were placed in a similar position and received a similar duration of isoflurane exposure as the CHIMERA cohort; however, this exposure for the naive animals occurred at NIH rather than USUHS, and therefore transport of the naïve animals to USUHS was not required. Only the CHIMERA animals were transported to and from USUHS on the same day (round trip). Animals were only housed at the NIH facility. Naïve thirteen-lined ground squirrels did not receive acetaminophen in the drinking water."*

Reviewer #2 (Remarks to the Author):

1. The paper introduces a model of TBI (procedural and animal model) that is suitable for comparison with primates. It is generally well written and is sufficient for the aims of the study, although further experimental evidence could have been provided. why not cross-sectional analysis of retina to show changes in layers. It would be good to be added to the result sections.

Re. We thank the reviewer for this valuable suggestion. We agree that retinal cross-sections could provide additional information about potential changes in populations beyond RGCs. However, the CHIMERA model is based on traumatic head impact with accelerated rotational movement, and previous studies have primarily focused on RGCs and their axons, which are the most vulnerable population in the retina under these conditions (RGCs are the only afferent neurons from the retina that projects to the brain). Therefore, in this study, we prioritized the evaluation of RGCs as the primary target population. We have now clarified that in the introduction, lines:

93-106: *"In this study, we sought to characterize visual impairment and alterations in the primary visual pathway induced by the Closed Head Impact Model of Engineered Rotational Acceleration (CHIMERA) model of TBI in thirteen-lined ground squirrels. Because TBI is defined by a concussive head impact and vision originates in the retina before being transmitted to central brain targets, we focused our analyses on key structures along the primary visual pathway. In particular, we examined retinal ganglion cells (RGCs) -the sole afferent neurons of the retina that project to the brain by forming the ONs- as well as the ONs themselves, where axonal injury, neuroinflammatory responses, and glial alterations are known to contribute to visual dysfunction (Orbach et al., 2024; Weber et al., 2008). Concussive events can induce compression, stretching, and dynamic shear forces within ONs, rendering these white matter axons particularly vulnerable even in the absence of direct ocular or ON trauma (Cockerham et al., 2009; Goodrich et al., 2013). By assessing both retinal and optic nerve pathology, we aimed to identify structural correlates of functional and behavioral visual deficits associated with CHIMERA-induced TBI in a diurnal rodent with high visual acuity (Van Hooser and Nelson 2006), and to explore potential mechanisms of neuroprotection relevant to human traumatic brain injury*

We acknowledge the potential value of assessing other retinal layers and cell types, and we have added a statement in the Discussion highlighting this as a limitation and a possible direction for future studies, lines:

363-373: *"We did not evaluate other retinal neuronal populations or perform retinal analyses using cryosections. This decision was based on our primary objective to assess degeneration within the primary visual pathway, where traumatic brain injury is expected to preferentially affect RGCs as a consequence of axonal damage to the ONs. Consistent with this rationale, previous studies have shown that ON axotomy does not result in significant loss of non-RGC neurons within the ganglion cell layer even after prolonged periods up to 15 months (Nadal-Nicolás et al., 2015), nor does it lead to photoreceptor loss up to 6 months post-injury (Ortín-Martínez et al., 2015). Consistent with that our ERG recordings demonstrate no deficits in photoreceptor and bipolar cell function, and therefore the visual impairments observed are mostly due to damage on RGCs and their axons. Nevertheless, we acknowledge that secondary or indirect retinal changes -potentially mediated by glial responses such as microglial or Müller cell activation (Guley et al., 2019)- cannot be fully excluded and warrant further investigation in future studies."*

2. Please further comment in the discussion on the fact that a lot of changes are not significant, can authors think of more repetition for injury to see higher chances of big differences? Or just adding numbers will resolve it

Re. We thank the reviewer for raising this important point. It is true that increasing the number of animals could potentially reduce variability and increase the likelihood of observing statistically significant differences. However, TLGS are a protected species, which limits our ability to substantially increase sample size. In addition, these animals are not bred in our facilities and can only be obtained during a narrow seasonal window—after weaning in late spring but before the onset of torpor in the fall. We also agree that increasing the number of injury repetitions could enhance the magnitude of observable differences. This point has now been added to the Discussion as a consideration for future studies, lines

323-324: *"…suggesting that a greater number of consecutive injury days (e.g. five days) may be required to elicit more robust pathological changes."*

Detailed comments below;
3. Line 53: species name should be in Italics

Re: The authors thank the reviewer for pointing out this inconsistency, and have corrected this in the text.

4. Line 76: unusual to abbreviate species names in scientific papers, either TLGS as an abbreviation of species name is rather unusual, Anyhow use TLGS or squirrels, consistently in the text not randomly use one of the two

Re: The authors thank the reviewer for pointing out this custom. The text has been corrected to identify the animals by their full species name: *"thirteen-lined ground squirrels".*

5. Line 122-124: this statement is not well observed in the figure 1C, better explanation or clarity on what stage these 3 animals are different would be helpful

Re: The authors thank the reviewer for asking for clarification. The text and the figure have been updated (lines):

144-149: *"For body weight comparison we included a group of thirteen-lined ground squirrels that hibernated during the same period for comparison. Strikingly, three of the five thirteen-lined ground squirrels, maintained in euthermic conditions and subjected to CHIMERA in early December exhibited extreme fluctuations in increased body weight (numerically annotated) that were uncoordinated with their expected seasonal cycle (May 13, 2025), suggesting a whole-body metabolic disruption induced by TBI (**Figure 1C**).*

6. Line 156; show significance on the graph Fig 1I-J

Re: In light of **Reviewer 3's Point #22,** Figure 1F-L and any corresponding text has been removed.

7. Line 162: Fig 2 not 2A as ERG is not shown in 2A

Re: The authors thank the reviewer for pointing out this inconsistency, and have corrected the text.

8. Line 172: CHIMERA???

Re: The authors thank the reviewer for pointing out this inconsistency, and have removed this from the text.

9. Line 176: retinal ganglion cells and their axons do not contribute to the ERG recording. correct this sentence then

Re: The authors thank the reviewer for pointing out this inconsistency, and have removed mention of retinal ganglion cells from the description.

10. Fig 2E: please indicate a and b wave on the graph, Fig 2F: why a and b wave were capitalized in the graph but not in the text. they should be the same

Re: The authors thank the reviewer for pointing out this inconsistency. We have amended the graphs to match the text. All references to a-wave and b-wave are now in lower case.

11. Fig 3C: in C different groups have to be identified

Re: The authors thank the reviewer for the request for clarification. The animal ID numbers and the corresponding left or right eye are now indicated with colors matching each trace. Additionally, the X-axis has been corrected to show Time (months) instead of milliseconds.

12. Fig 4A: 9 months looks like the image has a different magnification, correct it. Relevant counting method is not described

Re. We thank the reviewer for raising this point. The scale is actually correct; the apparent difference in magnification arises from the eccentricity factor. It is well documented in many species that RGC somas in the retinal periphery are larger, as they occupy more space, while central regions (such as the macula in primates or the visual streak in squirrels) contain smaller, more densely packed RGC somas.

In fact, we have previously documented this difference using Brn3a to label RGC nuclei: nuclei in peripheral regions such as the dorsal retina are larger, whereas those in the visual streak are smaller and more compact. To aid reviewers in visualizing these differences, we have included a figure from our previous study characterizing RGC populations in TLGS (Xiao et al., 2021, PMID: 34253851).

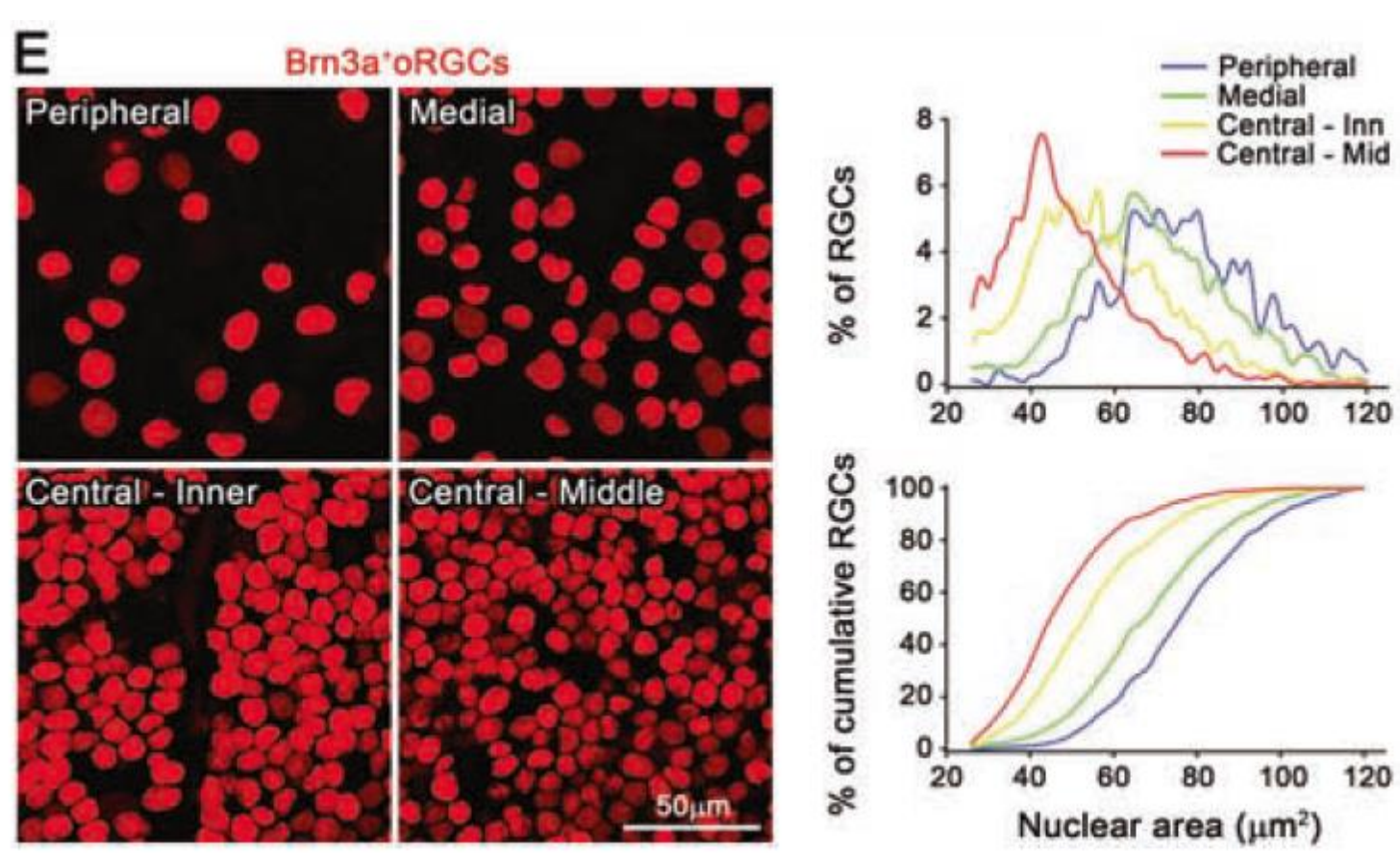

E Different RGC-nuclear areas (µm^2) along the retinal surface. Line plots showing the averaged or the cumulative distribution of the Brn3a$^+$RGC-nuclei size at four different retinal locations.

The counting method is now fully described in the Methods section for clarity (see **Reviewer #1 point #10).**

13. Line 257-258: ERG does not measure Ganglion cells and their functions, so this statement is not correct

Re: The authors thank the reviewer for pointing out this inconsistency, and have removed mention of retinal ganglion cells from the description.

14. Line 264: may (repeated word)

Re: The authors thank the reviewer for pointing out this inconsistency, and have removed the repeated word from the text.

15. Line 266: full stop at the end of sentence

Re: The authors thank the reviewer for pointing out this inconsistency, and have added this to the text.

16. Line 419: sudden change of font

Re: The authors thank the reviewer for pointing out this inconsistency, and have corrected this in the text.

Reviewer #3 (Remarks to the Author):

This manuscript by Nadal-Nicolas et al. describes work in a useful though "non-traditional" rodent model, the 13-lined ground squirrel (TLGS). This long-lived, diurnal, visually-guided species is arguably a better match for modeling human vision than are short-lived, nocturnal rats/mice. Various published advances in the squirrel's research utility have been made, most recently germline CRISPR-Cas9 gene editing that mimics an inherited retinal degenerative condition. Now, TLGS do hibernate in winter, presenting a physiological background that may or may not affect the species' relevance to the nonhibernating human condition (but also offering intriguing possibilities for physiological resilience). Animal research is always best interpreted through a clear lens of its model species' translational limitations. This manuscript could be a landmark in that it directly addresses this legitimate concern. A series of controlled head impacts provided by the established CHIMERA method were delivered to a small cohort of adolescent TLGS, while an age-matched set of un-injured controls were also employed. Non-invasive data were collected at 3, 6, and 9 mo post-TBI and post-mortem data were collected at 9 mo. Various injuries to retinal ganglion cells and optic nerves were documented using multiple methods linking structure and function. The pathologies detected were not a complete list of those seen in human or traditional rodent models of human, and some measure of repair/recovery appeared 9 mo after injury. This is potentially a very important paper in the Refinement of animal model species that have something to say about the human visual system's response to Traumatic Brain Injury (TBI). However, I observe some shortcomings that must be addressed, followed by re-review.
The most important weakness I perceive is the paper's stated goal of isolating "the metabolic effects of TBI without the potential confounding neuroprotective influence of hibernation" (emphasis mine).
(1) ▪ Are TLGS that don't enter torpor (on time or at all) really "healthy subjects" against which a TBI occurs in isolation?

Re: The reviewer raises an interesting and important point. It is true that TLGS are obligatory hibernators and that hibernation is generally recommended; however, our extensive experience demonstrates that preventing hibernation under controlled conditions is not detrimental to animal health.

To enter deep torpor, squirrels reduce their metabolic rate, which allows body temperature to decrease to approximately 25°C. However, animals cannot further reduce their body temperature without an external environmental cue. In natural winter conditions, low ambient temperatures facilitate the progression into deep torpor and full hibernation. In laboratory facilities, this external factor must be provided artificially. If animals are not transferred to a hibernaculum maintained at 4°C, they fail to enter deep torpor. After several unsuccessful attempts, they cease attempting to hibernate and instead remain in a normal physiological state, with continued access to food and water, a standard light cycle, and apparently normal behavior.

This management approach, together with established veterinary standard operating procedures, allows TLGS to remain awake throughout the winter without any apparent signs of distress or compromised welfare. Importantly, TLGS are a USDA-protected species, and NIH veterinary staff are required to conduct routine, periodic welfare assessments. These necessary interventions can also disrupt rest periods, thereby preventing the onset of torpor or artificially arousing animals that have begun to enter this state.

For clarification we have included this paragraph in the methods (study design), lines:

501-519: *"Thirteen-lined ground squirrels are obligate hibernators who start hibernating in the fall through the winter, with many documented molecular, genetic and anatomical changes during hibernation (Epperson et al., 2015; Mousavi et al., 2023; Williams et al., 2005; Mrosovsky et al-. 1970; Schartz and Andrews, 2013). During winter, animals often initially undergo a brief transitional period during which they attempt to enter hibernation, a phase characterized by a temporary decrease in body temperature, which reflects the expected metabolic shift associated with the onset of torpor. However, if the animals are not provided with the environmental conditions*

required for deep torpor (cold ambient temperatures), torpor occurs transiently. Following this period, body temperature stabilizes and subsequently fluctuates only within a normal circadian range, typically showing minor day-night variations of a few degrees, which are not comparable to the pronounced hypothermia observed during torpor. Consequently, in the 9-month period of our experiments, all animals in the study were kept in euthermic conditions (ambient temperature, food, and water) to prevent confounding torpor-related readouts. In fact, it has been described that animals collected at the interbout arousal (IBA), a transitory phase of hibernation where animals wake up and their body temperature increases to euthermic values, resemble thirteen-lined ground squirrels more in active conditions than those in deep torpor (Marutani et al., 2021). Body temperature in the selected animals was continuously monitored in real time using implanted transponders to confirm animals were no longer attempting to hibernate and returned to a stable euthermic state. Thus, we objectively verified that these animals were not in a prolonged pre-hibernation or metabolically compromised condition."

(2) ▪ Could not the demonstrated pre-hibernation changes in TLGS molecular physiology prevent a December TLGS (that has remained euthermic) an imperfect stand-in for a non-hibernating species like the human?

Re: The authors appreciate the point raised by the reviewer. Based on more than 15 years of experience working with TLGS in our facilities, we can state that animals that do not enter torpor remain healthy, and there are no reported or observed health issues correlated with the absence of hibernation. Importantly, we are not aware of any evidence in the literature indicating adverse long-term consequences in TLGS that fail to enter torpor under controlled laboratory conditions.

When TLGS are maintained euthermic during winter, they initially undergo a brief transitional period during which they attempt to enter hibernation. This phase is characterized by a temporary decrease in body temperature, reflecting the expected metabolic shift associated with the onset of torpor. However, if the animals are not provided with the environmental conditions required for deep torpor (i.e., cold ambient temperatures), this attempt is transient. Following this period, body temperature stabilizes and subsequently fluctuates only within a normal circadian range, typically showing minor day–night variations of a few degrees, which are not comparable to the pronounced hypothermia observed during torpor.

Crucially, body temperature is continuously monitored in real time using implanted transponders, allowing us to confirm when animals are no longer attempting to hibernate and have returned to a stable euthermic state. Thus, we can objectively verify that these animals are not in a prolonged pre-hibernation or metabolically compromised condition.

Additionally, because food and water are provided ad libitum throughout the year, TLGS that remain euthermic in December maintain relatively stable body weight and do not exhibit the extreme seasonal weight gain or loss characteristic of squirrels that enter hibernation. Together, these physiological, behavioral, and welfare observations support the conclusion that euthermic TLGS represent healthy subjects and do not display confounding health issues attributable to the absence of torpor.

For clarification we have included this limitation paragraph in the discussion, lines:

374-398: *"One limitation of this study is that we cannot definitively claim to have removed all potential hibernation-related influences, and thus there may be some metabolic impacts of hibernation on the animals in this study. However, hibernation is not a stable physiological state but a highly dynamic process. During the hibernation season, animals undergo prolonged periods of deep torpor lasting 10–14 days, interspersed with short interbout arousals (IBA) of approximately 8–12 hours, during which body temperature transiently returns to ~37 °C before the animals re-enter deep torpor again. Importantly, we think animals in IBA remain part of the hibernation cycle and will shortly return to torpor. In our paradigm, animals never entered a true hibernation state: they did not experience deep torpor or IBA phases. Although these animals initially underwent brief, unsuccessful attempts to hibernate, they subsequently ceased these attempts and remained physiologically*

awake throughout the winter. We therefore consider the animals in our study to be fundamentally different from hibernating animals sampled during IBA. Based on continuous body temperature monitoring, these animals display stable euthermia with only normal circadian fluctuations, supporting the conclusion that they are not engaged in the hibernation program. While a more comprehensive molecular characterization would be required to definitively demonstrate complete equivalence to summer-euthermic physiology, studies have shown that even animals sampled during IBA exhibit physiological parameters comparable to awake conditions (Marutani, et al., 2021). In addition, throughout the experimental period, animals were maintained under seasonally adjusted light–dark cycles and provided ad libitum access to food and water, as previously described (Merriman et al., 2012), but were deliberately not placed in a dark, cold hibernaculum. These housing and husbandry conditions were specifically implemented to prevent the induction of a hibernation cycle. Thus, taken together, these observations support our interpretation that thirteen-lined ground squirrels that did not hibernate during winter are more representative of normal awake conditions than of hibernating states, and that our results are unlikely to be confounded by hibernation-related physiology."

(3) ▪ Don't the following two Discussion points argue AGAINST the Introduction's aim to avoid the hibernation confound?

o (lines 279-80) "TLGS demonstrated relative resilience [to TBI], suggesting species-specific protective mechanisms"

o (lines 314-5) [using TLGS may] "uncover hibernator-specific neuroprotective mechanisms"

Re: The authors appreciate the reviewer's observation regarding this inconsistency and have rephrased the relevant sections accordingly. As noted in the Introduction, this study aimed to eliminate the confounding effects of hibernation by using awake squirrels that did not enter torpor. Nevertheless, as the reviewer correctly highlights, our conclusions must remain cautious. It is currently unclear whether TLGS exhibit relative resilience to TBI—even in the awake state, given that many parameters showed minimal or no significant changes—or whether more pronounced phenotypes could be elicited with additional injury. As now acknowledged in the discussion, future studies incorporating increased injury repetitions may be necessary to fully reveal the extent of TBI-induced effects in this species. Extending CHIMERA exposure from three consecutive days to five could potentially produce more robust effects. An amendment to the animal protocol to increase the number of CHIMERA days was requested prior to study initiation, but approval by the ACUC was delayed and could not be implemented due to the abrupt notification of the lab's closure. We anticipate that repetitive injury over additional days would yield more discernible phenotypic differences.

These thoughts are now included in the discussion, lines

320-324: *"Notably, whereas multiple CHIMERA impacts typically result in more severe degeneration in non-hibernating species such as mice and rats, the squirrel demonstrated relative resilience to 3 consecutive days of 2 CHIMERA impacts, suggesting that a greater number of consecutive injury days (e.g. 5) may be required to elicit more robust pathological changes."*

Regarding the second point (previous lines 314-5), the sentence has been rephrased in discussion, lines:

402-404: *"Furthermore, transcriptomic analyses following acute and chronic head injury in hibernating animals subjected to CHIMERA may uncover hibernator-specific neuroprotective mechanisms that preserve neural and visual function (Li et al., 2018, Ou et al., 2018)."*

(4) ▪ Note how this other TLGS paper (https://www.nature.com/articles/s41467-021-23363-x) handled this issue: "One important consideration for this [13LGS] model is the possibility that dramatic seasonal shifts in phenotype

with the onset of hibernation may affect metabolic and biochemical parameters of interest, including cerebral hypoxia tolerance. As a result, we have primarily focused on summer tissue collections for 13LGS to avoid any confounding metabolic impacts of hibernation, although we have included some animals collected in winter to bolster sample sizes, which we note in Supplementary Table 6. Any tissues from winter animals included in this dataset were collected during the euthermic interbout arousal period (Supplementary Fig. 11a). We also conducted pilot studies to confirm that seasonal phenotypes did not affect key parameters of interest, such as the abundance and activity of the H2S signal transduction pathway. 13LG squirrels did not show seasonal changes in responses to acute hypoxia breathing, in terms of the levels of sulfide, lactate, and the ratio of NADH/NAD+ in the brain (Supplementary Fig. 11b–d). There were also no seasonal differences in the protein levels of enzymes that metabolize sulfide in the brains of 13LG squirrels (Supplementary Fig. 11e–k), nor differences in H2S pathway enzyme
Activities."

Re. We thank the reviewer for raising this important point and providing this manuscript. Indeed, the cited manuscript (Marutani and cols., 2021 (PMID: 34035265)) is consistent with our previous experience; however, there are substantial differences between the experimental paradigms used in that study and in ours.

In Marutani and cols., 2021 (PMID: 34035265), the authors used animals in the interbout arousal (IBA) phase to complete the "awake" group when summer samples were not available. This approach differs fundamentally from our paradigm. Hibernation is not a stable physiological state but a highly dynamic process. During the hibernation season, animals undergo prolonged periods of deep torpor lasting 10–14 days, interspersed with short interbout arousals of approximately 8–12 hours, during which body temperature transiently returns to ~37 °C before the animals re-enter deep torpor again. Importantly, we think animals in IBA remain part of the hibernation cycle and will shortly return to torpor.

In contrast, in our paradigm, animals never entered a true hibernation state: they did not experience deep torpor or interbout arousal phases. Although these animals initially underwent brief, unsuccessful attempts to hibernate, they subsequently ceased these attempts and remained physiologically awake throughout the winter. We therefore consider these animals to be fundamentally different from hibernating animals sampled during IBA. Based on continuous body temperature monitoring, these animals display stable euthermia with only normal circadian fluctuations, supporting the conclusion that they are not engaged in the hibernation program.

While a more comprehensive molecular characterization would be required to definitively demonstrate complete equivalence to summer-euthermic physiology, the Marutani and cols., 2021 (PMID: 34035265) study itself provides supporting evidence that even animals sampled during IBA exhibit physiological parameters comparable to awake conditions. Thus, taken together, these observations support our interpretation that TLGS that did not hibernate during winter are more representative of normal awake conditions than of hibernating states, and that our results are unlikely to be confounded by hibernation-related physiology.

To address this point transparently, we will add a paragraph to the Discussion explicitly acknowledging this limitation and citing the Marutani and cols., 2021 (PMID: 34035265) manuscript as supporting evidence, lines:

374-398: *"One limitation of this study is that we cannot definitively claim to have removed all potential hibernation-related influences, and thus there may be some metabolic impacts of hibernation on the animals in this study. However, hibernation is not a stable physiological state but a highly dynamic process. During the hibernation season, animals undergo prolonged periods of deep torpor lasting 10–14 days, interspersed with short interbout arousals (IBA) of approximately 8–12 hours, during which body temperature transiently returns to ~37 °C before the animals re-enter deep torpor again. Importantly, we think animals in IBA remain part of the hibernation cycle and will shortly return to torpor. In our paradigm, animals never entered a true hibernation state: they did not experience deep torpor or IBA phases. Although these animals initially underwent brief,*

unsuccessful attempts to hibernate, they subsequently ceased these attempts and remained physiologically awake throughout the winter. We therefore consider the animals in our study to be fundamentally different from hibernating animals sampled during IBA. Based on continuous body temperature monitoring, these animals display stable euthermia with only normal circadian fluctuations, supporting the conclusion that they are not engaged in the hibernation program. While a more comprehensive molecular characterization would be required to definitively demonstrate complete equivalence to summer-euthermic physiology, studies have shown that even animals sampled during IBA exhibit physiological parameters comparable to awake conditions (Marutani, et al., 2021 In addition, throughout the experimental period, animals were maintained under seasonally adjusted light–dark cycles and provided ad libitum access to food and water, as previously described (Merriman et al., 2012), but were deliberately not placed in a dark, cold hibernaculum. These housing and husbandry conditions were specifically implemented to prevent the induction of a hibernation cycle. Thus, taken together, these observations support our interpretation that thirteen-lined ground squirrels that did not hibernate during winter are more representative of normal awake conditions than of hibernating states, and that our results are unlikely to be confounded by hibernation-related physiology."

(5) ▪ Don't get me wrong: TBI in TLGS is a terrific model with much to learn from, and this "isolating" issue should not discount its use. Indeed, the 9 mo post-TBI survival time provides a wealth of longitudinal analysis (though some acute response data would have been nice, too). Just don't claim to do what probably can't be done. The most you can say is that the confound of TORPOR was avoided (and then only if torpor was demonstrably carefully monitored from animals arrival at the lab until the 9 mo post-TBI time point).

The second most important weakness I perceive is that the current version is inconsistent in its comparative strategy of data presentation and discussion. The Introduction makes a good case for the TLGS's applicability to human vision and also cites this species' status as a hibernator, which includes an evolved resilience to the multiple metabolic challenges of torpor-arousal state changes over a calendar year (lines 87-89). The point is then made that, as a TBI model that hibernates, the TLGS may reveal novel therapeutic strategies. I agree, but as usual with TLGS, this puts a burden of proof on the paper to clearly discern where the TLGS model DOES and DOES NOT appear to recapitulate the human condition. This paper's findings would be strengthened greatly by added explicit comparison of TLGS results to both human (the translational "gold ring") and to traditional nocturnal rodent models (to which the paper argues TLGS provide a valuable supplement). Several places, "previous reports" or "prior studies" are cited, but without identifying whether they are based on human or rodent data (e.g. line 259, 263). A new Table in the Discussion comparing species' responses to TBI (headlined by human data, the translational "gold ring") would go far to convince the reader of the TLGS's comparative utility. Admitting where there are "signs of resilience" in TLGS vs. rats/mice might help put the hibernation program running in the background into helpful context, rather than something we'd like to avoid.

The third most important weakness is that a measure of RGC loss from the vital central retina (visual streak in TLGS, fovea in human) is apparently impossible to detect with certain methods (mentioned in lines 300-302), limiting some methods for detecting RGC loss to peripheral retina instead. Yet central retina is the area where documented damage would presumably correlate with the loss of acuity found in the paper's OMR data. A revised Discussion might clarify points like this and, alongside the new Table suggested above, help the reader to know which measures of RGC loss are less equivocal than others.

Re. This is the first study applying this TBI model in the TLGS. Because of the species' distinct anatomy and its classification as a protected species, the experimental design required careful optimization to minimize animal numbers and experimental attempts while still allowing us to determine whether TBI induces measurable alterations in the visual pathway. These anatomical constraints, especially skull thickness, necessitated cautious calibration of the CHIMERA paradigm to achieve reproducible injury without compromising animal welfare.

Regarding the reviewer's second point, we agree that a comparative table summarizing TBI responses across species could be informative. While it is certainly feasible to revise the Discussion to more clearly indicate which prior studies are based on human versus rodent data and to emphasize findings most relevant to human pathology (the translational "gold ring"), we believe that a comprehensive cross-species comparison table would be better suited to a dedicated retrospective or review article rather than a first mechanistic study introducing this model in TLGS. Nevertheless, we have carefully revised the text to clarify species-specific references and added numerous new citations along the main text where appropriate.

With respect to the third point, we note that TLGS do not possess a fovea or a true fovea-like structure. Instead, they exhibit a visual streak, which more closely resembles the human macula than the fovea itself (Kriger et al., 1998, PMID: 9682870; Xiao et al., 2021 PMID: 34253851). We agree with the reviewer that the visual streak is primarily responsible for high-acuity vision in TLGS, and therefore the reduction in visual acuity detected by the qOMR test can reasonably be attributed to dysfunction affecting this region.

In this study, RGCs were identified using RBPMS, a widely accepted pan-RGC marker that labels RGC somata. However, in the TLGS visual streak and ganglion cell layer, RGCs are arranged in multiple densely packed rows. When confocal z-stacks are collapsed into two-dimensional images for analysis, individual RGC somata frequently overlap or contact each other, preventing reliable and unbiased quantification in these high-density regions.

Previously, we successfully quantified the distribution and density of RGCs across the entire TLGS retina using the nuclear pan-RGC marker Brn3a (Xiao et al., 2021; PMID: 34253851). Because Brn3a labels RGC nuclei, it allows more accurate automated quantification in 2D images, even within the visual streak. Unfortunately, the antibody used in that study has since been discontinued, and currently available alternatives do not provide comparable labeling quality. As a result, in the present study we relied on RBPMS, which is a robust and reliable RGC marker but is less suitable for quantitative analyses in regions of high cellular density such as the visual streak. Consequently, RGC quantification in this region would not be sufficiently reliable using RBPMS. To support this, we have attached magnified (40x) images from our previous paper comparing RBPMS and Brn3a/DAPI in the visual streak (the higher RGC-density area), note that some areas the RBPMS signal is difficult to distinguish between individualized RGCs (a topic also reviewed in Nadal-Nicolas et al., 2023, PMID: 36594396).

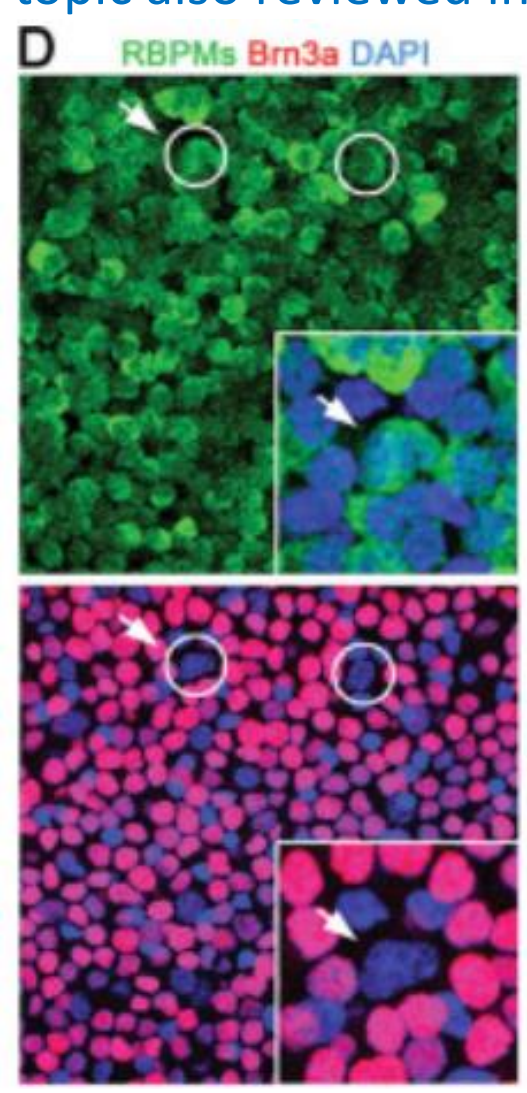

Fig. 3 Characterization of the ganglion cell layer in thirteen-lined ground squirrel flattened retinas…. D. Images of Brn3a and RBPMs co-expressing RGCs in retinal flat-mounts…

Given that CHIMERA-induced injury is expected to primarily affect the visual pathway at the level of RGC axons (optic nerve, chiasm, or optic tracts), we reasoned that axonal damage would impact subsets of axon

bundles and, by extension, corresponding retinal regions. The TLGS optic nerve head is elongated horizontally and almost divides the retina into dorsal and ventral regions. Therefore, although we attempt quantification within the visual streak, we end evaluated RGCs in both dorsal and ventral retinal regions to detect whether degeneration was occurring at all, and whether it exhibited regional differences.

We agree with the reviewer that direct assessment of the visual streak would be ideal. However, given current technical limitations, we cannot confidently quantify RGC loss in this region. Nevertheless, the observed reduction in visual acuity measured by qOMR strongly suggests that the visual streak is functionally affected. We have revised the Discussion to explicitly acknowledge this limitation and to clarify the rationale behind our regional analysis strategy. Lines:

351-360: *"In this study we evaluated RGC loss in dorsal and mid-peripheral ventral retinas because these regions contain fewer RGCs, which are characterized by larger and more sparsely distributed somas that are organized primarily in a monolayer. In contrast, the central retina exhibits a markedly higher RGC density, with an exceptional multilayered arrangement along the visual streak (Xiao et al., 2021; Nadal-Nicolas et al., 2022; Miyagishima et al., 2025Dopamine). This higher baseline density complicates detection of modest RGC loss in retinal flat mounts using RBPMS, as overlapping cytoplasmic labelling in two-dimensional imaging limits accurate cell discrimination. Consequently, this approach is more sensitive to relatively pronounced degeneration within the visual streak (Nadal-Nicolás et al., 2023). Nevertheless, the observed reduction in visual acuity measured by OMR strongly suggests that the visual streak is functionally affected."*

More specific revision suggestions:
• A revision is needed to remove redundancies such as:
(6) o TLGS being defined twice (lines 528-9)

Re: The authors thank the reviewer for pointing out this inconsistency. In light of **reviewer #2 (comment #4)**, we have removed all usage of "TLGS" in favor of "thirteen-lined ground squirrel."

(7) o Identical sentences about anesthesia in most Methods sections repeating verbatim.
▪ After anesthesia is detailed the first time, simply say "Anesthetized animals were…"

Re: The authors appreciate the reviewer's observation regarding this inconsistency. In light of the journal's comments on descriptions of anesthesia ("as different anaesthesia protocols were used for different applications, we do not consider removing repetitions in your Methods necessary if they allow for accuracy"), we have decided to keep the descriptions as is to maintain accuracy.

(8) ▪ Re:anesthesia, the reader needs to be told if Tbody was supported during anesthesia at all times. If not, could a drop in Tbody have triggered some "hibernation" physiological responses in the TLGS, especially considering the different times of year that data were apparently collected (December TBI then data collection at March 3 mo, June 6 mo, September 9 mo)?

Re: The authors appreciate this important question. Body temperature in awake TLGS does not decrease to hibernation-associated levels (e.g., ~4°C) during anesthesia. Moreover, the anesthesia and CHIMERA procedure are very brief, lasting approximately 3–5 minutes in total, with the majority of this time spent in the induction chamber. As a result, body temperature does not fluctuate substantially during the procedure.

Importantly, during recovery from anesthesia, animals are placed on a heating pad maintained by circulating warm water at approximately 37 °C until they are fully awake. Thus, body temperature is actively supported during the post-anesthetic period. Given the short duration of anesthesia, the limited opportunity for

body temperature reduction, and the active thermal support during recovery, it is highly unlikely that anesthesia induced any hibernation-like physiological responses in these animals.

We have accordingly edited our previous details in methods, lines:

540-552: *"Following impact, animals were placed in a clean nesting cage on a warming pad (maintained by circulating warm water at approximately 37°C) and monitored until fully conscious and demonstrating normal mobility before returning to their home cage."*

558-562: *"Both anesthesia and CHIMERA procedures were brief, lasting approximately 3–5 minutes in total. Consequently, body temperature and body weight did not fluctuate substantially during the procedure, making it highly unlikely that the intervention induced any hibernation-like physiological responses in these animals."*

(9) • A revision is needed to correct punctuation (e.g. line 84 has a misplaced comma and line 326 needs a comma after "behavior"), correct misspellings (e.g. line 530 "Trauamtic"), complete any incomplete sentences (e.g. lines 447, 472-4), missing information (e.g. line 445 "(concentration)", and provide missing information (e.g. the instrument used to collect ICGA images is never identified).

Re: The authors thank the reviewer for pointing out these inconsistencies, and have corrected these in the text.

o Sometimes, information that is perhaps better placed (or repeated in) Introduction, Methods, or Discussion is found in Results. That may be a requirement of journal formatting, but should be used with caution. A couple of examples:

(10) ▪ Results lines 113-17 might work better in Introduction, so that the reader understands going in why obesity is a consideration having some relationship to optic neuropathy.

Re: The authors thank the reviewer for pointing this out, and have moved these sentences to the introduction.

(11) ▪ Results lines 96-101 might work better in Methods, as they cite references (from other species apparently) in which a single impact wasn't sufficient to the paper's purpose. The reader is left wondering what species those were (presumably nonhibernators?). It's then not clear how a series of 6 impacts was settled on for TLGS, nor how that series of 6 relates to what is required for a criterion response in the others species referenced.

Re. As noted for **editor (point #5)** and **reviewer #1 (point #9),** we have now clarified in the manuscript that single CHIMERA impacts (0.5 J, 1 J, 1.5 J) were initially tested in thirteen-lined ground squirrels but did not produce measurable changes in RGC number. Consecutive impacts over multiple days were then attempted, which similarly did not yield detectable changes one month post-injury. In the current study, the number of consecutive injury days was increased to three, at which point some changes were observed. The fact that 100% of animals survived this protocol suggests that additional days of injury could potentially produce more robust phenotypic effects. Detailed descriptions were included in lines:

564-576: *"To establish the CHIMERA model in thirteen-lined ground squirrels, pilot experiments were performed using one animal at a time to assess feasibility, safety, and the induction of measurable pathology. These pilot animals were used exclusively for model optimization, were not included in the final experimental cohorts, and were not reused for subsequent experiments. Single impacts at varying energies (0.5 J, 1 J, and 1.5 J), as well as consecutive impacts delivered within 30 seconds on the same day, were tested. None of these conditions produced detectable pathological changes in the visual pathway probably due to the CHIMERA model, the injury location, the animal model and/or the animal anatomy, such as the scull thickness, despite multiple publications*

that report damage with one impact (Namjoshi et al., 2017; Sauerbeck et al., 2018; McNamara et al., 2022; Yang et al., 2023; Alkaslasi et al., 2025).
Throughout model development, animals were closely monitored for unexpected adverse effects, including failure to recover after impact. In such cases, predefined humane endpoints approved by the institutional protocol would have been followed (euthanasia using isoflurane overdose in 100% oxygen, followed by confirmation of death and cervical dislocation)".

(12) • Inclusion criteria for the study's TLGS are a basic element of experimental design.

Re. We thank the reviewer for pointing this out. We have now explicitly included the inclusion criteria in the Materials and Methods section, under the study design subsection. Specifically, we included, lines:

490-494; *"Inclusion criteria: all animals selected adult TLGS within their first year of age that were apparently healthy, as assessed by normal body weight, active and alert behavior, normal coat coloration, and absence of hair loss or other visible signs of illness. In addition, animals were selected to achieve a balanced representation of sexes, with approximately equal numbers of male and female TLGS included in the study."*

(13) Picking through Results, I find that body weight was monitored in these hibernation-naïve adolescents upon their arrival from the provider in Wisconsin, and five (5) individuals that were not "observed to show signs of torpor" (line 119) were then subjected to CHIMERA in December at the age of 6.5 mo (line 355), i.e. adolescents facing their first-ever winter season.
o How exactly were PRE-CHIMERA "signs of torpor" monitored and their lack determined?

Re: The authors thank the reviewer for requesting clarification. Signs of torpor as described in the SOP include: appearing ataxic, slow to respond or unresponsive, and cold. Torpor related to hibernation is accompanied by a reduction in consumption of food and reduced production of feces and urine. The Animal Care Technicians (ACT), noting torpor-like conditions will scan the body temperature of the animal (s), and immediately complete a Small Animal Clinical Report to notify the Animal Health Technician(s).

None of the animals undergoing this study present signs of torpor, as we previously replied in **Reviewer 3, point #8**, lines:

550-552: *"Following impact, animals were placed in a clean nesting cage on a warming pad (maintained by circulating warm water at approximately 37°C) and monitored until fully conscious and demonstrating normal mobility before returning to their home cage."*

558-562: *"Both anesthesia and CHIMERA procedures were brief, lasting approximately 3–5 minutes in total. Consequently, body temperature and body weight did not fluctuate substantially during the procedure, making it highly unlikely that the intervention induced any hibernation-like physiological responses in these animals."*

(14) o Were signs of torpor monitored POST-CHIMERA?

Re: We thank the reviewer for raising this important and interesting point. Torpor signs in TLGS are typically assessed only in the fall, when animals are naturally preparing for hibernation. Our animal facilities perform routine health checks on all animals throughout the year (twice per week). While these checks do not specifically monitor for torpor outside the fall season, any behavior resembling hibernation during other times of the year would be noted as abnormal and flagged by veterinary staff as a potential health concern. Therefore, although

daily torpor monitoring was not performed during non-hibernation periods, any inappropriate torpor-like behavior would have been detected.

Additionally, the animals were maintained on standard housing racks outside of the hibernaculum at ambient room temperature, with ad libitum access to normal food and water. They were not placed on the winter feeding regimen, which would normally involve a gradual reduction in food availability. These conditions promoted continued activity and wakefulness and were not conducive to the induction of hibernation or torpor.

We clarified this in the Methods, in the study design (see below, also in **Reviewer #3, point #19**), in the CHIMERA induction section (see below), and including a timeline graphic (Figure 1B) showing experimental interventions, calendar months, animal ages, room temperature, photoperiod, and hibernation-relevant monitoring periods to improve clarity for the reader.

519-522: *"In addition, animals that did not undergo a hibernation cycle were evaluated twice per week throughout the study, and no health issues were observed; to date, we are not aware of any evidence indicating that missing a single hibernation cycle adversely affects health in thirteen-lined ground squirrels."*

562-563: *"Additionally, animals were maintained at ambient room temperature, with ad libitum access to normal food regimen and water."*

(15) • As obligate hibernators, a circannual rhythm still has a say in TLGS physiology, as they are known to undergo test bouts of torpor in a 70 deg room with a 16:8 L:D photoperiod and daily disturbance of feeding and cleaning. This manuscript aims to "isolate the metabolic effects of TBI without the potential confounding neuroprotective influence of hibernation" (emphasis mine). Might the best way to do that is to perform TBI in July/August, rather than December? I'm not saying "do your experiments over", I'm saying frame your experiments as performed in a realistic way.

Re: We agree that, in retrospect, greater control over the timing of the experiments would have been ideal. However, approval of the animal protocol by the NIH Animal Care and Use Committee (ACUC) required several years. Even after approval, the ACUC initially authorized only a pilot study (n = 1 per condition) for each CHIMERA impact parameter (0.5 J, 1.0 J, 1.5 J, and 2.0 J). At that time, we were also evaluating the feasibility of using an advanced blast simulator (ABS; 10 ps, 20 psi) to induce injury. The ACUC required that each injury parameter be fully tested and evaluated before proceeding to the next.

Each animal was monitored for approximately one-month post-injury prior to retinal collection, resulting in an initial pilot phase that spanned roughly six months. Importantly, this pilot phase involved only a single CHIMERA impact or a single ABS exposure. Subsequent amendments were required to permit repeated injuries in the same animal, again necessitating n = 1 testing for each parameter and a one-month observation period to confirm animal welfare and survival.

Through this iterative process, we ultimately established a protocol consisting of two impacts per day over three consecutive days, which resulted in a 100% survival rate. Given the cumulative delays associated with protocol approval and amendments, and the impending closure of the laboratory in September 2025, the experiments were necessarily initiated in December.

In addition, when we stated that our goal was to "isolate the metabolic effects of TBI without the potential confounding neuroprotective influence of hibernation," we meant to specify that **we did not study the TBI paradigm in animals undergoing deep torpor or hibernation to investigate the neuroprotective effects of hibernation itself**. As we addressed in earlier responses for the reviewer, the animals used (even during winter) did not enter any hibernation phase and were maintained in euthermic conditions throughout the entire study period. Aside from brief and unsuccessful attempts to enter torpor, the animals remained physiologically awake during the winter.

We agree that due to the timing of the experiment we need to be careful on how we frame the experiments. We have made these changes to the study design, lines:

495-500: *"To evaluate visual impairment and degeneration induced by CHIMERA in thirteen-lined ground squirrels under awake conditions (thereby avoiding potential confounding neuroprotective effects of hibernation) and because degenerative changes following traumatic brain injury may be more readily detectable at later stages, we extended the anatomical evaluations to 9 months post-CHIMERA and ensured that thirteen-lined ground squirrels remained predominantly in euthermic conditions throughout the post-injury period."*

(16) o Labs that maintain TLGS report a standard process of fall "hibernation trials" (for all ages) in which food & water are removed; the animals are placed in a cold, dark hibernaculum; and, within a couple of days, they are hibernating (e.g. Mousavi et al. 2023 https://pubs.acs.org/doi/10.1021/acschemneuro.3c00268). Would it not have been better to choose subjects that FAILED such fall "hibernation trials", rather than those that were never subjected to trials at all?

Re. We thank the reviewer for raising this important point. In our animal facility, all squirrels are monitored year-round, and all animals exhibit signs of torpor during the initial pre-hibernation period. Accordingly, although the animals used in this study (n = 5, CHIMERA group) were selected several months before the winter season, they did display early pre-hibernation signs. However, these animals were intentionally **not placed in a hibernaculum**, thereby preventing the induction of full hibernation. In addition, food and water were provided ad libitum, and animals were not exposed to the cold, dark environmental conditions required to trigger hibernation. These measures were deliberately implemented to maintain the animals in a euthermic state and to avoid potential confounding effects of hibernation.

As requested by **reviewer #1, point (#7),** lines:

501-522: *"Thirteen-lined ground squirrels are obligate hibernators who start hibernating in the fall through the winter, with many documented molecular, genetic and anatomical changes during hibernation (Epperson et al., 2015; Mousavi et al., 2023; Williams et al., 2005; Mrosovsky et al-. 1970; Schartz and Andrews, 2013). During winter, animals often initially undergo a brief transitional period during which they attempt to enter hibernation, a phase characterized by a temporary decrease in body temperature, which reflects the expected metabolic shift associated with the onset of torpor. However, if the animals are not provided with the environmental conditions required for deep torpor (cold ambient temperatures), torpor occurs transiently. Following this period, body temperature stabilizes and subsequently fluctuates only within a normal circadian range, typically showing minor day-night variations of a few degrees, which are not comparable to the pronounced hypothermia observed during torpor. Consequently, in the 9-month period of our experiments, all animals in the study were kept in euthermic conditions (ambient temperature, food, and water) to prevent confounding torpor-related readouts. In fact, it has been described that animals collected at the interbout arousal (IBA), a transitory phase of hibernation where animals wake up and their body temperature increases to euthermic values, resemble thirteen-lined ground squirrels more in active conditions than those in deep torpor (Marutani et al., 2021). Body temperature in the selected animals was continuously monitored in real time using implanted transponders to confirm animals were no longer attempting to hibernate and returned to a stable euthermic state. Thus, we objectively verified that these animals were not in a prolonged pre-hibernation or metabolically compromised condition. In addition, animals that did not undergo a hibernation cycle were evaluated twice per week throughout the study, and no health issues were observed; to date, we are not aware of any evidence indicating that missing a single hibernation cycle adversely affects health in thirteen-lined ground squirrels."*

(17) ▪ Alternatively, those that failed to exhibit torpor with or without a trial might be "less healthy" than those that did so. Thoughts?

Re. We thank the reviewer for raising this interesting point. To date, we are not aware of any published evidence indicating that failure to hibernate in a given season induces health issues in TLGS. Our experience shows that animals that do not hibernate in one year are able to hibernate normally in subsequent years without any apparent health consequences. While we acknowledge that TLGS are obligatory hibernators and that hibernation is a natural physiological behavior, our observations suggest that missing a hibernation cycle is not critical for health. Furthermore, routine health assessments conducted by our animal care staff did not reveal any signs of compromised health in animals that failed to enter torpor. Now stated in lines (also **see previous Reviewer #3, point #14):**

519-522: *"In addition, animals that did not undergo a hibernation cycle were evaluated twice per week throughout the study, and no health issues were observed; to date, we are not aware of any evidence indicating that missing a single hibernation cycle adversely affects health in thirteen-lined ground squirrels."*

(18) o What is one to make of published work reporting seasonal adjustments to metabolicallyrelevant mRNAs or peptides? What evidence might the manuscript present that would help us know whether or not a euthermic TLGS in December (when hibernation would generally be well underway) is molecularly equivalent to a euthermic TLGS in May (when prolonged euthermia concomitant with reproduction would generally be well underway)?

▪ TLGS: Epperson & Martin, 2002 https://journals.physiology.org/doi/full/10.1152/physiolgenomics.00004.2002
▪ Golden-mantled GS: Williams et al., 2005
https://journals.physiology.org/doi/full/10.1152/physiolgenomics.00301.2004
▪ TLGS: Mousavi et al., 2023 https://pubs.acs.org/doi/10.1021/acschemneuro.3c00268

Re. We thank the reviewer for highlighting these important studies. We fully acknowledge that published work in TLGS and other ground squirrel species (e.g., Epperson & Martin, 2002; Williams et al., 2005; Mousavi et al., 2023) demonstrates seasonal adjustments in metabolically relevant mRNAs, peptides, and other molecular mechanisms. These studies provide valuable insight into the molecular plasticity associated with hibernation and prolonged euthermia and are now cited accordingly (**see previous Reviewer #3, point #16).**

However, we would like to emphasize several points relevant to the present study:

1. **Focus on anatomical outcomes:** Our study specifically examines structural and anatomical changes in the retina and visual pathway, which are fundamentally different from molecular-level changes. While seasonal molecular fluctuations are well documented, our experience with TLGS over many years does not indicate major health issues associated with euthermia during winter; animals remain active, alert, and physiologically normal.
2. **Study duration and timing:** The current study spans nine months post-TBI. Since the injury was induced in summer, the subsequent nine months encompass sufficient time for degeneration to occur. Any degeneration resulting from the injury should still be detectable in our anatomical analysis. To perform a strictly seasonal molecular comparison (e.g., December vs. May) would have required the animals to enter at least one full hibernation period, which was not compatible with our experimental design. We intentionally maintained the animals in euthermic conditions to isolate the effects of TBI, without the confounding influence of active hibernation.
3. **Physiological relevance:** Although molecular profiles may fluctuate seasonally, our anatomical and functional outcomes are unlikely to be masked or significantly altered by these molecular adjustments.

The animals in December were physiologically awake, and our data indicate that retinal and visual pathway degeneration is measurable and interpretable under these conditions.

(19) • Because of the possible confound of a circannual rhythm of metabolic adjustment in the absence of expressed torpor, I would strongly suggest adding a Methods timeline graphic ranging from just before CHIMERA to last timepoint of data collection, with a parallel display of calendar month of data collection, animal age, room temperature and photoperiod, and what type(s) of hibernation monitoring was ongoing:
o Apparently no "naïve"/baseline data collection in December, just before CHIMERA was applied to the TBI group animals
o 3 mo post-TBI data collection at age 9.5 mo (presumably March, when the circannual rhythm would be calling for sexual maturity) (were signs of torpor monitored daily up to this time point? If so, say so)
o 6 mo post-TBI data collection at age 12.5 mo (presumably June, when the circannual rhythm might still be calling for last-chance reproduction) (were signs of torpor monitored daily up to this time point? If so, say so)
o 9 mo post-TBI data collection at age 15.5 mo (presumably September, when the circannual rhythm would be in hibernation preparation mode, and indeed some TLGS this age do exhibit test bouts of torpor beginning in August) (were signs of torpor monitored daily up to this time point? If so, say so)

Re. We thank the reviewer for raising this important and interesting point. Torpor signs in TLGS are typically assessed only in the fall, when animals are naturally preparing for hibernation. Our animal facilities perform routine health checks on all animals throughout the year (twice per week). While these checks do not specifically monitor for torpor outside the fall season, any behavior resembling hibernation during other times of the year would be noted as abnormal and flagged by veterinary staff as a potential health concern. Therefore, although daily torpor monitoring was not performed during non-hibernation periods, any inappropriate torpor-like behavior would have been detected.

We clarified this in the Methods, in the study design and in the CHIMERA induction (see previous **Reviewer #3, point #19**), and including a timeline graphic (Figure 1B) showing experimental interventions, calendar months, animal ages, room temperature, photoperiod, and hibernation-relevant monitoring periods to improve clarity for the reader (see previous **Reviewer #3, point #14**, for further details).

(20) • Starting in Methods at line 337, the manuscript cites an N=5 in the TBI group and an N=4 of in the"Naïve" (never-TBI) group. o Non-invasive data were collected at a time consistent with 3-6-9 mos post-TBI, with "additional control animals" included at 6-9 mos. In contrast, post-mortem data (e.g. IHC) data were collected only at 9 mos, meaning that certain acute TBI-induced changes were missed.

Re. We thank the reviewer for raising this important point. As this is the first study applying the TBI paradigm in a hibernating species, and given the limited availability of TLGS, we prioritized long-term evaluations to capture degenerative changes over a prolonged period. All animals were maintained in active (euthermic) conditions, and tissue collection at 9 months post-TBI allowed us to assess outcomes after sufficient time for degeneration to manifest.

It is also important to note that, based on previous studies in other models, many physiological alterations following TBI—beyond the direct acute injury—often appear well after the initial insult. These long-term changes could correlate with the vision loss observed in military personnel months or even years after injury. Therefore, we focused on long-term changes rather than the acute phase, during which we would not expect substantial RGC degeneration. Early post-mortem analyses could be informative in future studies, but for this first trial, our design maximized the ability to detect meaningful long-term retinal and visual pathway changes.

We made the appropriate clarifications in the study design, in **Reviewer #3 (point #15)**.

(21) o When the revision lays out a clear comparative summary of how well TLGS parallels documented human/rat/mouse acute responses to TBI, it should be careful to identify where "no data" were available in this TLGS longitudinal study. Might merit a mention in revised Study Design.

Re. As stated in an earlier response for the **reviewer 3 (point #5),** we agree that a comparative table summarizing TBI responses across species could be informative. While it is certainly feasible to revise the Discussion to more clearly indicate which prior studies are based on human versus rodent data and to emphasize findings most relevant to human pathology (the translational "gold ring"), we believe that a comprehensive cross-species comparison table would be better suited to a dedicated retrospective or review article rather than a first mechanistic study introducing this model in TLGS. Nevertheless, we revised the text to include and clarify species-specific references where appropriate.

(22) • I don't find the light dark box data useful because: wild TLGS hide in dark burrows. Indeed, they build short (~24") burrows solely as "escape hatches" that have no connection to the complex home burrow with nest, food cache, and defecatorium chambers. I would predict anxiety to enhance dark bias in TLGS, just as it does in nocturnal rats/mice (lines 305-6). Yet the paper suggests that light bias reflects anxiety in TLGS (lines 309-10). I just see too many alternative explanations to draw that conclusion (listed below), unless/until the appropriate un-injured TLGS controls are tested in exactly the same way across seasons -- and no such controls were used in this study.

o Could TLGS post-injury light bias be due to damage to the fear centers?

o Could TLGS post-injury light bias be due to acclimation to the procedure?

o Could TLGS post-injury light bias be due to circannual physiological state changes that don't depend on whether torpor itself has occurred, or not? (the 9 mo time point corresponds to September when the subjects' second hibernation season would be imminent)

Re: The authors appreciate the reviewer's interpretation and acknowledge that the light–dark box test in the thirteen lined ground squirrel is subject to multiple interpretations, which may limit its utility. We removed Figure 1 F-L from the manuscript and any corresponding text.

(23) • Immediately post-injury, TLGS were offered acetaminophen-dosed drinking water for 24 hr before and after TBI (line 363). It is not clear if NO-TBI controls also had this intervention. More importantly, the same citation (#47) used to justify this analgesic approach questions its efficacy (on p. 482, #47 says about drinking water acetaminophen: "consistent themes in all of these studies were variability in consumption, both in quantity and time of day that led the authors to conclude that these methods may be unreliable for provision of consistent and continuous analgesia." For the future studies mentioned, I might recommend subcu injections of Carprofen, 24&48 before and 24&48 days after TBI. We have found this quite effective on TLGS undergoing surgery.

Re. We thank the reviewer for this important point and for the thoughtful suggestion regarding alternative analgesic approaches. In our study, acetaminophen-dosed drinking water was provided 24hr before and up to 24hr after the final TBI exposure based on the recommendations of our collaborators at USUHS who have years of experience with the CHIMERA model in a variety of species and the approval of our veterinary staff. Uninjured control animals did not receive the acetaminophen intervention.

We acknowledge the cited reference's concerns regarding variability in analgesic intake from drinking water. However, as researchers, we are required to follow the analgesia protocols approved by our institutional animal care committee, as well as the Uniformed Services University Health Sciences (USUHS) animal protocol,

and any recommendations by the veterinarian staff. Any modification to the type, timing, or route of analgesia would need to undergo formal review and approval. We would be pleased to consider alternative analgesics, such as subcutaneous Carprofen, in future studies, pending committee approval. However, the lab is officially closed and no further studies will be allowed by the institute. For the current study, we strictly adhered to the approved protocol to ensure compliance with all ethical and regulatory guidelines.

(24) My concerns above are not an indictment of the work described in the manuscript under review. Because torpor is just one aspect of the circannual expression of hibernation physiology, I'm not convinced that a 9-month-long study reliant on body mass measurements and the lack of "observed signs of torpor" is truly capable of producing data "without the potential confounding neuroprotective influence of hibernation". The most this study can say, perhaps, is to have removed the potential confound of TORPOR. Reframing the study should reflect this reality.

I encourage the study authors to submit a revised version for re-review. The TLGS model has so much to offer, both in its relevance to the human condition and in its potential for deciphering novel protective strategies. That "double edged sword" is unavoidable, but ALL animal models – and indeed the variability amongst human patients – have their limitations of comparison.

Thank you for the opportunity of reviewing this interesting work.

Re. We thank the reviewer for this thoughtful and constructive comments, and we fully agree with the perspective expressed. We acknowledge that torpor is only one aspect of the circannual hibernation physiology and that our 9-month study, based on body mass and the absence of observed torpor, cannot definitively claim to remove all potential hibernation-related influences.

To reflect this more accurately, we reframed the manuscript to clarify that our study specifically removed the confound of torpor, rather than all aspects of hibernation physiology. We updated the text throughout the manuscript—including the Abstract, Introduction, and Discussion—to ensure this distinction is explicit.

We also appreciate the reviewer's encouragement regarding the translational relevance of the TLGS model and its potential for uncovering novel neuroprotective strategies. We agree that, as with all animal models, there are inherent limitations, and we highlighted these in the revised Discussion to provide appropriate context for our findings.

We hope you will find the referees' comments useful as you decide how to proceed. Should further experimental data or analysis allow you to address these criticisms, we would be happy to look at a substantially revised manuscript. In particular, the reviewers raised concerns if the study was adequately powered. We recommend a power analysis or at the very least discussions on sample size and report effect size for statistics; specify sex distribution on figure legends and provide sex annotated source data, and expand discussion to fully consider the strengths and weaknesses of the proposed model. You must also improve the reporting of methods and experimental design (see link to ARRIVE guidelines below), such as the administration of anaesthesia prior to perfusions (note that as different anaesthesia protocols were used for different applications, we do not consider removing repetitions in your Methods necessary if they allow for accuracy).

We are committed to providing a fair and constructive peer-review process. Do not hesitate to contact us if you wish to discuss the revision or if there are specific requests from the reviewers that you believe are technically impossible or unlikely to yield a meaningful outcome.

If you decide to submit a revised version, we ask that your manuscript complies with our editorial policies. Specifically:

For all graphs depicting a single point value (e.g., mean) with error bars, you must add individual data points or convert the graph to a boxplot or dot-plot to show data distribution.

It's mandatory to provide access to the numerical source data for graphs and charts either through a repository or by providing the data in a Supplementary Data file (in excel format).

All blots/gels must be accompanied by size markers in every figure panel. Uncropped and unedited blot/gel images must be included as Supplementary Figure(s) in the Supplementary Information pdf.

Please ensure that you have complied with the data deposition policies at the Nature Portfolio, please see here.

Please ensure that you have complied with our policies on research involving animals and humans, see here

Please follow the ARRIVE guidelines for reporting animal experiments. Please fully complete an ARRIVE checklist including both the essential and recommended set of items (adding information to the manuscript where needed) and upload this with your revised manuscript.

Please also see our revision checklist for guidance on formatting the manuscript and complying with our policies. A comprehensive guide to our formatting requirements for final submissions is also available for your reference here.

Title: Impact-Acceleration Head Injury Results in Optic Neuropathy in the Thirteen-Lined Ground Squirrel

We sincerely thank the Editor and Reviewers 1, 2, and 3 for their careful, constructive, and insightful evaluation of our manuscript. We greatly appreciate the time and effort they devoted to this thorough review, and we believe that their comments and suggestions have significantly improved the clarity, rigor, and overall quality of the work. All comments have been addressed point by point in the rebuttal below, and the manuscript has been revised accordingly. We hope that these revisions fully address the concerns raised by the Editor and Reviewers and meet the journal's expectations. To facilitate tracking of changes in the revised manuscript, previous edits are shown in blue, and newly added revisions are indicated in red.

Comments:

Reviewer #1 (Remarks to the Author):

The authors have made many changes to the manuscript in response to the reviewers' comments. However, there remain numerous points that require clarification, particularly regarding the statistical analysis, reporting of the results and interpretation of the findings.

1. The conclusions in the Abstract should be more carefully written to convey accurately the findings. The authors write "Longitudinal ophthalmic assessments revealed retinal thinning and significant reductions in RGC function, along with persistent deficits in visual acuity. Histological analysis confirmed dorsal RGC loss and late-stage changes in ON glia." What are the reductions in RGC function that are referred to here? There were no significant changes in retinal functional measures in Figure 2. Visual acuity deficits were observed at 3 months but not 6 or 9 months, so the characterization of these deficits as 'persistent' is questionable. The changes in ON glia were only for astrocytes, not microglia, so it would be more precise to state this as 'changes in ON astrocytes'.

R: The authors thank Reviewer 1 for carefully identifying these inconsistencies and for the opportunity to clarify and improve the manuscript. We have revised the text accordingly.

In addition, during a thorough re-examination of the electroretinogram (ERG) analysis, we identified an error in the reporting of the a-wave implicit time (see **point 3g**). The correct statistical outcome indicates a significant delay in the a-wave implicit time at 9 months post-injury. We have now corrected this throughout the manuscript, and the corresponding ERG results section, Discussion, and Abstract have been updated accordingly.

We also agree with the reviewer's comments regarding precision of terminology and interpretation of the histological findings. Accordingly, we have revised the manuscript to specify that GFAP expression in the optic nerve does not differ significantly between groups (see **point 12**), while statistically significant differences are observed in microglial activation (see **points 7 and 11**). The Abstract and main text have been updated to accurately reflect these distinctions. Finally, we have revised the description of the temporal profile of visual acuity changes to avoid the term "persistent," in accordance with the reviewer's suggestion.

Page 2, Lines 53-61: *"Longitudinal ophthalmic assessments revealed transient retinal thinning and deficits in visual acuity, accompanied by a subtle delay in the a-wave implicit time of the electroretinogram (ERG) and significant reductions in RGC function at 9 months post-injury, as measured by patter ERG amplitude. Histological analysis further demonstrated dorsal RGC loss and late-stage regionally restricted microglia activation in the ON. Together, these findings demonstrate that the thirteen-lined ground squirrel recapitulates key aspects of the heterogeneous*

systemic, behavioral, and visual dysfunction following TBI, establishing it as a promising model for neurotrauma and vision loss."

2. The housing conditions are not detailed for the subset of ground squirrels that were moved to the hibernaculum to allow hibernation (in support of collecting weight data in Figure 1?). The alterations to vivarium temperatures (shown in Figure 1) were not described in the housing section.

R: The authors thank reviewer 1 for pointing out this absence of housing details, we have now included detailed description accordingly in material and methods section:

Page 14, Lines 507--513: *Animals were then implanted with subcutaneous temperature transponders for continuous monitoring of core body temperature and housed individually under standard laboratory conditions with seasonally adjusted light–dark cycles designed to replicate natural photoperiods in Wisconsin (Figure 1B). Briefly, environmental temperature (gray bars) was gradually increased from 60 ± 4 °F in December to 72 ± 4 °F by late spring–summer, with incremental changes of ~1 °F each two-week period. In parallel, the photoperiod (orange trace) was extended from 8.5–9 hours of light in December–January to approximately 15 hours in June, increasing by ~1.5 hours per month."*

Page 15, Lines 536-546: *"To control for potential differences in body weight associated with hibernation, a subset of Thirteen-lined ground squirrels (n = 6) was allowed to enter hibernation in the fall after exhibiting signs of spontaneous torpor. These animals were transferred by veterinary personnel from standard housing conditions (ambient room temperature) to a dedicated hibernaculum maintained at 4 °C, as reflected in Figure 1C. Following transfer, animals were monitored daily, and after three consecutive days in stable torpor (confirmed by a body temperature of ~4 °C) they were placed in individual containers with appropriate bedding, without access to food or water, consistent with standard hibernation protocols. Health status was assessed twice weekly, and body weight was recorded periodically throughout the hibernation period. Importantly, these animals were used exclusively for body weight monitoring and were not subjected to any experimental procedures or included in subsequent analyses."*

3. The statistical analysis section and reporting within the text/legends is not complete. a) It would be easier for the reader to follow if references were provided to the data figures for each test described. For example, "OCT measurements of RNFL and total retinal thickness (Figs 2B and 2C) were analyzed using repeated-measures one-way ANOVA in GraphPad Prism (version 10.5.0), as the dataset included pre-injury baseline measurements and follow-up assessments 4.5- and 9-months post-injury." This would also help the authors verify that they have provided methods for each dataset.

R: The authors thank Reviewer 1 for highlighting the need for improved consistency and clarity in the reporting of statistical analyses. Statistical tests were performed for all datasets included in the manuscript. In response to this comment, we have revised the OCT Results section to explicitly include the statistical analyses, as suggested. In addition, to ensure comprehensive and easily traceable reporting, and **following Reviewer 1's recommendation (point 3c),** we have included a summary table (**Supplementary Table 1**) that compiles all statistical analyses. This table is now systematically referenced throughout the manuscript for each dataset.

Page 5, Lines 169-188: Because ON injury is expected to reduce the number of RGCs, we examined thinning of the retinal nerve fiber layer (RNFL) as an indicator of axonal loss. However, RNFL thinning was not anticipated within the timeframe of this study, as previous studies have shown that retinal thickness in thirteen-lined ground squirrels remains stable with aging[47]. Optical coherence tomography (OCT) measurements were therefore focused on the

visual streak, a specialized retinal region characterized by the highest density of cones and RGCs. Importantly, due to the horizontal orientation of the ON head (ONH), which effectively divides the retina into superior and inferior hemiretinas, a large proportion of RGC axons from regions below the ONH converge through the visual streak[14-19]. This makes it a particularly reliable and anatomically consistent region for detecting changes in RNFL thickness in the thirteen-lined ground squirrel[14].

Analysis of the visual streak revealed that RNFL thickness (**Figure 2A, yellow arrow**) was not significantly reduced in injured animals at 9 months post-CHIMERA compared to the pre-injury (baseline) levels from the same animals (**Figure 2B;** repeated-measures one-way ANOVA, $F(1.389, 12.50) = 2.075$, $p = 0.1730$; **Supplementary table 1**). In contrast, total retinal thickness was significantly reduced at 4.5 months post-injury (**Figure 2C;** repeated-measures one-way ANOVA, $F(1.946, 17.51) = 15.34$, $p = 0.0002$; Tukey's post hoc for multiple comparisons, $p = 0.0014$; **Supplementary table 1**). By 9 months post-injury, retinal thickness was no longer significantly different from baseline but was significantly increased relative to the 4.5-month time point (**Figure 2C**; Tukey's post hoc for multiple comparisons, $p = 0.0079$; **Supplementary table 1**).

b) In the previous sentence, statistical software is referenced for this one test, but not for any of the other tests. If all tests were run in GraphPad Prism v 10.5.0, this statement should stand alone, not in association with one test.

R: The authors thank reviewer 1 for requesting consistency and clarity. Because all statistical analysis were performed using the same software and version, we have now stated it in the Statistics section in Material and methods:

Page 22, Lines 827-830: *"Statistical analyses were conducted using GraphPad Prism (version 10.5.0). A comprehensive summary of the statistical tests applied, including results and significance levels, is presented in **Supplementary Table 1**."*

c) ANOVA results are not consistently reported. The authors should report all ANOVA F test results and p values. F tests should be reported as F(x,y)=z, p=xx or Fx,y=z, p=xx. For 2-way ANOVAs or 1-way ANOVAs with repeated measures, both main effect and interaction ANOVA results should be provided. This can be done in the results text, the figure legends or in a table.

R: The authors thank Reviewer 1 for emphasizing the importance of consistent and complete reporting of ANOVA results. In accordance with this recommendation, results section has been revised to refer to the ANOVA outputs (F values, and corresponding p values) where appropriate. To improve clarity, we have compiled all statistical analyses, including full ANOVA outputs (F values, and corresponding p values) and post hoc tests results, in a summary table (**Supplementary Table 1**) for all datasets analyzed in this study.

d) Post-hoc t-tests used for ANOVAs are not described. Consideration should be given to correcting for multiple comparisons.

R: The authors thank Reviewer 1 for highlighting the need for clearer reporting of post hoc analyses and multiple-comparison corrections. In response, we have now specified the post hoc tests used following ANOVA in the Statistical Analysis section of Materials and Methods. These tests, along with the applied multiple-comparison corrections, are now systematically referenced to results section and detailed in the **Supplementary Table 1.**

e) Lines 167-169, the authors state that RNFL thickness "decreased progressively with time" despite no

significance at either time point relative to baseline and no notable change between 4.5 and 9 months. This is an overinterpretation. Further, no ANOVA results are reported. (see point c).

R: The authors thank Reviewer 1 for identifying this overinterpretation and inconsistency. We have revised the text to remove the statement that RNFL thickness "decreased progressively with time," as this was not supported by statistically significant differences (see **point 3a**). In addition, the corresponding ANOVA results are now explicitly reported, as addressed in **point 3a.** Following the recommendation in **point 3c,** all statistical details are also compiled in **Supplementary Table 1**, which is now referenced in the revised text for clarity and completeness.

f) Authors should be clear about whether p values reported are for the ANOVA or the posthoc tests. For example, on page 5 the authors write "In contrast, total retinal thickness was significantly reduced at 4.5 months post-injury (Figure 2C; p=0.0014; repeated-measures ANOVA, n=10 eyes). By 9 months post-injury, retinal thickness was no longer significantly different from baseline but was significantly increased relative to the 4.5-month time point (p=0.008) Figure 2C; repeated-measures ANOVA, F=15.34, p=0.0002)," This is confusing, because the there should only be one repeated measures ANOVA run for Figure 2C, which should be reported (F test and p value for the ANOVA) prior to reporting posthoc t-test p values. It is unclear whether the two different p values refer to the ANOVA or to the posthoc tests.

R: The authors thank Reviewer 1 for highlighting the need for clearer distinction between ANOVA and post hoc test results. We have revised the text to ensure that ANOVA results (including F values and corresponding *p* values) are reported prior to post hoc comparisons, and that *p* values are explicitly attributed to either the overall ANOVA or the post hoc tests. This specific issue has been corrected in the revised Results section (see **point 3a**). In addition, and following the recommendation in **point 3c**, all statistical details are systematically compiled in **Supplementary Table 1**, which is now referenced throughout the manuscript for clarity and consistency.

g) It is unclear how data in Figure 2 F-I were analyzed, despite a reference to no "significant" changes on line 186, which implies statistical analyses. The statistical tests should be described in the Statistical analysis section and the results reported (i.e. non-significant ANOVAs for main/interaction effects).

R: The authors thank Reviewer 1 for highlighting the need for clearer reporting of statistical analyses. We have revised the statistical approach and confirm that significant changes in implicit time are present, while no significant effects were detected for amplitude measures. Accordingly, the Results and discussion sections (see below) and figures have been updated to reflect these findings, and the corresponding statistical tests are now explicitly described. Given that amplitude and implicit time were analyzed across multiple stimulus conditions, each requiring separate statistical testing, we have centralized all detailed results, including ANOVA outputs and post hoc comparisons, in **Supplementary Table 1**, which is now systematically referenced throughout the Results section.

Page 6, Lines 199-212: *"Neither the ERG a-wave nor the b-wave showed significant changes in amplitude (**Figure 2F-G**) in CHIMERA-injured animals (n = 10 eyes, 5 animals) compared with age-matched naïve controls at the 4.5-month (n = 8 eyes, 4 animals) and 9- month (n=12 eyes, 6 animals) post-injury time points (non-significant ordinary one-way ANOVA and Tukey's post hoc for multiple comparisons test, see **Supplementary table 1**). However, implicit time for a-wave for most of the stimuli presented, and only punctually in b-wave (**Figure 2H-I**), showed significant increase at 9 month post-CHIMERA respect to 4.5 months post-CHIMERA (ordinary one-way ANOVA and Tukey's post hoc for multiple comparisons test, see **Supplementary table 1** for details). Regarding implicit time changes, we also observed sporadic differences between naïve, 4.5-month and 9-month groups, however, these were of much smaller magnitude and less consistent than those observed following CHIMERA injury (see **Supplementary***

table 1 for details). Together, these results indicate that CHIMERA injury does not measurably affect outer retinal response amplitude, but is associated with a subtle, time-dependent slowing of phototransduction kinetics.

Page 9, Lines 326-330: *"ERG recordings showed no deficits in photoreceptor and bipolar cell response amplitude, but is associated with a subtle, time-dependent slowing of phototransduction kinetics, which may reflect mild alterations in photoreceptor response dynamics and/or early changes in post-receptor signal transmission without overt synaptic or cellular loss."*

h) There is no description of the statistical analysis for data in Fig 2 M-O.

R: The authors thank reviewer 1 for requesting consistency and clarity. In addition to the **Supplementary Table 1**, we have included the following sentence:

Page 7, Lines 217-221: *"Post-injury animals showed no frequency-dependent changes in response amplitude compared with naïve animals even at 30Hz (**Figure 2M-O**; Ordinary One-way ANOVA; 5Hz: F(3, 36)=1.221, p=0.3334; 15Hz: F(3, 36)=0.5753, p =0.6350; 30Hz: F(3, 36)=0.3016, p=0.8240, **Supplementary table 1**), indicating that cone responses are not impaired by the mild closed head impact used in this study.*

i) On page 6, the authors' statement that "This reduction in OMR persisted at 6- (1.26 ± 0.32 cyc/deg) and 9-months (1.36 ± 0.25 cyc/deg) post-injury" is not supported by the statistical analysis which shows no difference, as they report: "… although the difference at these later time points no longer reached statistical significance." Given the very small n for this study, the authors must be more cautious about overinterpreting nonsignificant shifts in distribution. The title of this section (lines 200-201) should also be modified accordingly.

R: The authors thank Reviewer 1 for identifying this inconsistency. We have revised the text to remove the unsupported interpretation of a sustained reduction in OMR at later time points, and the section title has been accordingly modified to better reflect the statistical outcomes (see below). In addition, this section has been updated to consistently report all statistical analyses, including non-significant results, in line with the reviewer's recommendation (see revised manuscript).

Page 7, Lines 229-230: *"The difference in visual acuity at 6- (1.26 ± 0.32 cyc/deg) and 9-months (1.36 ± 0.25 cyc/deg) post-injury no longer reached statistical significance."*

Page 7, Line 223: *"CHIMERA-Induced TBI Causes Subtle Deficits in Visual Acuity Measured by Optomotor Response"*

j) On page 7, in describing the PERG data, the authors describe one posthoc result, then the overall ANOVA, then another posthoc result. It would be clearer to first report the overall ANOVA, and then the posthoc comparisons.

R: The authors thank Reviewer 1 for highlighting the need for clearer and more consistent reporting of statistical results. We have revised the PERG results paragraph to ensure that statistical outcomes are presented in a logical order, first reporting the overall ANOVA results followed by the corresponding post hoc comparisons. Where appropriate, we now also refer to **Supplementary Table 1** for complete statistical details, in accordance with the standardized reporting format used throughout the manuscript:

Page 7, 240-252: *"To corroborate the visual deficits observed with the optomotor reflex, we also measured pattern electroretinography (PERG) in the same animals before injury and at 3-, 6-, and 9-months post-injury to track longitudinal changes in retinal function. PERG provides a measure of RGC activity, with reductions in amplitude*

reflecting loss of RGC function. At 9 months post-injury, TBI-exposed thirteen-lined ground squirrels showed a significant reduction in PERG amplitude (50.3 ± 22.5 µV, n = 10 eyes) compared to their pre-injury baseline (81.6 ± 24.3 µV, n = 10 eyes; ***Figure 3C-D****). Repeated-measures one-way ANOVA confirmed a significant effect over time (F(2.383, 21.45)=3.977, p=0.0281), with Tukey's post hoc for multiple comparison analysis indicating that the reduction in amplitude occurred between 6- and 9-months post injury (p=0.0345,* ***Supplementary table 1****). In contrast, the implicit time of the P50 peak in the PERG response did not change significantly from 30.2 ± 4.0 ms at baseline in the naïve condition to 33.7 ± 2.8 ms at 9 months post-injury (****Figure 3E****; Repeated-Measures one-way ANOVA, F(2.353, 21.18)=2.613, p=0.0894;* ***Supplementary table 1****).*

k) The statistical analysis section says "Retinal vasculature parameters were compared between baseline pre-injury images and 9 months post-CHIMERA injury using Student's t tests." but Figure and Figure legends describe the control group as naïve not pre-injury. There is also a redundant sentence fragment in this part of the statistical analysis section.

R: The authors thank the reviewer for pointing out this inconsistency, and have corrected it in the text. The sentence fragment has been removed from the text.

Page 23, Lines785-787: *"Retinal vasculature parameters, microglial cell density (Iba1 cells per unit area) in retina and ONs, glial fibrillary acidic protein (GFAP) integrated density, and the weight of different organs (spleen and kidneys), were compared between naïve and 9 months post-CHIMERA injury using unpaired two-tailed Student's t tests"*

l) Please specify in the Statistical Analysis section whether student's t-tests were 2-tailed or 1-tailed, paired or unpaired. Then this information does not need to be provided in the Results text.

R: The authors thank Reviewer 1 for highlighting the need for clearer specification of statistical tests. All Student's *t*-tests performed in this study were unpaired and two-tailed. This information has now been explicitly stated in the Statistical Analysis section of the Materials and Methods, where the tests used for each dataset are described (see below). For consistency in reporting, statistical tests are also indicated in the Results section (as done for ANOVA analyses), and all details are systematically compiled in **Supplementary Table 1**.

Page 22, Lines 826-857: *"Statistical Analysis*

All statistical analyses were conducted using GraphPad Prism (version 10.5.0), except for the indocyanine green (ICG) vascular intensity (Levene's test calculator available from Statistics Kingdom 2017). A comprehensive summary of the statistical tests applied, including results and significance levels, is presented in ***Supplementary Table 1****. OCT measurements of RNFL and total retinal thickness were analyzed using repeated-measures one-way ANOVA and Tukey´s post hoc test for multiple comparisons, as the dataset included pre-injury baseline measurements and follow-up assessments 4.5- and 9-months post-injury. Amplitude and implicit time of the ERG a- and b-waves, as well as flicker response amplitudes (5, 15, and 30 Hz), were analyzed using ordinary one-way ANOVA followed by Tukey's post hoc test for multiple comparisons, comparing age-matched naïve control animals with CHIMERA-exposed animals. OMR visual acuity measurements were analyzed using ordinary one-way ANOVA and Tukey´s post hoc test for multiple comparisons, as age-matched naive control animals and CHIMERA-exposed animals were compared. Repeated-measures analysis was not applied because the naïve control animals were not longitudinally matched to the CHIMERA cohort; control animals were assessed at 0 and 9 months, whereas CHIMERA animals were assessed at 3-, 6-, and 9-months post-injury. Pattern ERG amplitudes and implicit times were analyzed using repeated-measures one-way ANOVA and Tukey´s post hoc test for multiple comparisons,*

consistent with the inclusion of pre-injury baseline measurements and follow-up measurements at 4.5- and 9-months post-injury. Retinal vasculature parameters, MC density (Iba1 cells per unit area) in retina and ONs, glial fibrillary acidic protein (GFAP) integrated density, and the weight of different organs (spleen and kidneys), were compared between naïve and 9 months post-CHIMERA injury using unpaired two-tailed Student's t tests. The ICG vascular intensity histogram and confidence intervals were plotted using Levene's test calculator [Internet] Statistics Kingdom 2017 [cited 18 September 2025] (Available from: https://statskingdom.com/230var_levenes.html and verified using the R software package). RGC (RBPMS) density in the retina was initially analyzed using ordinary one-way ANOVA with Tukey's post hoc test for multiple comparisons. However, because thirteen-lined ground squirrel RGC density is known to differ between dorsal and ventral retina[14] and this was not the focus of the present study, direct comparisons between naïve and 9-month post-CHIMERA groups were performed using unpaired two-tailed Student's t-tests. Microglial activation (Iba1/CD68 co-localization) was analyzed using Fisher's exact test by comparing the proportion of ONs exhibiting activated microglia between naïve and CHIMERA-treated groups."

m) The authors, in their response to the previous critique that two eyes or nerves in the same animal are not independent measures, provide a statement in their 'definition of n' that "where applicable, bilateral measure were analyzed using repeated-measures ANOVA designs". This approach does not seem to have been incorporated into any of the analyses. Further, the authors statement that the injury is "equivalent" to both eyes and nerves of one animal is not supported by any evidence and is contradicted by their statement that the TBI can "differentially affect each visual pathway". Further, if the injury (mechanically) is equivalent to both sides of the visual system, as the authors suggest, this is in support of the necessity of treating the left and right samples as repeated measures rather than independent samples. That is, the responses within an animal may be more similar than those across animals. This can be tested statistically, but the fact that some animals were sampled once (R or L) while others were sampled bilaterally (R and L) as revealed in the supplementary Excel file provided with this revision, makes testing this assumption virtually impossible. The lack of systematic sampling is a limitation of the study design that needs to be included.

R: The authors thank Reviewer 1 for this thoughtful comment regarding the treatment of bilateral samples and the statistical approach. We agree that, in principle, when measurements from both sides of the same animal are systematically collected, a repeated-measures design may be appropriate to account for potential intra-animal correlations.

However, we respectfully clarify that in the context of the CHIMERA model, it is important to distinguish between the **mechanical delivery of the injury** and the **resulting biological response**. While the impact is applied at the midline -therefore being mechanically comparable with respect to both optic nerves- this does not ensure equivalent biological outcomes. In traumatic brain injury models, variability arises from subtle anatomical asymmetries, differences in force transmission, and local tissue susceptibility. Consequently, the extent, localization, and progression of axonal damage, as well as associated cellular responses, may differ between the left and right optic nerves within the same animal.

Consistent with this, our data show heterogeneous patterns of degeneration and microglial activation between sides, supporting the notion that bilateral measurements cannot be assumed to be equivalent or strongly correlated. Therefore, treating each optic nerve as an independent experimental unit reflects the biological variability inherent to this model rather than a contradiction of the mechanical setup.

Importantly, we acknowledge that bilateral sampling was performed systematically across all animals or analyses, however, for some ex vivo analysis not all samples from the same individual undergo same staining combination. This heterogeneity precludes the consistent application of repeated-measures statistical approaches and limits the ability to formally assess intra-animal dependence. We agree that this represents a limitation of the study design. Accordingly, we have revised the manuscript to clarify this point and now explicitly state:

Page 13, Lines 476-483: *"In this study, although the traumatic impact was delivered at the head midline, the biological outcome may differ between sides due to inherent variability in TBI models. Thus, for all groups, both eyes and ONs from each animal were analyzed and treated as independent experimental units (OCT, ERG, PERG, and ex vivo anatomical analysis), except for the behavioral assessment (OMR), where each animal was an experimental unit. While bilateral sampling was performed, staining combinations were not applied systematically across all ex-vivo analyses, precluding the use of repeated-measures statistical approaches and limiting the assessment of intra-animal correlations."*

4. In the legend for Figure 2, the box and whisker plots are described as "Error bars represent the 90th (top) and 25th (bottom)". This is a nonstandard description and seems incomplete and in error. The middle line and top and bottom of the box should be defined (often these represent median and quartiles), and the whiskers (error bars) should be defined (often these represent the 95th percentile, or maybe the 90th percentile in this case?). This explanation should be included in each figure containing box plots. Alternatively, the explanation could be in the Statistical Analysis section to describe how all box plots are represented.

R: The authors thank reviewer 1 for pointing out this error and for requesting consistency and clarity. We have now removed this sentence from the Fig. 2 legend. Accordingly, we have now edited figure legends, and included a corrected description for all box plots in the Statistical Analysis section as recommended:

Page 23, Lines 858-862: *Data are presented as box plots (GraphPad Prism, version 10.5.0), in which the central line represents the median and the upper and lower box boundaries indicate the upper and lower quartiles, respectively. Whiskers extend to the 90th (upper) and 10th (lower) percentiles. Individual data points are shown as dots. Statistical significance is indicated as follows: $p \leq 0.05$ (*), $p \leq 0.01$ (**), $p \leq 0.001$ (***), and $p \leq 0.0001$ (****).*

5. On page 5-6, the authors state "By 9 months post-injury, retinal thickness was no longer significantly different from baseline but was significantly increased relative to the 4.5-month time point …suggesting that prolonged retinal inflammation may contribute to increased cellular thickness at this later stage." It is unclear how any inference about inflammation can be drawn from retinal thickness measurements. This speculative comment should be removed from the Results.

R: The authors thank the reviewer for pointing out this speculation, and have removed it from the text.

6. Statements (page 8) such as "This likely reflects the fact that microglia are primarily recruited to sites of active degeneration and debris clearance. Following the initial injury and subsequent wave of neurodegeneration, the inflammatory response diminishes in regions where RGCs have already been lost, leading to a reduced microglial presence over time" are speculative interpretation and belong in the Discussion, not the Results.

R: The authors thank the reviewer for pointing out this speculation, and have moved it to the Discussion.

Page 10, Lines 345-353: *"This likely reflects the fact that microglia are primarily recruited to sites of local axonal degeneration and debris clearance. Following the initial injury and subsequent wave of neurodegeneration, the inflammatory response diminishes in regions where RGCs have already been lost, leading to a reduced microglial presence over time. These events were accompanied with subtle alterations in retinal vascular diameter and branching and changes in microglial markers in the ON, as reported previously[53] which are consistent with chronic post-injury pathology."*

7. Similarly, "certain ON sections display microglial activation, possibly associated with localized axonal degeneration." The latter part of this sentence is speculation that could be provided in the Discussion but not the Results.

R: The authors thank the reviewer for pointing out this speculation. We have provided a new analysis to support significant microglial activation in ONs subjected to CHIMERA (see **point 11**). We have accordingly edited the sentence in results while moving the speculative paragraph to the Discussion.

Page 9, Lines 308-312: *"Nonetheless, the IBA1 and CD68 co-localization indicate that 71% of ONs subjected to CHIMERA exhibit microglial activation, whereas none of the control ONs showed an increased CD68 signal (5/7 vs. 0/6 ONs in CHIMERA and control groups, respectively; Fisher´s exact test: P value=0.0210*, Two-sided; **Supplementary table 1**)."*

Page 10, Lines 351-353: *"In this context, the increased prevalence of localized microglial activation (Iba1/CD68 co-localization) in ONs following CHIMERA is likely associated with ongoing focal axonal degeneration."*

8. The statement "TBI-exposed thirteen-lined ground squirrels exhibited long-term deficits in RGC function, as evidenced by reduced pattern ERG amplitudes and delayed implicit times" on page 9 is not supported by the data, which showed no change in implicit times. Further, the PERG data shows a delayed decrease in RGC function, which is somewhat different than 'long-term deficits' which could be interpreted as deficits that persist for a long time.

R: The authors thank the reviewer for making this clarification, and have corrected the text accordingly. Discussion of delayed implicit times has been removed:

Page 9, Lines 323-325: *"TBI-exposed thirteen-lined ground squirrels exhibited persistent deficits in RGC function, as evidenced by reduced pattern ERG amplitudes, consistent with findings from other studies of blast-induced injury[51,52]."*

9. The statement that " in vivo anatomical evaluation using OCT revealed a gradual thinning of the RNFL" is not supported by the data, which 1) does not show a statistically significant change and 2) shows an equivalent median at 4.5 and 9 months. There is no acute data, so the authors do not know if this nonsignificant decrease was 'gradual'.

R: The authors thank reviewer 1 for making this clarification, and have corrected the text accordingly.

Page 10, Lines 334-339: *"In agreement with these functional and behavioral results, in vivo anatomical evaluation using OCT revealed a significant thinning of the total retinal thickness at 4.5 months post-injury and a subsequent increase in thickness at 9 months, possibly reflecting dynamic structural remodeling processes rather than sustained*

degeneration, potentially involving glial responses such as Müller cell or astrocyte reactivity, as well as inflammation-associated tissue changes that can temporarily influence retinal thickness."

10. The statement "…total retinal thickness was revealed to be significant at 4.5 months post-injury and a subsequent increase in thickness at 9 months, possibly reflecting an immune cell-mediated response. This degeneration…" is also highly speculative. What evidence is there of immune cell mediated increase in retinal thickness? The authors data show no change in retinal glia. Further, what evidence is there that the decreased retinal thickness represents 'degeneration', if the change is transient? RGC dysfunction does not equate to degeneration.

R: The authors thank Reviewer 1 for raising this important point and for the opportunity to clarify our interpretation. We acknowledge that the term "immune cell-mediated response" was inaccurate and have corrected this to "glia-mediated response" in the revised manuscript.

We agree that our previous wording was overly speculative and have revised the text accordingly. Although we did not detect a significant increase in microglial cell numbers, inflammatory responses following optic nerve injury are not limited to changes in microglial density. Traumatic optic nerve injury is well known to induce a complex neuroinflammatory response involving activation of resident microglia, astrocytes, and Müller glia, as well as the release of cytokines and recruitment of peripheral immune cells.

Importantly, experimental models of optic nerve injury have demonstrated that retinal inflammation and gliosis occur rapidly after injury, including upregulation of inflammatory mediators (e.g., TNF-α, IL-1β) and gliosis markers such as GFAP and Iba1, even in the absence of overt changes in cell number. In addition, reactive gliosis of Müller cells and astrocytes is a hallmark of retinal and optic nerve injury and can lead to structural remodeling of the retina.

Notably, inflammation-associated retinal thickening has been described in models of optic nerve and retinal injury, where early inflammatory edema and glial activation can transiently increase retinal thickness and mask underlying neurodegenerative changes. These findings support the concept that changes in retinal thickness may reflect a dynamic balance between degeneration and inflammation-driven structural remodeling.

In this context, the transient reduction in retinal thickness observed at 4.5 months may reflect early structural alterations associated with RGC dysfunction or stress. However, because this effect is not sustained, we have revised the manuscript to avoid interpreting it as definitive degeneration. The subsequent return to baseline thickness at 9 months may instead reflect compensatory or reactive glial responses, including Müller cell or astrocyte hypertrophy and/or inflammation-associated tissue remodeling.

Page 10, Lines 334--339: *"In agreement with these functional and behavioral results, in vivo anatomical evaluation using OCT revealed a significant thinning of the total retinal thickness at 4.5 months post-injury and a subsequent increase in thickness at 9 months, possibly reflecting dynamic structural remodeling processes rather than sustained degeneration, potentially involving glial responses such as Müller cell or astrocyte reactivity, as well as inflammation-associated tissue changes that can temporarily influence retinal thickness."*

11. The statement "These events were accompanied with subtle alterations in retinal vascular branching and changes in microglial markers in the ON" is not accurate, as there were no changes in microglial markers (Iba1) in the retina or ON. The CD68 labeling was reported anecdotally. If the authors wish to make a claim about changes in microglial in the ON, they need to perform a more complete examination of CD68 immunolabeling.

R: The authors thank reviewer 1 for requesting statistical evidence for our observations. We have now quantified as the proportion of ONs exhibiting microglial activation versus those without detectable activation in both experimental and control groups. We performed the Fisher exact test for presence of activated Microglial cells in the ON analyzed. Accordingly, we have included details in material and methods, statistics and results sections as well as its corresponding plot in Fig. 5C. This test provides evidence of microglial cell activation in 71% (n=5/7) of the ONs at 9 months after CHIMERA, while 0% (n=0/6) of ONs had activated microglial cells in Naïve animals (also in **point 7**).

Page 22, Lines 810-818: Microglial activation in the ON was evaluated by assessing the co-localization of Iba1 and CD68 immunoreactivity. Focal CD68-positive microglial activation was clearly detected in specific branches in most ONs subjected to CHIMERA; however, widespread activation across the entire ON section was not detected. Given the relatively large diameter and complex architecture of the ON of the thirteen-lined ground squirrel, compared with those of mice or rats, only a subset of sections was analyzed. Consequently, additional localized regions of degeneration and associated microglial activation may have been underrepresented. Therefore, microglial activation was quantified as the proportion of ONs exhibiting microglial activation versus those without detectable activation in both experimental and control groups.

Page 9, Lines 308-312: *"Nonetheless, IBA1 and CD68 staining indicate that 71% of ONs subjected to CHIMERA exhibit microglial activation, whereas none of the control ONs showed an increased CD68 signal (5/7 vs. 0/6 ONs in CHIMERA and control groups, respectively two-sided Fisher´s exact test, p=0.0210; **Supplementary table 1**)."*

Page 23, Lines 855-857: *"Microglial activation (Iba1/CD68 co-localization) was analyzed using Fisher's exact test by comparing the proportion of ONs exhibiting activated microglia between naïve and CHIMERA-treated groups."*

12. It is unclear how the data in this study are consistent with reports of axonal injury in rodent TBI (lines 312-315), since no assessment of axonal pathology was performed and GFAP expression decreased, which is opposite of the typical response to white matter injury.

R. The authors thank Reviewer 1 for this important observation and for the careful evaluation of our results. We agree that our initial analysis of GFAP expression in the optic nerve was not fully aligned with the expected response to white matter injury described in rodent traumatic brain injury studies.

In response, we have reanalyzed the GFAP integrated intensity using a more stringent and controlled approach. Specifically, quantification was restricted to a defined 0.2 × 1 mm region of interest in naïve optic nerves and those subjected to CHIMERA at 9 months. To minimize technical variability, we included only samples that were sectioned on the same day, mounted on the same slide, and processed simultaneously during immunostaining. As a result, a subset of previously included optic nerves was excluded to ensure appropriate comparability.
Importantly, this revised analysis led to a change in the statistical outcome: no significant differences in GFAP integrated intensity were detected between groups using an unpaired two-tailed t-test. These updated results have been incorporated into the revised manuscript, including corresponding changes in the Methods, Results, Figures, Abstract, and Conclusions. The revised dataset has been included in the updated Excel file (row data) provided for review, and the new statistical outputs are reported in **Supplementary Table 1**.

Page 22, Lines 819-824: *"Quantification of GFAP immunoreactivity in the optic nerve was performed using ImageJ (FIJI, NIH) by measuring integrated density normalized to the corresponding optic nerve area. To minimize technical*

variability, only samples that were sectioned on the same day, mounted on the same slide, and processed simultaneously during immunostaining were included. For each optic nerve, a region of interest (ROI) measuring 0.2 × 1 mm was defined, and fluorescence intensity was quantified using the integrated density function."

Page 9, Lines 312-315: *"In contrast, glial fibrillary acidic protein (GFAP) expression did not differ significantly between groups (**Figure 5D-E)** as quantified in comparable regions of interest adjacent to the ONH **(Figure 5F-G**; unpaired two-tailed student's t test, p=0,6757; n=3 and 6 ONs for naïve and 9-months post-CHIMERA, respectively; **Supplementary table 1**).*

Page 10, Lines 359-362: *"In addition, although previous studies have reported increased GFAP immunoreactivity following TBI[62–64], we did not observe differences in GFAP levels in ON astrocytes at 9 months post-injury, which may reflect a resolution of astrocytic reactivity at chronic time points, highlighting the dynamic and heterogeneous nature of glial remodeling following TBI."*

13. Given the authors admission that availability of the ground squirrels and their protected status constrained their experimental design, these considerations should be provided as limitations to the model in the Discussion.

R: The authors thank the reviewer for suggesting this additional limitation be added to the text.

Page 12, Lines 442-447: *"Due to the thirteen-lined ground squirrels' classification as a USDA-protected species, the experimental design required careful optimization to minimize animal numbers and experimental attempts while still allowing determination of whether TBI induces measurable alterations in the visual pathway. This pilot study provides foundational parameters for generating closed-head impact TBI in this model and is intended to guide future research while facilitating more efficient ACUC approval."*

Reviewer #2 (Remarks to the Author):

I am happy with the revisions made and have no further comments.

R: The authors thank reviewer 2 for their careful evaluation of our manuscript.

Reviewer #3 (Remarks to the Author):

I thank the Authors for the comprehensive responses to all the Editor's and Reviewers' comments. I am grateful for the Reviewers for catching things I missed.
The Rebuttal responses to MY particular concerns have been met in the revision.
If the other Reviewers feel the same way, I consider this Revision to be a success worthy of publication.
Nevertheless there are two small points:

1. The word "scull" should be spelled "skull".

R: The authors thank reviewer 3 for pointing out this typo and have corrected it in the text.

2. The new text at the Revision's Lines 571-2 should reads: "...despite multiple publications using mice that report

damage from one impact(92-96)" [add "using mice"]. It's for a different paper I'm sure, but I wonder how much the difference in skull morphology plays in the differential susceptibility to a single impact.

R: The authors thank reviewer 3 for bringing up this interesting point and have made the suggested changes to the text.

Page 17, Lines 613-642: *"None of these conditions produced detectable pathological changes in the visual pathway probably due to the CHIMERA model, the injury location, the animal model and/or the animal anatomy, such as the skull thickness, despite multiple publications using mice that report damage from one impact[92–96]."*

Title: Impact-Acceleration Head Injury Results in Optic Neuropathy in the Thirteen-Lined Ground Squirrel

We thank the Editors and Reviewers 1, 2, and 3 for their careful, constructive, and insightful evaluation of our manuscript. We are grateful for the time and effort they devoted to a thorough review, and we believe that their comments and suggestions have significantly improved the clarity, rigor, and overall quality of the manuscript. All comments have been addressed point by point in the rebuttal below, and the manuscript has been revised accordingly. We hope that these revisions fully address the Editor's and Reviewers´ concerns and meet the objectives of the journal.
To facilitate tacking changes in the revised manuscript: previous edits are kept in blue, and new edits are in red.

Comments:

Reviewer #1 (Remarks to the Author):

The authors have made many changes to the manuscript in response to the reviewers' comments. However, there remain numerous points that require clarification, particularly regarding the statistical analysis, reporting of the results and interpretation of the findings.

1. The conclusions in the Abstract should be more carefully written to convey accurately the findings. The authors write "Longitudinal ophthalmic assessments revealed retinal thinning and significant reductions in RGC function, along with persistent deficits in visual acuity. Histological analysis confirmed dorsal RGC loss and late-stage changes in ON glia." What are the reductions in RGC function that are referred to here? There were no significant changes in retinal functional measures in Figure 2. Visual acuity deficits were observed at 3 months but not 6 or 9 months, so the characterization of these deficits as 'persistent' is questionable. The changes in ON glia were only for astrocytes, not microglia, so it would be more precise to state this as 'changes in ON astrocytes'.

R: The authors thank Reviewer 1 for carefully identifying these inconsistencies and for the opportunity to clarify and improve the manuscript. We have revised the text accordingly.

In addition, during a thorough re-examination of the electroretinogram (ERG) analysis, we identified an error in the reporting of the a-wave implicit time (see **point 3g**). The correct statistical outcome indicates a significant delay in the a-wave implicit time at 9 months post-injury. We have now corrected this throughout the manuscript, and the corresponding ERG results section, Discussion, and Abstract have been updated accordingly.

We also agree with the reviewer's comments regarding precision of terminology and interpretation of the histological findings. Accordingly, we have revised the manuscript to specify that GFAP expression in the optic nerve does not differ significantly between groups (see **point 12**), while statistically significant differences are observed in microglial activation (see **points 7 and 11**). The Abstract and main text have been updated to accurately reflect these distinctions. Finally, we have revised the description of the temporal profile of visual acuity changes to avoid the term "persistent," in accordance with the reviewer's suggestion.

53-61: *"Longitudinal ophthalmic assessments revealed transient retinal thinning and deficits in visual acuity, accompanied by a subtle delay in the a-wave implicit time of the electroretinogram (ERG) and significant reductions in RGC function at 9 months post-injury, as measured by patter ERG amplitude. Histological analysis further demonstrated dorsal RGC loss and late-stage regionally restricted microglia activation in the ON. Together, these findings demonstrate that the thirteen-lined ground squirrel recapitulates key aspects of the heterogeneous*

systemic, behavioral, and visual dysfunction following TBI, establishing it as a promising model for neurotrauma and vision loss."

2. The housing conditions are not detailed for the subset of ground squirrels that were moved to the hibernaculum to allow hibernation (in support of collecting weight data in Figure 1?). The alterations to vivarium temperatures (shown in Figure 1) were not described in the housing section.

R: The authors thank reviewer 1 for pointing out this absence of housing details, we have now included detailed description accordingly in material and methods section:

506-512: *Animals were then implanted with subcutaneous temperature transponders for continuous monitoring of core body temperature and housed individually under standard laboratory conditions with seasonally adjusted light–dark cycles designed to replicate natural photoperiods in Wisconsin (Figure 1B). Briefly, environmental temperature (gray bars) was gradually increased from 60 ± 4 °F in December to 72 ± 4 °F by late spring–summer, with incremental changes of ~1 °F each two-week period. In parallel, the photoperiod (orange trace) was extended from 8.5–9 hours of light in December–January to approximately 15 hours in June, increasing by ~1.5 hours per month."*

535-545: *"To control potential differences in body weight associated with hibernation, a subset of Thirteen-lined ground squirrels (n = 6) was allowed to enter hibernation in the fall after exhibiting signs of spontaneous torpor. These animals were transferred by veterinary personnel from standard housing conditions (ambient room temperature) to a dedicated hibernaculum maintained at 4 °C, as reflected in Figure 1C. Following transfer, animals were monitored daily, and after three consecutive days in stable torpor (confirmed by a body temperature of ~4 °C) they were placed in individual containers with appropriate bedding, without access to food or water, consistent with standard hibernation protocols. Health status was assessed twice weekly, and body weight was recorded periodically throughout the hibernation period. Importantly, these animals were used exclusively for body weight monitoring and were not subjected to any experimental procedures or included in subsequent analyses."*

3. The statistical analysis section and reporting within the text/legends is not complete. a) It would be easier for the reader to follow if references were provided to the data figures for each test described. For example, "OCT measurements of RNFL and total retinal thickness (Figs 2B and 2C) were analyzed using repeated-measures one-way ANOVA in GraphPad Prism (version 10.5.0), as the dataset included pre-injury baseline measurements and follow-up assessments 4.5- and 9-months post-injury." This would also help the authors verify that they have provided methods for each dataset.

R: The authors thank Reviewer 1 for highlighting the need for improved consistency and clarity in the reporting of statistical analyses. Statistical tests were performed for all datasets included in the manuscript. In response to this comment, we have revised the OCT Results section to explicitly include the statistical analyses, as suggested. In addition, to ensure comprehensive and easily traceable reporting, and **following Reviewer 1's recommendation (point 3c),** we have included a summary table (**Supplementary Table 1**) that compiles all statistical analyses. This table is now systematically referenced throughout the manuscript for each dataset.

169-177: Because ON injury is expected to reduce the number of RGCs, we examined thinning of the retinal nerve fiber layer (RNFL) as an indicator of axonal loss. However, RNFL thinning was not anticipated within the timeframe of this study, as previous studies have shown that retinal thickness in thirteen-lined ground squirrels remains stable with aging[47]. Optical coherence tomography (OCT) measurements were therefore focused on the visual streak, a specialized retinal region characterized by the highest density of cones and RGCs. Importantly, due to the horizontal

orientation of the ON head (ONH), which effectively divides the retina into superior and inferior hemiretinas, a large proportion of RGC axons from regions below the ONH converge through the visual streak[14-19]. This makes it a particularly reliable and anatomically consistent region for detecting changes in RNFL thickness in the thirteen-lined ground squirrel[14].

*Analysis of the visual streak revealed that RNFL thickness (**Figure 2A, yellow arrow**) was not significantly reduced in injured animals at 9 months post-CHIMERA compared to the pre-injury (baseline) levels from the same animals (**Figure 2B;** repeated-measures one-way ANOVA, F(1.389, 12.50) = 2.075, p = 0.1730; **Supplementary table 1**). In contrast, total retinal thickness was significantly reduced at 4.5 months post-injury (**Figure 2C;** repeated-measures one-way ANOVA, F(1.946, 17.51) = 15.34, p =0.0002; Tukey's post hoc for multiple comparisons, p = 0.0014; **Supplementary table 1**). By 9 months post-injury, retinal thickness was no longer significantly different from baseline but was significantly increased relative to the 4.5-month time point (**Figure 2C**; Tukey's post hoc for multiple comparisons, p = 0.0079**; Supplementary table 1**).*

b) In the previous sentence, statistical software is referenced for this one test, but not for any of the other tests. If all tests were run in GraphPad Prism v 10.5.0, this statement should stand alone, not in association with one test.

R: The authors thank reviewer 1 for requesting consistency and clarity. Because all statistical analysis were performed using the same software and version, we have now stated it in the Statistics section in Material and methods:

825-828: *"Statistical analyses were conducted using GraphPad Prism (version 10.5.0) except for the indocyanine green (ICG) vascular intensity (Levene's test calculator and Statistics Kingdom 2017). A comprehensive summary of the statistical tests applied, including results and significance levels, is presented in **Supplementary Table 1**."*

c) ANOVA results are not consistently reported. The authors should report all ANOVA F test results and p values. F tests should be reported as F(x,y)=z, p=xx or Fx,y=z, p=xx. For 2-way ANOVAs or 1-way ANOVAs with repeated measures, both main effect and interaction ANOVA results should be provided. This can be done in the results text, the figure legends or in a table.

R: The authors thank Reviewer 1 for emphasizing the importance of consistent and complete reporting of ANOVA results. In accordance with this recommendation, results section has been revised to refer to the ANOVA outputs (F values, and corresponding *p* values) where appropriate. To improve clarity, we have compiled all statistical analyses, including full ANOVA outputs (F values, and corresponding *p* values) and post hoc tests results, in a summary table (**Supplementary Table 1**) for all datasets analyzed in this study.

d) Post-hoc t-tests used for ANOVAs are not described. Consideration should be given to correcting for multiple comparisons.

R: The authors thank Reviewer 1 for highlighting the need for clearer reporting of post hoc analyses and multiple-comparison corrections. In response, we have now specified the post hoc tests used following ANOVA in the Statistical Analysis section of Materials and Methods. These tests, along with the applied multiple-comparison corrections, are now systematically referenced to results section and detailed in the **Supplementary Table 1.**

e) Lines 167-169, the authors state that RNFL thickness "decreased progressively with time" despite no

significance at either time point relative to baseline and no notable change between 4.5 and 9 months. This is an overinterpretation. Further, no ANOVA results are reported. (see point c).

R: The authors thank Reviewer 1 for identifying this overinterpretation and inconsistency. We have revised the text to remove the statement that RNFL thickness "decreased progressively with time," as this was not supported by statistically significant differences (see **point 3a**). In addition, the corresponding ANOVA results are now explicitly reported, as addressed in **point 3a.** Following the recommendation in **point 3c,** all statistical details are also compiled in **Supplementary Table 1**, which is now referenced in the revised text for clarity and completeness.

f) Authors should be clear about whether p values reported are for the ANOVA or the posthoc tests. For example, on page 5 the authors write "In contrast, total retinal thickness was significantly reduced at 4.5 months post-injury (Figure 2C; p=0.0014; repeated-measures ANOVA, n=10 eyes). By 9 months post-injury, retinal thickness was no longer significantly different from baseline but was significantly increased relative to the 4.5-month time point (p=0.008) Figure 2C; repeated-measures ANOVA, F=15.34, p=0.0002)," This is confusing, because the there should only be one repeated measures ANOVA run for Figure 2C, which should be reported (F test and p value for the ANOVA) prior to reporting posthoc t-test p values. It is unclear whether the two different p values refer to the ANOVA or to the posthoc tests.

R: The authors thank Reviewer 1 for highlighting the need for clearer distinction between ANOVA and post hoc test results. We have revised the text to ensure that ANOVA results (including F values and corresponding *p* values) are reported prior to post hoc comparisons, and that *p* values are explicitly attributed to either the overall ANOVA or the post hoc tests. This specific issue has been corrected in the revised Results section (see **point 3a**). In addition, and following the recommendation in **point 3c**, all statistical details are systematically compiled in **Supplementary Table 1**, which is now referenced throughout the manuscript for clarity and consistency.

g) It is unclear how data in Figure 2 F-I were analyzed, despite a reference to no "significant" changes on line 186, which implies statistical analyses. The statistical tests should be described in the Statistical analysis section and the results reported (i.e. non-significant ANOVAs for main/interaction effects).

R: The authors thank Reviewer 1 for highlighting the need for clearer reporting of statistical analyses. We have revised the statistical approach and confirm that significant changes in implicit time are present, while no significant effects were detected for amplitude measures. Accordingly, the Results and discussion sections (see below) and figures have been updated to reflect these findings, and the corresponding statistical tests are now explicitly described. Given that amplitude and implicit time were analyzed across multiple stimulus conditions, each requiring separate statistical testing, we have centralized all detailed results, including ANOVA outputs and post hoc comparisons, in **Supplementary Table 1**, which is now systematically referenced throughout the Results section.

199-211: *"Neither the ERG a-wave nor the b-wave showed significant changes in amplitude (**Figure 2F-G**) in CHIMERA-injured animals (n = 10 eyes, 5 animals) compared with age-matched naïve controls at the 4.5-month (n = 8 eyes, 4 animals) and 9- month (n=12 eyes, 6 animals) post-injury time points (non-significant ordinary one-way ANOVA and Tukey's post hoc for multiple comparisons test, see **Supplementary table 1**). However, implicit time for a-wave for most of the stimuli presented, and only punctually in b-wave (**Figure 2H-I**), showed significant increase at 9 month post-CHIMERA respect to 4.5 months post-CHIMERA (ordinary one-way ANOVA and Tukey's post hoc for multiple comparisons test, see **Supplementary table 1** for details). Regarding implicit time changes, we also observed sporadic differences between naïve, 4.5-month and 9-month groups, however, these were of much smaller magnitude and less consistent than those observed following CHIMERA injury (see **Supplementary table 1***

for details). Together, these results indicate that CHIMERA injury does not measurably affect outer retinal response amplitude, but is associated with a subtle, time-dependent slowing of phototransduction kinetics.

325-329: *"ERG recordings showed no deficits in photoreceptor and bipolar cell response amplitude, but is associated with a subtle, time-dependent slowing of phototransduction kinetics, which may reflect mild alterations in photoreceptor response dynamics and/or early changes in post-receptor signal transmission without overt synaptic or cellular loss."*

h) There is no description of the statistical analysis for data in Fig 2 M-O.

R: The authors thank reviewer 1 for requesting consistency and clarity. In addition to the **Supplementary Table 1**, we have included the following sentence:

216-220: *"Post-injury animals showed no frequency-dependent changes in response amplitude compared with naïve animals even at 30Hz (**Figure 2M-O**; Ordinary One-way ANOVA; 5Hz: $F(3, 36)=1.221$, $p=0.3334$; 15Hz: $F(3, 36)=0.5753$, $p=0.6350$; 30Hz: $F(3, 36)=0.3016$, $p=0.8240$, **Supplementary table 1**), indicating that cone responses are not impaired by the mild closed head impact used in this study.*

i) On page 6, the authors' statement that "This reduction in OMR persisted at 6- (1.26 ± 0.32 cyc/deg) and 9-months (1.36 ± 0.25 cyc/deg) post-injury" is not supported by the statistical analysis which shows no difference, as they report: "… although the difference at these later time points no longer reached statistical significance." Given the very small n for this study, the authors must be more cautious about overinterpreting nonsignificant shifts in distribution. The title of this section (lines 200-201) should also be modified accordingly.

R: The authors thank Reviewer 1 for identifying this inconsistency. We have revised the text to remove the unsupported interpretation of a sustained reduction in OMR at later time points, and the section title has been accordingly modified to better reflect the statistical outcomes (see below). In addition, this section has been updated to consistently report all statistical analyses, including non-significant results, in line with the reviewer's recommendation (see revised manuscript).

228-207: *"The difference in visual acuity at 6- (1.26 ± 0.32 cyc/deg) and 9-months (1.36 ± 0.25 cyc/deg) post-injury no longer reached statistical significance…"*

222: *"CHIMERA-Induced TBI Causes Subtle Deficits in Visual Acuity Measured by Optomotor Response"*

j) On page 7, in describing the PERG data, the authors describe one posthoc result, then the overall ANOVA, then another posthoc result. It would be clearer to first report the overall ANOVA, and then the posthoc comparisons.

R: The authors thank Reviewer 1 for highlighting the need for clearer and more consistent reporting of statistical results. We have revised the PERG results paragraph to ensure that statistical outcomes are presented in a logical order, first reporting the overall ANOVA results followed by the corresponding post hoc comparisons. Where appropriate, we now also refer to **Supplementary Table 1** for complete statistical details, in accordance with the standardized reporting format used throughout the manuscript:

239-251: *"To corroborate the visual deficits observed with the optomotor reflex, we also measured pattern electroretinography (PERG) in the same animals before injury and at 3-, 6-, and 9-months post-injury to track longitudinal changes in retinal function. PERG provides a measure of RGC activity, with reductions in amplitude*

reflecting loss of RGC function. At 9 months post-injury, TBI-exposed thirteen-lined ground squirrels showed a significant reduction in PERG amplitude (50.3 ± 22.5 µV, n = 10 eyes) compared to their pre-injury baseline (81.6 ± 24.3 µV, n = 10 eyes; **Figure 3C-D**). Repeated-measures one-way ANOVA confirmed a significant effect over time ($F(2.383, 21.45)=3.977$, $p=0.0281$), with Tukey's post hoc for multiple comparison analysis indicating that the reduction in amplitude occurred between 6- and 9-months post injury ($p=0.0345$, **Supplementary table 1**). In contrast, the implicit time of the P50 peak in the PERG response did not change significantly from 30.2 ± 4.0 ms at baseline in the naïve condition to 33.7 ± 2.8 ms at 9 months post-injury (**Figure 3E**; Repeated-Measures one-way ANOVA, $F(2.353, 21.18)=2.613$, $p=0.0894$; **Supplementary table 1**).

k) The statistical analysis section says "Retinal vasculature parameters were compared between baseline pre-injury images and 9 months post-CHIMERA injury using Student's t tests." but Figure and Figure legends describe the control group as naïve not pre-injury. There is also a redundant sentence fragment in this part of the statistical analysis section.

R: The authors thank the reviewer for pointing out this inconsistency, and have corrected it in the text. The sentence fragment has been removed from the text.

785-787: *"Retinal vasculature parameters, MC density (Iba1 cells per unit area) in retina and ONs, glial fibrillary acidic protein (GFAP) integrated density, and the weight of different organs (spleen and kidneys), were compared between naïve images and 9 months post-CHIMERA injury using unpaired two-tailed Student's t tests."*

l) Please specify in the Statistical Analysis section whether student's t-tests were 2-tailed or 1-tailed, paired or unpaired. Then this information does not need to be provided in the Results text.

R: The authors thank Reviewer 1 for highlighting the need for clearer specification of statistical tests. All Student's *t*-tests performed in this study were unpaired and two-tailed. This information has now been explicitly stated in the Statistical Analysis section of the Materials and Methods, where the tests used for each dataset are described (see below). For consistency in reporting, statistical tests are also indicated in the Results section (as done for ANOVA analyses), and all details are systematically compiled in **Supplementary Table 1**.

824-855: *"Statistical Analysis*

All statistical analyses were conducted using GraphPad Prism (version 10.5.0), except for the indocyanine green (ICG) vascular intensity (Levene's test calculator and Statistics Kingdom 2017). A comprehensive summary of the statistical tests applied, including results and significance levels, is presented in ***Supplementary Table 1****. OCT measurements of RNFL and total retinal thickness were analyzed using repeated-measures one-way ANOVA and Tukey´s post hoc test for multiple comparisons in, as the dataset included pre-injury baseline measurements and follow-up assessments 4.5- and 9-months post-injury. Amplitude and implicit time of the ERG a- and b-waves, as well as flicker response amplitudes (5, 15, and 30 Hz), were analyzed using ordinary one-way ANOVA followed by Tukey's post hoc test for multiple comparisons, comparing age-matched naïve control animals with CHIMERA-exposed animals. OMR visual acuity measurements were analyzed using ordinary one-way ANOVA and Tukey´s post hoc test for multiple comparisons, as age-matched naive control animals and CHIMERA-exposed animals were compared. Repeated-measures analysis was not applied because the naïve control animals were not longitudinally matched to the CHIMERA cohort; control animals were assessed at 0 and 9 months, whereas CHIMERA animals were assessed at 3-, 6-, and 9-months post-injury. Pattern ERG amplitudes and implicit times were analyzed using repeated-measures one-way ANOVA and Tukey´s post hoc test for multiple comparisons, consistent with the inclusion of pre-injury baseline measurements and follow-up measurements at 4.5- and 9-months post-injury.*

Retinal vasculature parameters, MC density (Iba1 cells per unit area) in retina and ONs, glial fibrillary acidic protein (GFAP) integrated density, and the weight of different organs (spleen and kidneys), were compared between naïve images and 9 months post-CHIMERA injury using unpaired two-tailed Student's t tests. The ICG vascular intensity histogram and confidence intervals were plotted using Levene's test calculator [Internet] and Statistics Kingdom 2017 [cited 18 September 2025] (Available from: https://statskingdom.com/230var_levenes.html and verified using the R software package). RGC (RBPMS) density in the retina was initially analyzed using ordinary one-way ANOVA with Tukey's post hoc test for multiple comparisons. However, because RGC density differs between dorsal and ventral retina[14] and this was not the focus of the present study, direct comparisons between naïve and 9-month post-CHIMERA groups were performed using unpaired two-tailed Student's t-tests. Microglial activation (Iba1/CD68 co-localization) was analyzed using Fisher's exact test by comparing the proportion of ONs exhibiting activated microglia between naïve and CHIMERA-treated groups."

m) The authors, in their response to the previous critique that two eyes or nerves in the same animal are not independent measures, provide a statement in their 'definition of n' that "where applicable, bilateral measure were analyzed using repeated-measures ANOVA designs". This approach does not seem to have been incorporated into any of the analyses. Further, the authors statement that the injury is "equivalent" to both eyes and nerves of one animal is not supported by any evidence and is contradicted by their statement that the TBI can "differentially affect each visual pathway". Further, if the injury (mechanically) is equivalent to both sides of the visual system, as the authors suggest, this is in support of the necessity of treating the left and right samples as repeated measures rather than independent samples. That is, the responses within an animal may be more similar than those across animals. This can be tested statistically, but the fact that some animals were sampled once (R or L) while others were sampled bilaterally (R and L) as revealed in the supplementary Excel file provided with this revision, makes testing this assumption virtually impossible. The lack of systematic sampling is a limitation of the study design that needs to be included.

R: The authors thank Reviewer 1 for this thoughtful comment regarding the treatment of bilateral samples and the statistical approach. We agree that, in principle, when measurements from both sides of the same animal are systematically collected, a repeated-measures design may be appropriate to account for potential intra-animal correlations.

However, we respectfully clarify that in the context of the CHIMERA model, it is important to distinguish between the **mechanical delivery of the injury** and the **resulting biological response**. While the impact is applied at the midline -therefore being mechanically comparable with respect to both optic nerves- this does not ensure equivalent biological outcomes. In traumatic brain injury models, variability arises from subtle anatomical asymmetries, differences in force transmission, and local tissue susceptibility. Consequently, the extent, localization, and progression of axonal damage, as well as associated cellular responses, may differ between the left and right optic nerves within the same animal.

Consistent with this, our data show heterogeneous patterns of degeneration and microglial activation between sides, supporting the notion that bilateral measurements cannot be assumed to be equivalent or strongly correlated. Therefore, treating each optic nerve as an independent experimental unit reflects the biological variability inherent to this model rather than a contradiction of the mechanical setup.

Importantly, we acknowledge that bilateral sampling was performed systematically across all animals or analyses, however, for some ex vivo analysis not all samples from the same individual undergo same staining combination. This heterogeneity precludes the consistent application of repeated-measures statistical approaches and limits

the ability to formally assess intra-animal dependence. We agree that this represents a limitation of the study design. Accordingly, we have revised the manuscript to clarify this point and now explicitly state:

475-482: *"In this study, although the traumatic impact was delivered at the head midline, the biological outcome may differ between sides due to inherent variability in TBI models. Thus, for all groups, both eyes and ONs from each animal were analyzed and treated as independent experimental units (OCT, ERG, PERG, and ex vivo anatomical analysis), except for the behavioral assessment (OMR), where each animal was an experimental unit. While bilateral sampling was performed, staining combinations were not applied systematically across all ex-vivo analyses, precluding the use of repeated-measures statistical approaches and limiting the assessment of intra-animal correlations."*

4. In the legend for Figure 2, the box and whisker plots are described as "Error bars represent the 90th (top) and 25th (bottom)". This is a nonstandard description and seems incomplete and in error. The middle line and top and bottom of the box should be defined (often these represent median and quartiles), and the whiskers (error bars) should be defined (often these represent the 95th percentile, or maybe the 90th percentile in this case?). This explanation should be included in each figure containing box plots. Alternatively, the explanation could be in the Statistical Analysis section to describe how all box plots are represented.

R: The authors thank reviewer 1 for pointing out this error and for requesting consistency and clarity. We have now removed this sentence from the Fig. 2 legend. Accordingly, we have now edited figure legends, and included a corrected description for all box plots in the Statistical Analysis section as recommended:

856-860: *Data are presented as box plots (GraphPad Prism, version 10.5.0), in which the central line represents the median and the upper and lower box boundaries indicate the upper and lower quartiles, respectively. Whiskers extend to the 90th (upper) and 10th (lower) percentiles. Individual data points are shown as dots. Statistical significance is indicated as follows: $p \le 0.05$ (*), $p \le 0.01$ (**), $p \le 0.001$ (***), and $p \le 0.0001$ (****).*

5. On page 5-6, the authors state "By 9 months post-injury, retinal thickness was no longer significantly different from baseline but was significantly increased relative to the 4.5-month time point …suggesting that prolonged retinal inflammation may contribute to increased cellular thickness at this later stage." It is unclear how any inference about inflammation can be drawn from retinal thickness measurements. This speculative comment should be removed from the Results.

R: The authors thank the reviewer for pointing out this speculation, and have removed it from the text.

6. Statements (page 8) such as "This likely reflects the fact that microglia are primarily recruited to sites of active degeneration and debris clearance. Following the initial injury and subsequent wave of neurodegeneration, the inflammatory response diminishes in regions where RGCs have already been lost, leading to a reduced microglial presence over time" are speculative interpretation and belong in the Discussion, not the Results.

R: The authors thank the reviewer for pointing out this speculation, and have moved it to the Discussion.

340-347: *"Ex vivo confocal imaging of retinal flat mounts revealed peripheral RGC loss confirming these observations. However, although we could not reliably detect significant RGC degeneration within the visual streak - likely due to the high density of RGCs in this region[14,19] which may obscure subtle RGC loss – we observed a trend toward increased microglial cell density in the visual streak. This likely reflects the fact that microglia are primarily recruited to sites of local axonal degeneration and debris clearance. Following the initial injury and subsequent*

wave of neurodegeneration, the inflammatory response diminishes in regions where RGCs have already been lost, leading to a reduced microglial presence over time."

7. Similarly, "certain ON sections display microglial activation, possibly associated with localized axonal degeneration." The latter part of this sentence is speculation that could be provided in the Discussion but not the Results.

R: The authors thank the reviewer for pointing out this speculation. We have provided a new analysis to support significant microglial activation in ONs subjected to CHIMERA (see **point 11**). We have accordingly edited the sentence in results while moving the speculative paragraph to the Discussion.

307-311: *"Nonetheless, IBA1 and CD68 co-localization indicate that 71% of ONs subjected to CHIMERA exhibit microglial activation, whereas none of the control ONs showed an increased CD68 signal (5/7 vs. 0/6 ONs in CHIMERA and control groups, respectively; Fisher´s exact test: P value=0.0210*, Two-sided; **Supplementary table 1**)."*

350-352: *"In this context, the increased prevalence of localized microglial activation (Iba1/CD68 co-localization) in ONs following CHIMERA is likely associated with ongoing focal axonal degeneration."*

8. The statement "TBI-exposed thirteen-lined ground squirrels exhibited long-term deficits in RGC function, as evidenced by reduced pattern ERG amplitudes and delayed implicit times" on page 9 is not supported by the data, which showed no change in implicit times. Further, the PERG data shows a delayed decrease in RGC function, which is somewhat different than 'long-term deficits' which could be interpreted as deficits that persist for a long time.

R: The authors thank the reviewer for making this clarification, and have corrected the text accordingly. Discussion of delayed implicit times has been removed:

322-324: *"TBI-exposed thirteen-lined ground squirrels exhibited persistent deficits in RGC function, as evidenced by reduced pattern ERG amplitudes, consistent with findings from other studies of blast-induced injury[51,52]."*

9. The statement that " in vivo anatomical evaluation using OCT revealed a gradual thinning of the RNFL" is not supported by the data, which 1) does not show a statistically significant change and 2) shows an equivalent median at 4.5 and 9 months. There is no acute data, so the authors do not know if this nonsignificant decrease was 'gradual'.

R: The authors thank reviewer 1 for making this clarification, and have corrected the text accordingly.

333-335: *"In agreement with these functional and behavioral results, in vivo anatomical evaluation using OCT revealed a significant thinning of the total retinal thickness at 4.5 months post-injury and a subsequent increase in thickness at 9 months,…"*

10. The statement "…total retinal thickness was revealed to be significant at 4.5 months post-injury and a subsequent increase in thickness at 9 months, possibly reflecting an immune cell-mediated response. This degeneration…" is also highly speculative. What evidence is there of immune cell mediated increase in retinal thickness? The authors data show no change in retinal glia. Further, what evidence is there that the decreased

retinal thickness represents 'degeneration', if the change is transient? RGC dysfunction does not equate to degeneration.

R: The authors thank Reviewer 1 for raising this important point and for the opportunity to clarify our interpretation. We acknowledge that the term "immune cell-mediated response" was inaccurate and have corrected this to "glia-mediated response" in the revised manuscript.

We agree that our previous wording was overly speculative and have revised the text accordingly. Although we did not detect a significant increase in microglial cell numbers, inflammatory responses following optic nerve injury are not limited to changes in microglial density. Traumatic optic nerve injury is well known to induce a complex neuroinflammatory response involving activation of resident microglia, astrocytes, and Müller glia, as well as the release of cytokines and recruitment of peripheral immune cells .

Importantly, experimental models of optic nerve injury have demonstrated that retinal inflammation and gliosis occur rapidly after injury, including upregulation of inflammatory mediators (e.g., TNF-α, IL-1β) and gliosis markers such as GFAP and Iba1, even in the absence of overt changes in cell number . In addition, reactive gliosis of Müller cells and astrocytes is a hallmark of retinal and optic nerve injury and can lead to structural remodeling of the retina .

Notably, inflammation-associated retinal thickening has been described in models of optic nerve and retinal injury, where early inflammatory edema and glial activation can transiently increase retinal thickness and mask underlying neurodegenerative changes . These findings support the concept that changes in retinal thickness may reflect a dynamic balance between degeneration and inflammation-driven structural remodeling.

In this context, the transient reduction in retinal thickness observed at 4.5 months may reflect early structural alterations associated with RGC dysfunction or stress. However, because this effect is not sustained, we have revised the manuscript to avoid interpreting it as definitive degeneration. The subsequent return to baseline thickness at 9 months may instead reflect compensatory or reactive glial responses, including Müller cell or astrocyte hypertrophy and/or inflammation-associated tissue remodeling.

333-338: *"In agreement with these functional and behavioral results, in vivo anatomical evaluation using OCT revealed a significant thinning of the total retinal thickness at 4.5 months post-injury and a subsequent increase in thickness at 9 months, possibly reflecting dynamic structural remodeling processes rather than sustained degeneration, potentially involving glial responses such as Müller cell or astrocyte reactivity, as well as inflammation-associated tissue changes that can temporarily influence retinal thickness."*

11. The statement "These events were accompanied with subtle alterations in retinal vascular branching and changes in microglial markers in the ON" is not accurate, as there were no changes in microglial markers (Iba1) in the retina or ON. The CD68 labeling was reported anecdotally. If the authors wish to make a claim about changes in microglial in the ON, they need to perform a more complete examination of CD68 immunolabeling.

R: The authors thank reviewer 1 for requesting statistical evidence for our observations. We have now quantified as the proportion of ONs exhibiting microglial activation versus those without detectable activation in both experimental and control groups. We performed the Fisher exact test for presence of activated Microglial cells in the ON analyzed. Accordingly, we have included details in material and methods, statistics and results sections as well as its corresponding plot in Fig. 5C. This test provides evidence of microglial cell activation in 71% (n=5/7) of

the ONs at 9 months after CHIMERA, while 0% (n=0/6) of ONs had activated microglial cells in Naïve animals (also in **point 7**).

808-816: Microglial activation in the ON was evaluated by assessing the co-localization of Iba1 and CD68 immunoreactivity. Focal CD68-positive microglial activation was clearly detected in specific branches in most ONs subjected to CHIMERA; however, widespread activation across the entire ON section was not detected. Given the relatively large diameter and complex architecture of the ON of the thirteen-lined ground squirrel, compared with those of mice or rats, only a subset of sections was analyzed. Consequently, additional localized regions of degeneration and associated microglial activation may have been underrepresented. Therefore, microglial activation was quantified as the proportion of ONs exhibiting microglial activation versus those without detectable activation in both experimental and control groups.

307-311: *"Nonetheless, IBA1 and CD68 co-localization indicate that 71% of ONs subjected to CHIMERA exhibit microglial activation, whereas none of the control ONs showed an increased CD68 signal (5/7 vs. 0/6 ONs in CHIMERA and control groups, respectively; two-sided Fisher´s exact test, p=0.0210; **Supplementary table 1**)."*

853-855: *"Microglial activation (Iba1/CD68 co-localization) was analyzed using Fisher's exact test by comparing the proportion of ONs exhibiting activated microglia between naïve and CHIMERA-treated groups."*

12. It is unclear how the data in this study are consistent with reports of axonal injury in rodent TBI (lines 312-315), since no assessment of axonal pathology was performed and GFAP expression decreased, which is opposite of the typical response to white matter injury.

R. The authors thank Reviewer 1 for this important observation and for the careful evaluation of our results. We agree that our initial analysis of GFAP expression in the optic nerve was not fully aligned with the expected response to white matter injury described in rodent traumatic brain injury studies.

In response, we have reanalyzed the GFAP integrated intensity using a more stringent and controlled approach. Specifically, quantification was restricted to a defined 0.2 × 1 mm region of interest in naïve optic nerves and those subjected to CHIMERA at 9 months. To minimize technical variability, we included only samples that were sectioned on the same day, mounted on the same slide, and processed simultaneously during immunostaining. As a result, a subset of previously included optic nerves was excluded to ensure appropriate comparability.
Importantly, this revised analysis led to a change in the statistical outcome: no significant differences in GFAP integrated intensity were detected between groups using an unpaired two-tailed t-test. These updated results have been incorporated into the revised manuscript, including corresponding changes in the Methods, Results, Figures, Abstract, and Conclusions. The revised dataset has been included in the updated Excel file (row data) provided for review, and the new statistical outputs are reported in **Supplementary Table 1**.

817-822: *"Quantification of GFAP immunoreactivity in the optic nerve was performed using ImageJ (FIJI, NIH) by measuring integrated density normalized to the corresponding optic nerve area. To minimize technical variability, only samples that were sectioned on the same day, mounted on the same slide, and processed simultaneously during immunostaining were included. For each optic nerve, a region of interest (ROI) measuring 0.2 × 1 mm was defined, and fluorescence intensity was quantified using the integrated density function."*

311-314: *"In contrast, glial fibrillary acidic protein (GFAP) expression did not differ significantly between groups **(Figure 5D-E)** as quantified in comparable regions of interest adjacent to the ONH **(Figure 5F-G;** unpaired two-*

tailed student's t test, p=0,6757; n=3 and 6 ONs for naïve and 9-months post-CHIMERA, respectively; ***Supplementary table 1****).*

358-361: *"In addition, although previous studies have reported increased GFAP immunoreactivity following TBI[62–64], we did not observe differences in GFAP levels in ON astrocytes at 9 months post-injury, which may reflect a resolution of astrocytic reactivity at chronic time points, highlighting the dynamic and heterogeneous nature of glial remodeling following TBI."*

13. Given the authors admission that availability of the ground squirrels and their protected status constrained their experimental design, these considerations should be provided as limitations to the model in the Discussion.

R: The authors thank the reviewer for suggesting this additional limitation be added to the text.

441-446: *"Due to the thirteen-lined ground squirrels' classification as a USDA-protected species, the experimental design required careful optimization to minimize animal numbers and experimental attempts while still allowing determination of whether TBI induces measurable alterations in the visual pathway. Thus, the species' protected status may limit further studies in this model. The ground squirrel is native primarily to the central United States and parts of Canada, and does not receive protected status in other countries."*

Reviewer #2 (Remarks to the Author):

I am happy with the revisions made and have no further comments.

R: The authors thank reviewer 2 for their careful evaluation of our manuscript.

Reviewer #3 (Remarks to the Author):

I thank the Authors for the comprehensive responses to all the Editor's and Reviewers' comments. I am grateful for the Reviewers for catching things I missed.
The Rebuttal responses to MY particular concerns have been met in the revision.
If the other Reviewers feel the same way, I consider this Revision to be a success worthy of publication.
Nevertheless there are two small points:

1. The word "scull" should be spelled "skull".

R: The authors thank reviewer 3 for pointing out this typo and have corrected it in the text:

638: *"such as the skull thickness"*

2. The new text at the Revision's Lines 571-2 should reads: "...despite multiple publications using mice that report damage from one impact(92-96)" [add "using mice"]. It's for a different paper I'm sure, but I wonder how much the difference in skull morphology plays in the differential susceptibility to a single impact.

R: The authors thank reviewer 3 for bringing up this interesting point and have made the suggested changes to the text.

638-641: *"None of these conditions produced detectable pathological changes in the visual pathway probably due to the CHIMERA model, the injury location, the animal model and/or the animal anatomy, such as the skull thickness, despite multiple publications using mice that report damage from one impact[92–96]."*
