# Supplementary material for: Impact-acceleration head injury results in optic neuropathy in the thirteen-lined ground squirrel
Source: Commun Biol. 2026 Jun 9;9:1217. doi: 10.1038/s42003-026-10397-4 (PMC13586358; doi:10.1038/s42003-026-10397-4)
Supplement: Supplementary file 4 [file 42003_2026_10397_MOESM2_ESM.docx]

**Description of Additional Supplementary Files**

**File name:** Supplementary Data

**Description:** This file contains the raw numerical data underlying all plots presented in the main and supplementary figures. Data are provided for each individual animal and include sex as a biological variable.
